# Post-transport recovery trajectory of the canine gut microbiome and metabolome

**DOI:** 10.1186/s40104-026-01385-z

**Published:** 2026-04-12

**Authors:** Yang Lyu, Chunxia Su, Keying Sun, Yuwei Wang, Lingna Zhang, Junning Pu, Caimei Wu, David Thomas, Lianqiang Che

**Affiliations:** 1 Key Laboratory of Animal Disease-Resistance Nutrition, Ministry of Education; Animal Nutrition Institute, Sichuan Agricultural University, Chengdu, 611130 China; 2 College of Laboratory Medicine, Chengdu Medical College, Chengdu, 610500 China; 3 College of Animal Science, South China Agricultural University, Guangzhou, 510642 China; 4 School of Agriculture and Environment, Massey University, Palmerston North, New Zealand

**Keywords:** Behavioral changes, Dogs, Gut microbiota, Metabolomics, Transportation stress

## Abstract

**Background:**

Transportation induces a multisystem stress response in companion animals, yet the integrated recovery dynamics across physiological, microbial, and metabolic domains remain poorly characterized. This study comprehensively tracked the 7-day recovery trajectory in dogs following road transport by analyzing clinical parameters, fecal microbiome and metabolome.

**Results:**

Time-dependent changes were observed across domains, with differing temporal patterns. Fecal consistency improved rapidly, while behavioral scores exhibited a decrease followed by stabilization. Microbial alpha diversity initially decreased, with significant community restructuring persisting throughout recovery, culminating in a new stable state distinct from the arrival (D0) state. This shift was characterized by early enrichment of *Fusobacterium* and *Clostridium sensu stricto 1*, followed by late dominance of *Erysipelatoclostridium*, contrasting with the initial post-transport (D0) community dominated by *Prevotella 9*, *Lactobacillus*, *Phascolarctobacterium*, *Anaerobiospirillum*, *Parabacteroides*, and *Prevotellaceae GA6A1* group. Metabolomic profiling confirmed a sustained metabolic shift, involving pathways in the biosynthesis of steroid and unsaturated fatty acids and the metabolism of butanoate and several amino acids. Strong cross-domain correlations linked specific microbial genera and metabolites with behavioral improvement, underscoring gut-brain axis involvement.

**Conclusion:**

By D7, several measures remained distinct from the arrival (D0) state, indicating persistent multi-system differences during the first week after transport. These findings elucidate the complex, coordinated adaptation to transport stress, highlighting ongoing clinical, microbial, and metabolic differences by D7 and providing a framework for interventions aimed at enhancing welfare and resilience in transported companion animals.

**Graphical Abstract:**

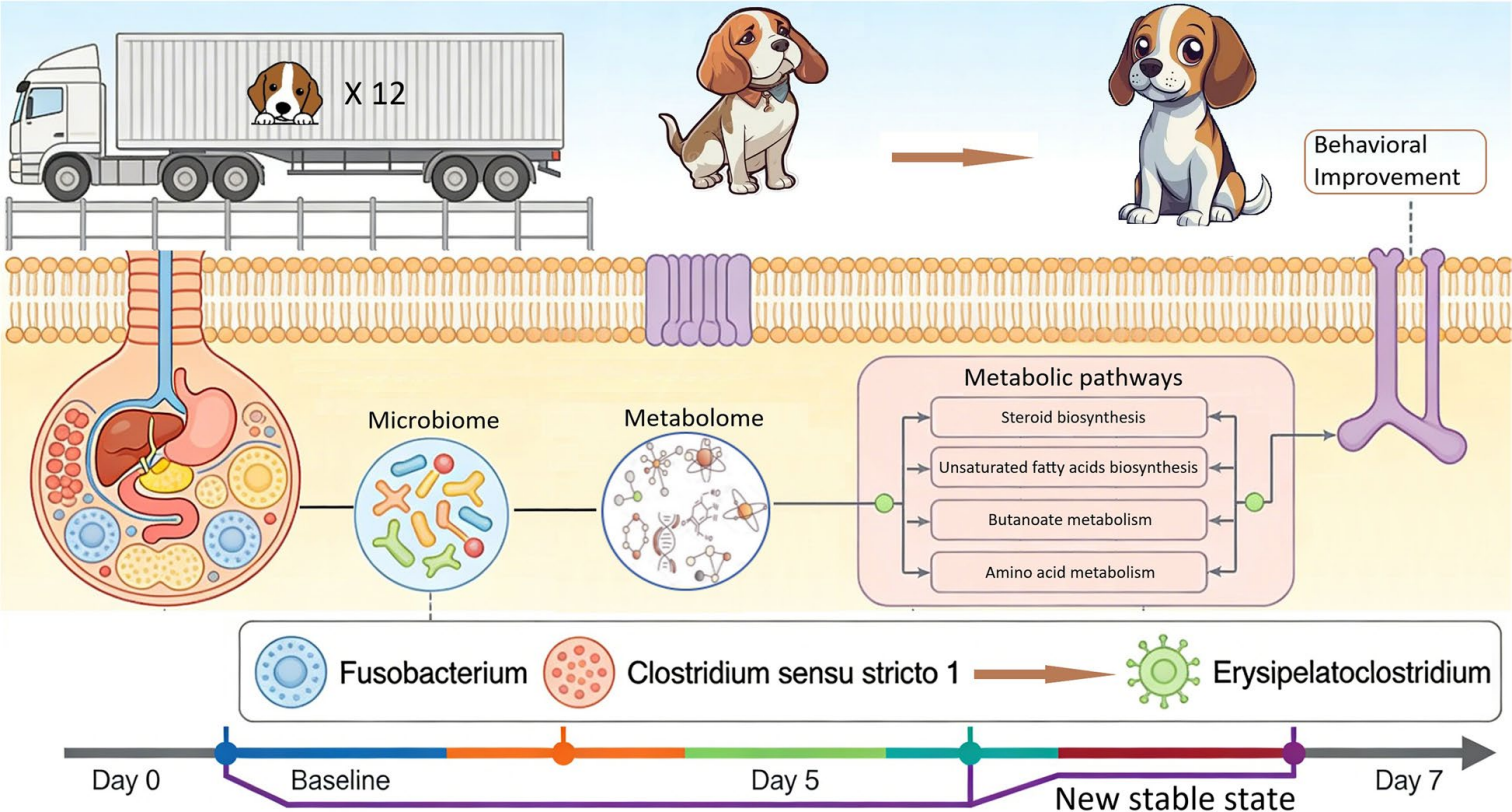

**Supplementary Information:**

The online version contains supplementary material available at 10.1186/s40104-026-01385-z.

## Introduction

The domestic dog (*Canis familiaris*) holds a distinctive role in human society, serving not only as a companion and working partner but also as an important model in biomedical research. Throughout their lives, dogs are frequently subjected to long-distance transport, whether for relocation, travelling, shows, breeding programs, or research purposes [1]. While common, such transport constitutes a major multidimensional stressor that can significantly disrupt canine welfare and physiological homeostasis [2]. Transportation stress is best understood as a composite challenge, involving confinement, social disruption, sensory overload, noise, vibration, variable temperature and humidity, and often limited access to food and water [2, 3]. Together, these factors elicit a complex stress response that may contribute to cumulative physiological burden, with serious consequences for the dog’s health, behavior, and the reliability of scientific data obtained from transported animals [2–4].

The physiological and pathological effects of transport stress in dogs are largely mediated by activation of the hypothalamic-pituitary-adrenal axis and the sympathetic-adrenal-medullary system [5, 6]. This leads to a rapid rise in catecholamines (e.g., epinephrine and norepinephrine) and glucocorticoids (mainly cortisol in dogs) [7, 8]. Although adaptive in acute scenarios, sustained elevation of these hormones can trigger immunosuppression [9], cardiovascular changes such as tachycardia and hypertension [10], a catabolic metabolic state leading to muscle loss and hyperglycemia [11], and gastrointestinal (GI) dysfunction, including altered motility, reduced secretion, and increased intestinal permeability [12]. Such GI disturbances are particularly consequential, as they form a critical junction between systemic stress and the gut ecosystem, potentially initiating microbial dysbiosis [13].

The gut microbiota, a complex community of microorganisms inhabiting the GI tract, is now recognized as a fundamental regulator of host health. This ‘microbial organ’ contributes to nutrient digestion, energy harvest, vitamin synthesis, immune regulation, and pathogen defense [14]. A healthy microbiota exhibits both stability and resilience, yet this balance is easily disrupted by external stressors via the brain-gut-microbiota axis, a bidirectional signaling network through which stress alters gut motility, secretion, and barrier function, thereby exerting selective pressure on microbial populations [15]. In turn, gut microbes influence host physiology via neurotransmitter synthesis, immunomodulation, and production of bioactive metabolites [14, 15].

During transport, stress is expected to profoundly affect the gut microbiota [16]. Altered intestinal transit time influences nutrient availability for bacteria; luminal norepinephrine can directly modulate bacterial growth and virulence; and increased gut permeability may promote bacterial translocation and inflammation [16, 17]. The resulting dysbiosis typically involves reduced microbial diversity, depletion of beneficial commensals (e.g., *Faecalibacterium* and *Lactobacillus*), and expansion of opportunistic taxa such as *Fusobacterium*. These structural shifts inevitably lead to functional changes in the microbiome’s metabolic output [15–18].

The metabolic activity of gut microbiota plays a key role in host physiology. Microbial metabolites, such as short-chain fatty acids (SCFAs) from dietary fiber fermentation, provide energy for colonocytes, exert anti-inflammatory effects, and support barrier function [15, 18]. Stress-related dysbiosis often corresponds with decreased SCFA production, worsening gut integrity loss and inflammation [16, 17]. Similarly, microbial transformation of bile acids influences lipid and glucose metabolism and immune activity via receptors such as farnesoid X receptor. Stress can unbalance this system, altering bile acid signaling [19]. Tryptophan-derived microbial metabolites (e.g., kynurenines, indole-3-acetic acid) also modulate immune and neural pathways, while oxidized fatty acids like hydroxyoctadecadienoic acid may reflect oxidative damage induced by stress [20].

Despite the acknowledged impact of transport stress on canine health, a comprehensive longitudinal assessment of microbiome-metabolome interactions during recovery is still missing. Most studies capture only acute stress markers or single time points, leaving the timeline of microbial and metabolic restoration poorly mapped. Understanding whether and how the gut ecosystem recovers after stress removal is essential for developing effective mitigation strategies. Therefore, this study aims to profile the fecal microbiome and metabolome of dogs over a 7-day recovery period following long-distance transport. We hypothesize that transport stress will induce significant microbial and metabolic disruption, manifesting as temporal changes within the initial 7 d post-arrival. These findings will provide systemic insights into the ‘stress-dysbiosis-recovery’ axis in dogs, offering biomarkers for welfare assessment and targets for interventions that enhance resilience to transport stress.

## Materials and methods

### Experimental design and sample collection

The study protocol was reviewed and approved by the Animal Care and Use Committee of Sichuan Agricultural University (Approval No. 20250519). Twelve individually-housed, healthy, unrelated adult Beagle dogs (6 males and 6 females, aged between 1 and 2 years, 11.5 ± 0.9 kg) were purchased as experimental animals (Ensiweier Biotech., Chengdu, China) for other research projects unrelated to this study, and subsequently subjected to a long-distance transport protocol by Ensiweier Biotech.

The dogs were transported over a total distance of approximately 2,080 km (from Shandong to Sichuan province, China) in a medium-sized van via highway over a 5-day period, with travel occurring only during daylight hours. They were housed individually in galvanized mesh cages with dimensions of 0.85 m × 0.72 m × 0.56 m, ensuring sufficient space for standing, turning, and lying down. The van was typically operated for an average of 7 to 9 driving hours daily, incorporating 3 short breaks. A total of 4 mandatory overnight stops were observed throughout the route. Dogs were removed from cages for rest, cage cleaning, and feeding after the van was parked each evening. Following a 1–2 h period of rest, dogs were fed ad libitum once daily with the same batch of standard commercial dry diet consumed pre-transportation (Myfoodie^®^ Beef double grain, Gambol Pet Group, China; 20 kg/pack, obtained the same batch of 500 kg). The diet was stored in sealed containers within the labeled shelf life parameters in the same carriage as dogs (internal temperature: 18–24 °C). Water was provided ad libitum throughout the journey.

No adverse clinical signs, such as gastrointestinal (e.g., diarrhea, vomiting) or feeding (e.g., anorexia, anti-feeding behaviour) issues, were observed during transportation. The general health of the dogs was assessed daily upon arrival at the facility. Monitoring included heart and respiratory rate, food intake, body weight, body condition score (on a 9-point scale) [21], fecal score (on a 5-point scale; 1 = watery liquid that can be poured; 2 = soft, unformed stool that assumes the shape of the recipient; 3 = soft, formed, moist stool; 4 = hard, formed, dry stool; 5 = hard, dry) [22], qualitative behavioral assessment (a 5-point IAABC behavioral checklist scoring system; 1 = absence; 2 = very mild; 3 = mild; 4 = modest; 5 = severe) [23], and the occurrence of vomiting or diarrhea. Behavior scoring was performed by two trained independent observers (a veterinarian and an animal keeper in other faculties) with high inter-observer reliability (intraclass correlation coefficient > 0.80). Assessments were conducted during 2–3 randomized daily on-site observation sessions (5–10 min each). Observers were blinded to the specific study hypotheses and group comparisons. The overall behavior score was calculated daily per dog by averaging the scores from all completed sessions and both observers. It combines the level of emotional expression (e.g., anxiety, fear) and the duration of vigilance/posture behavior (e.g., pacing, hiding) and activity/exploration behavior in the standardized IAABC checklist. Behaviors unrelated to this experiment were not included in the scoring (e.g., scavenges, chasing animals).

Fecal samples were collected at multiple time points: upon arrival at the facility (D0), and then on D1, D2, D3, D5, and D7 post-transport. All samples were immediately frozen at −80 °C until analysis. The dogs were maintained on the same batch of the same diet that was fed during the transport throughout the observation period. The diet was stored in sealed containers within the labeled shelf life parameters, maintained within the temperature-controlled dog stable (20–25 °C). The initial amount of food offered was calculated based on individual maintenance energy requirements according to body weight (i.e., an average daily energy intake of 110 kcal/kg [body weight]^0.75^ according to FEDIAF nutritional guideline 2025 [24]), and adjusted based on daily weight changes to maintain an ideal body condition throughout the study. Dogs were fed once daily and had free access to water.

### 16S rRNA gene sequencing and microbiome analysis

Microbial DNA was extracted from fecal samples using the QIAamp PowerFecal DNA Kit (QIAGEN, Hilden, Germany) per the manufacturer’s instructions. DNA concentration and purity were measured with a NanoDrop 2000 spectrophotometer (Thermo Scientific, Waltham, MA, USA). The V4 region of the bacterial 16S rRNA gene was amplified using primers 515 F (5′-GTGCCAGCMGCCGCGGTAA-3′) and 806R (5′-GGACTACHVGGGTWTCTAAT-3′) in a GeneAmp 9700 PCR system (Applied Biosystems, Carlsbad, CA, USA) under the following conditions: 95 °C for 3 min; 27 cycles of 95 °C for 30 s, 55 °C for 30 s, 72 °C for 45 s; and a final extension at 72 °C for 10 min. Each 20 μL PCR reaction contained 4 μL 5 × FastPfu Buffer, 2 μL dNTPs (2.5 mmol/L), 0.8 μL of each primer (5 μmol/L), 0.4 μL FastPfu Polymerase, and 10 ng template DNA. PCR products were purified from a 2% agarose gel (AxyPrep Kit, Axygen, Tewksbury, MA, USA) and quantified (QuantiFluor™-ST, Promega, Madison, WI, USA). Equimolar amplicons were pooled and sequenced (2 × 300 bp) on an Illumina MiSeq platform following standard protocols. Raw reads were processed using QIIME2 version 2024.10 with custom scripts (https://docs.qiime2.org/). The DADA2 plugin was employed for quality filtering and denoising, taxonomy assignment was performed using SILVA database. The sequencing services were provided by Baiwei Biotech Co., Ltd. (Chengdu, China).

### Non-targeted metabolomic analysis

Untargeted metabolome was analyzed by ultra-high-performance liquid chromatography coupled with tandem mass spectrometry (UPLC-MS/MS). Freeze-dried fecal samples were subjected to generic extraction using a methanol-based protocol and analyzed by Baiwei Biotech Co., Ltd. (Chengdu, China). Chromatographic separation was carried out on the UPLC system, and data were acquired in both positive and negative ionization modes. Raw mass spectrometry data were pre-processed using XCMS (v 3.9.3, https://github.com/sneumann/xcms). Raw data were processed for peak annotation and feature alignment, was conducted using CAMERA (https://github.com/sneumann/CAMERA) and metaX toolbox (v 1.4.19, https://github.com/wenbostar/metaX) implemented in R (v 4.4.0). Metabolites were identified and annotated by reference to the Human Metabolome Database (HMDB, http://www.hmdb.ca/) and Kyoto Encyclopedia of Genes and Genomes Database (KEGG, http://www.genome.jp/kegg/). The identified metabolites were further validated using an in-house metabolite fragment spectrum library provided by Baiwei Biotech.

### Statistical analysis

Clinical health parameters, including body condition, fecal quality, and behavior scores, were subjected to Friedman’s test (for overall effect) and the Bonferroni-corrected Wilcoxon signed-rank test (for post-hoc pairwise comparisons), using SPSS Statistics version 29 (IBM Corp., Armonk, NY, USA). Statistical significance was defined as a *P*-value of less than 0.05. A trend toward significance was noted for *P*-values between 0.05 and 0.10. All results are presented as mean ± standard deviation (SD). Results were illustrated using a box-plot generated by online platform Hiplot (https://hiplot.cn/; Shanghai, China).

Microbiome analysis was performed on the QIIME2 platform v2024.10. Alpha diversity was calculated using indices including Shannon, Simpson, Chao1, and phylogenetic diversity (PD). Beta diversity was assessed via Permutational Multivariate Analysis of Variance (PERMANOVA) using Bray Curtis distance, and Non-metric Multidimensional Scaling (NMDS) based on unweighted UniFrac distance. Linear Discriminant Analysis Effect Size (LEfSe) was employed to identify differentially abundant taxa from phylum to genus level between time points (LDA score > 2.0, adjusted *P* < 0.05).

For metabolic analysis, Partial Least Squares Discriminant Analysis (PLS-DA) was performed to visualize global metabolic profiles. Differential abundance analysis of metabolites was conducted between different days (e.g., D0 vs. D7) using Wilcoxon signed-rank test followed by Bonferroni’s test. Marker metabolites were selected according to a *P*-value < 0.05 and a fold change ≥ 2, with a focus on the top 15 most abundant compounds and Level 3 KEGG pathways.

The behavior, microbiome and metabolome datasets were integrated through a systematic, correlation-based approach. Spearman’s correlation coefficients between behavior scores, marker bacteria and metabolites were analyses using online platform MetaboAnalyst 6.0 (https://www.metaboanalyst.ca/; McGill University, Montreal, Canada). False discovery rate (FDR) correction was applied to adjust *P*-values for multiple comparisons. A scatter plot was generated using hyper-geometric test to illustrate the significantly impacted pathways.

## Results

### General health

Clinical monitoring confirmed the absence of gastrointestinal upset (i.e., vomiting and diarrhea) or feeding problem (i.e., anorexia and anti-feeding behaviour). Body condition scores remained within the ideal range (4.1 to 5.0) throughout the experiment (Additional file 1), and the non-significant and raw data are provided in the supplementary material (Additional files 1, 2 and 3).

**Fig. 1:**
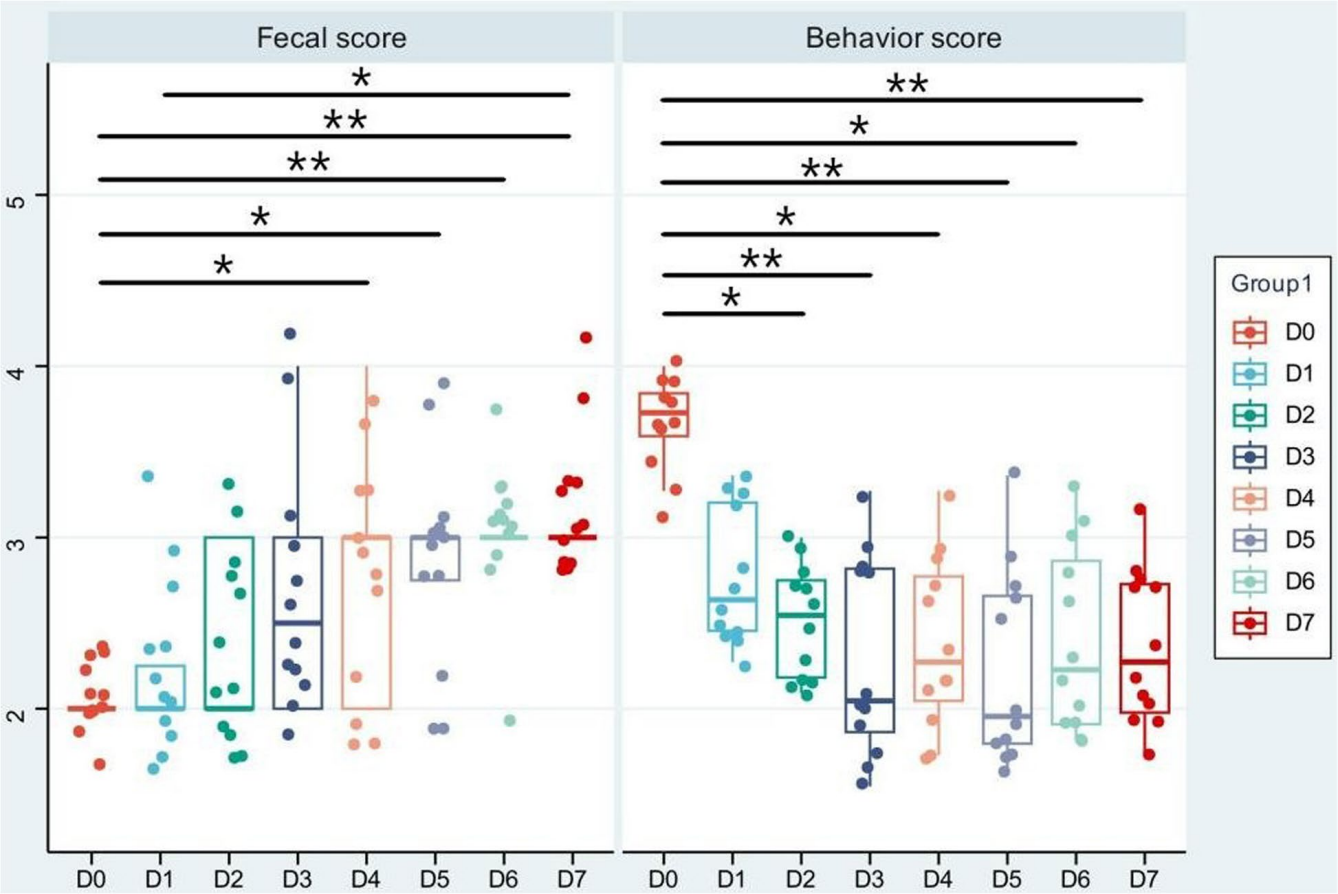
Fecal and behavior scores of dogs (mean ± SD, *n* = 12). Each dot represents an independent biological replicate. Statistical significance was determined using Friedman’s test for overall effect and Wilcoxon signed-rank test for post-hoc pairwise comparisons followed by Bonferroni’s test. ^*^adjusted *P* < 0.05, ^**^adjusted *P* < 0.01

Fecal quality and behavior were monitored daily over the one-week recovery period (D0 to D7) to assess the physiological and behavioral manifestations of transportation stress (Fig. 1). Fecal score exhibited significant variation across days (*P* < 0.01). The score increased markedly from D0 (2.1 ± 0.4) to D1 (3.5 ± 0.3; *P* < 0.01). This improvement continued through D7 (4.2 ± 0.2), with significant pairwise differences between multiple time points including D1 vs. D3, D2 vs. D5, and D3 vs. D7 (*P* < 0.05). Behavior scores showed significant fluctuations (*P* < 0.01). Scores were reduced at D0 (2.5 ± 0.3) and D1 (2.3 ± 0.4). Values increased from D2 (3.1 ± 0.3) to D4 (3.8 ± 0.3; *P* < 0.05), decreased at D5 (3.4 ± 0.3; *P* < 0.05 vs. D4), and stabilized at D7 (3.9 ± 0.2). Significant differences were observed between D1 and D4, and between D5 and D7 (*P* < 0.05).

### Microbial community dynamics

**Fig. 2:**
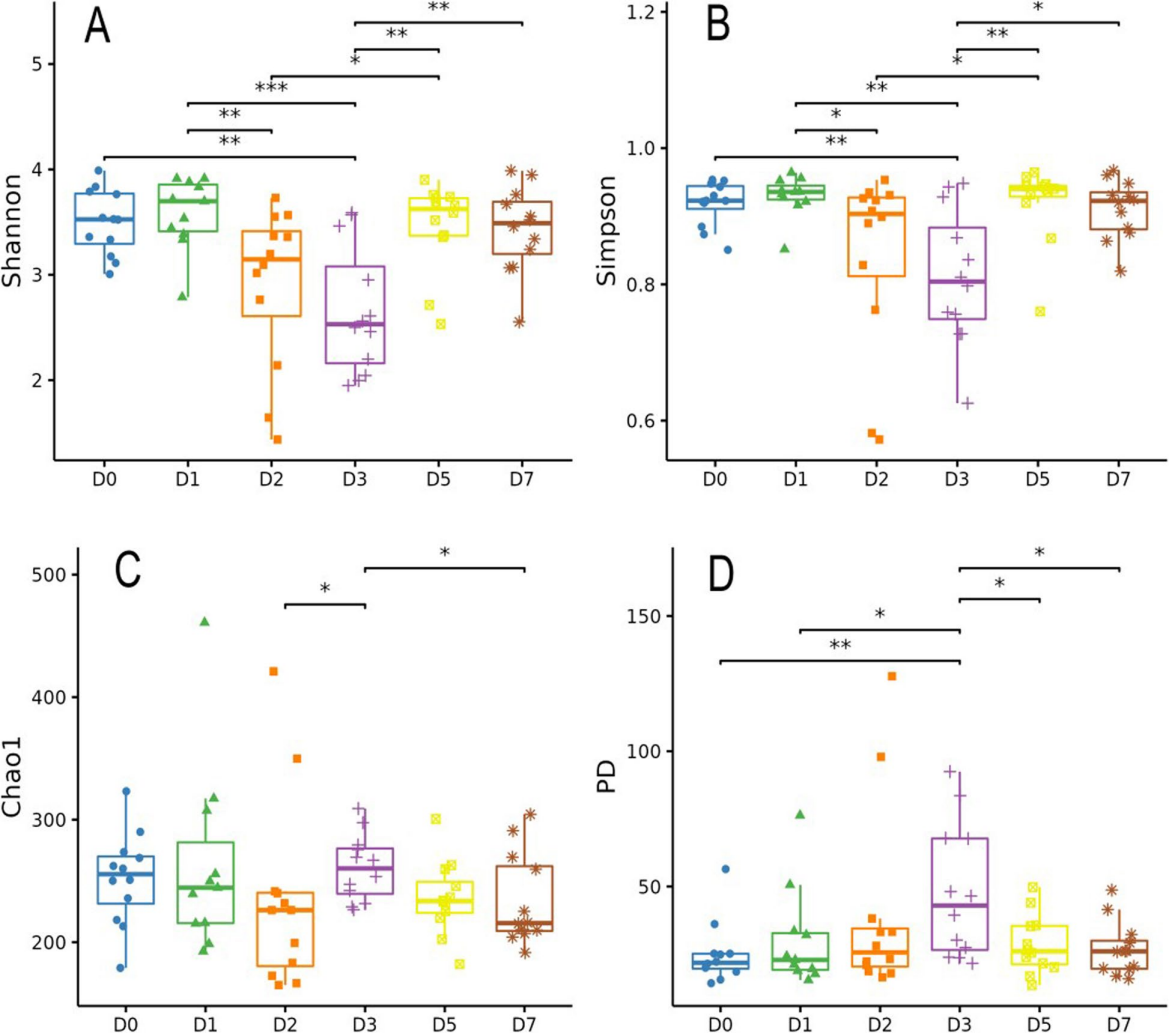
Alpha diversity of the canine fecal microbiota (mean ± SD, *n* = 12). **A** Shannon index. **B** Simpson index. **C** Chao1 index. **D** Phylogenetic diversity (PD) index. Each dot represents an independent biological replicate. Statistical significance was determined using one-way ANOVA followed by FDR correction. ^*^FDR-adjusted *P* < 0.05, ^**^FDR-adjusted *P* < 0.01

Alpha diversity analysis revealed significant shifts in the microbial community structure over the recovery period (Fig. 2). Differences in alpha diversity were observed among the six experimental groups. From D0 to D7, microbial diversity indices (Shannon, Simpson, and Chao1) showed a significant decrease at D2 and D3, followed by a partial recovery at D5 and D7; however, the values did not return to the levels observed at D0 and D1. In contrast, the PD index exhibited an opposite trend, reaching its peak at D3 before returning to levels comparable to D0 and D1.

**Fig. 3:**
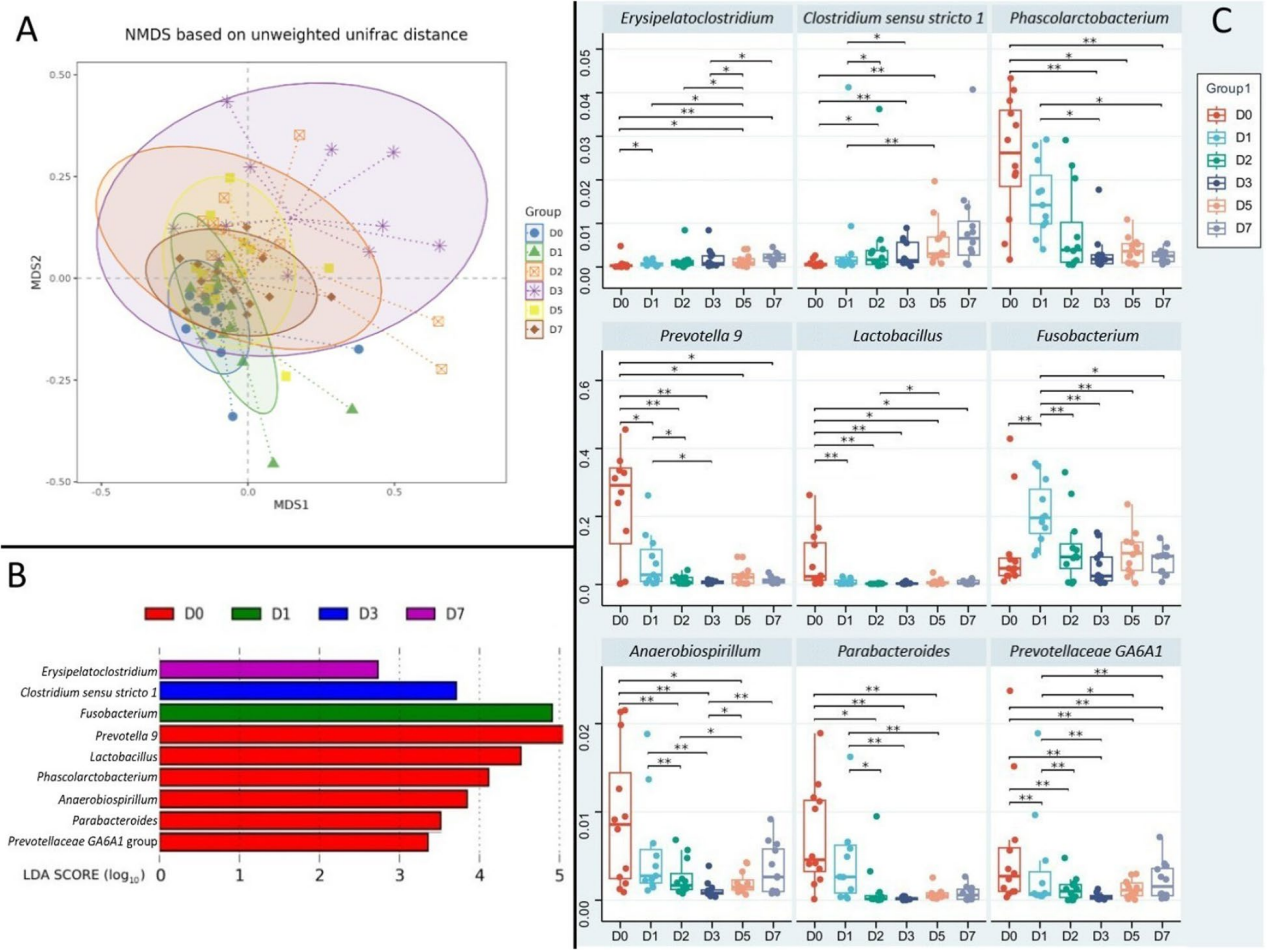
Results of fecal microbiome analysis. **A** Non-Metric Multidimensional Scaling (NMDS) beta diversity index based on unweighted UniFrac distance. **B** Discriminatory taxa at the genus level based on LEfSe analysis; LDA score represents log_10_ transfered LEfSe score of each differential bacteria genus, a significance threshold set at an LDA score > 2.0 and a FDR-adjusted *P* < 0.05. **C** Box-plots of the relative abundance of marker bacteria (mean ± SD, *n* = 12). Each dot represents an independent biological replicate. Statistical significance was determined using Wilcoxon signed-rank test followed by Bonferroni’s test, ^*^adjusted *P* < 0.05, ^**^adjusted *P* < 0.01

Beta diversity analysis further demonstrated significant temporal changes in community composition. Specifically, PERMANOVA indicated that the D0 community structure was significantly different from all other time points (*R*^2^ ranging from 0.242 to 0.302, *P* < 0.01; Additional file 1). NMDS analysis also showed separation across time points (Fig. 3A). Samples from D0 were distinctly separated from those of all other time points, indicating a pronounced difference in microbial community structure immediately post-transport. The greatest separation was observed between D0 and D3. By D7, the microbial community composition shifted closer to D0/D1 but remained significantly different, indicating persistent differences during the first week post transport.

The LEfSe analysis was employed to identify discriminatory taxa at the genus level (Fig. 3B). The initial post-transport (D0) community was characterized by a consortium of genera including *Prevotella 9*, *Lactobacillus*, *Phascolarctobacterium*, *Anaerobiospirillum*, *Parabacteroides*, and *Prevotellaceae GA6A1* group. The analysis identified *Fusobacterium* as the significantly discriminatory taxa at D1, while *Clostridium sensu stricto 1* was significantly enriched at D3. *Erysipelatoclostridium* was significantly enriched at D7. Statistical analysis revealed significant inter-group variations in the relative abundance of characteristic microbial species across the sampling time points (*P* < 0.05, Fig. 3C). *Erysipelatoclostridium* and *Clostridium sensu stricto 1* demonstrated a progressive increase from D0 to D7, while *Phascolarctobacterium* and *Lactobacillus* exhibited a biphasic pattern with an initial decrease followed by partial recovery. *Prevotella 9*, *Anaerobiospirillum*, *Parabacteroides*, and *Prevotellaceae GA6A1* group displayed a U-shaped trajectory with significant reduction and subsequent recovery. *Fusobacterium* abundance peaked transiently at D1 before gradually decreasing.

Functional prediction analysis suggested that the differentially abundant bacterial taxa identified during the recovery period were associated with 14 distinct predicted functional pathways (Additional file 1). These predicted pathways include HCOMODA/2-hydroxy-3-carboxy-muconic semialdehyde decarboxylase; collagen type Vll alpha; xylan 1,4-beta-xylosidase; exosome complex exonuclease DIS3/RRP44; all-*trans*-retinol 13,14-reductase; UDP-D-galactose:(glucosyl)LPS alpha-1,6-D-galactosyltransferase; 5-guanidino-2-oxopentanoate decarboxylase; type IV secretion system protein VirB7; itaconyl-CoA hydratase/mesaconyl-C4 CoA hydratase; undecaprenyl-phosphate 4-deoxy-4-formamido-L-arabinose transferase; Sep-tRNA:Cys-tRNA synthetase; two component system, NtrC family, nitrogen regulation response regulator NtrX; L-arginine dehydrogenase; adenosylcobinamide hydrolase.

### Metabolomic profiling

**Fig. 4:**
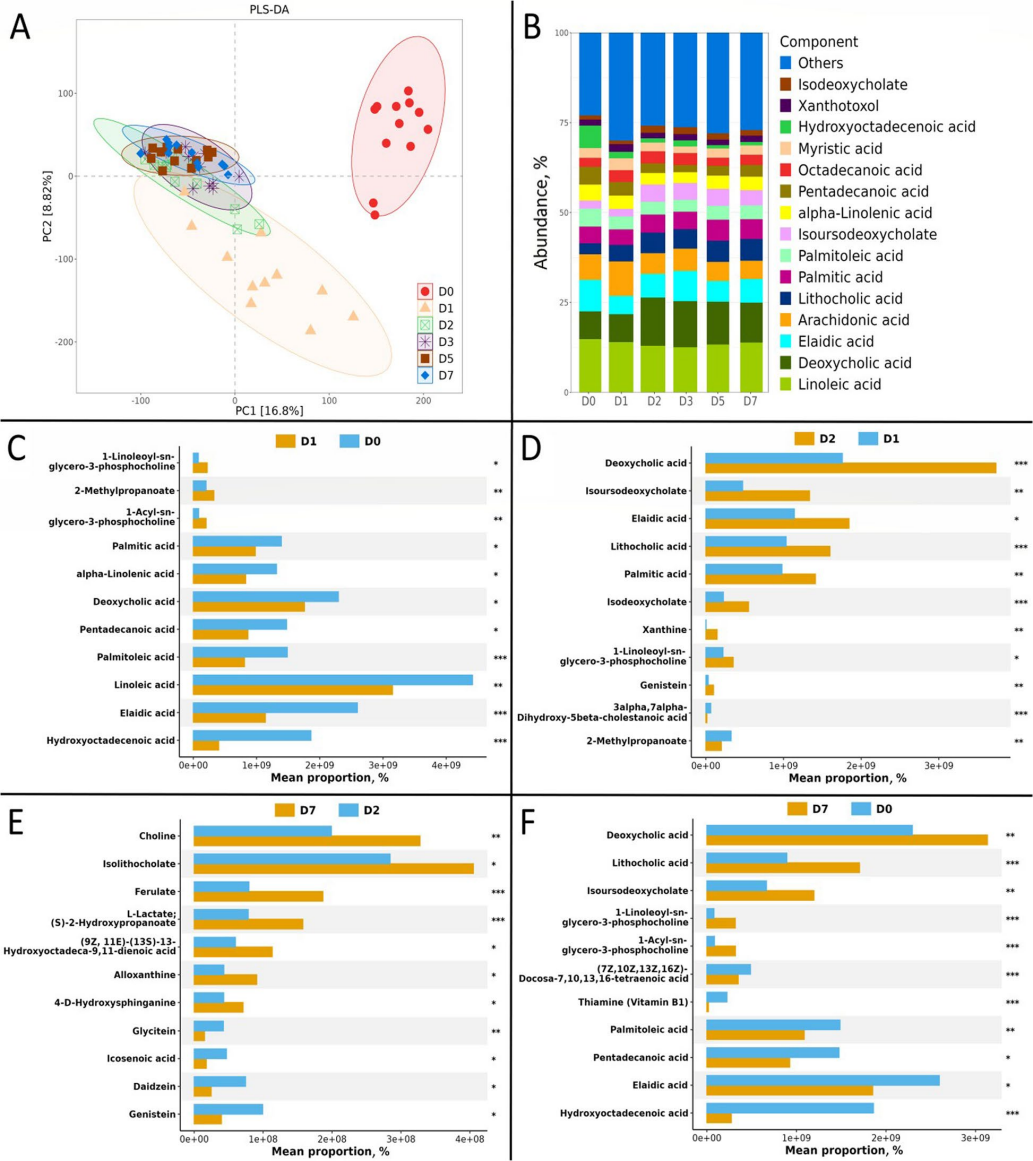
Results of fecal metabolomic profiles. **A** Metabolite distribution based on PLS-DA; the model was cross-validated with an R^2^Y of 0.562, Q^2^ of 0.732, and valid permutation test. **B** Distinct compositional metabolite classes. **C** Significant differences in the proportions of metabolites at D0 vs. D1. **D** Metabolites at D1 vs. D2. **E** Metabolites at D2 vs. D7. **F** Metabolites at D0 vs. D7. Mean proportions represent the relative abundance of the average peak intensity of the metabolite in time points. Each dot represents an independent biological replicate. Statistical significance was determined using Wilcoxon signed-rank test followed by Bonferroni’s test, ^*^adjusted *P* < 0.05, ^**^adjusted *P* < 0.01, ^***^adjusted *P* < 0.001

PLS-DA was used to visualize global metabolomic profiles across time points (R^2^Y = 0.562, Q^2^ = 0.732; Fig. 4A). The D0 group showed a distinct metabolome that was clearly separated in the score plot from subsequent time points (D1–D7). Separation among later time points (D2–D7) appeared reduced compared with the D0-to-later shift.

The stacked bar chart analysis revealed distinct compositional patterns in major metabolite classes across the recovery period (Fig. 4B). Secondary bile acids including deoxycholic acid, isodeoxycholate, and lithocholic acid demonstrated progressive accumulation from D0 to D7, showing particularly prominent increases in relative abundance during the later recovery phase. Medium-chain fatty acids such as myristic acid, pentadecanoic acid, and palmitic acid maintained relatively stable proportions throughout the observation period. Long-chain polyunsaturated fatty acids exhibited variable patterns: while linoleic acid and α-linolenic acid showed fluctuating abundances with a general decreasing trend, arachidonic acid proportions remained comparatively stable. The oxidative stress marker (9Z)-(12R)-hydroxyoctadecenoic acid displayed highest relative abundance during initial time points followed by gradual reduction. Monounsaturated fatty acids including palmitoleic acid and elaidic acid maintained consistent proportions across groups. The "Others" category, representing diverse minor metabolites, demonstrated substantial relative abundance at all time points, suggesting significant compositional complexity in the metabolite profile throughout the recovery process.

The comparative analysis of metabolite proportions revealed distinct temporal patterns across four key recovery intervals (Fig. 4C–F). In the D1 versus D0 comparison, secondary bile acids including deoxycholic acid demonstrated increased proportions during the initial recovery phase, while specific phospholipid compounds such as 1-Linoleoyl-sn-glycero-3-phosphocholine showed decreased relative abundance. (9Z)-(12R)-Hydroxyoctadecenoic acid exhibited substantial elevation at D1 compared to D0 levels (Fig. 4C). The transition from D2 to D1 was characterized by continued alterations in bile acid metabolism, with deoxycholic acid maintaining its progressive increase. Medium-chain fatty acids including pentadecanoic acid and myristic acid displayed proportion modifications, while unsaturated fatty acids such as linoleic acid began to show stabilization trends after initial fluctuations (Fig. 4D). The D7 versus D2 comparison revealed significant metabolic restructuring in the later recovery phase. Phospholipid metabolites including choline demonstrated markedly increased proportions. Secondary bile acids such as lithocholic acid showed substantial accumulation, while essential fatty acids exhibited stabilized proportional patterns (Fig. 4E). The comprehensive D7 versus D0 analysis highlighted the extensive metabolic shifts over the entire recovery period. Deoxycholic acid and lithocholic acid exhibited sustained elevation, indicating ongoing alterations in bile acid metabolism. Phosphocholine derivatives showed attenuated proportions after initial decreases, and (9Z)-(12R)-hydroxyoctadecenoic acid displayed levels that approached those observed at later time points (Fig. 4F).

### Integrative analysis

**Fig. 5:**
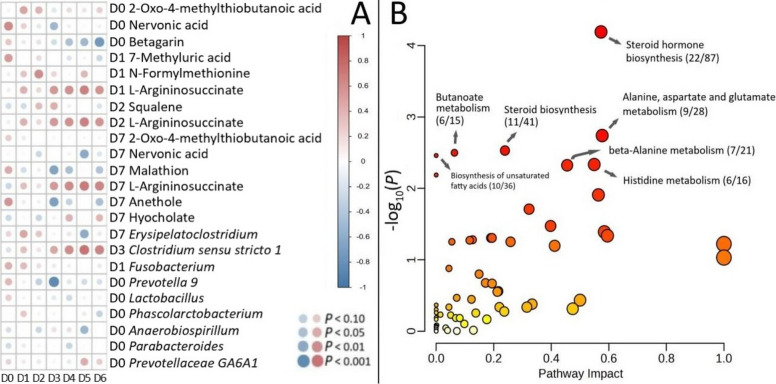
Integrated behavior-microbiome-metabolites correlation analysis. **A** Spearman’s correlation coefficients between behavior score, marker bacteria, and differential metabolites. FDR correction was applied to adjust *P*-values for multiple comparisons. **B** KEGG pathway enrichment analysis of significantly altered metabolites. The *y*-axis and the color scale represent the negative log_10_ of the FDR-adjusted *P*-values. The *x*-axis and the size of each circle represent pathway impact score. The ratio provided in brackets indicates the proportion of significant metabolites relative to the total number of detected compounds mapped to that pathway

Behavioral scores demonstrated significant temporal correlations with specific microbial taxa and metabolite levels throughout the recovery period (Fig. 5A). The scores showed a progressive improvement from D0 to D7, which correlated positively with the increasing abundance of *Erysipelatoclostridium *and* Faecalibacterium*. Conversely, behavioral scores exhibited negative correlations with *Fusobacterium* abundance during the early recovery phase (D1–D2) and with *Clostridium sensu stricto 1* at D3. Metabolite analysis revealed that behavioral improvement aligned with decreased levels of oxidative stress markers (e.g., (9Z)-(12R)-hydroxyoctadecenoic acid) and stabilized essential fatty acids (e.g., nervonic acid, linoleic acid).

Pathway analysis identified seven significantly altered metabolic pathways (*P* < 0.05, Fig. 5B): Steroid hormone biosynthesis, Alanine, aspartate and glutamate metabolism, Steroid biosynthesis, Butanoate metabolism, Biosynthesis of unsaturated fatty acids, Histidine metabolism, and beta-Alanine metabolism.

## Discussion

### Integrated analysis of clinical health parameters and microbial ecosystem dynamics

The comprehensive monitoring of clinical health parameters throughout the 7-day recovery period reveals a complex interplay between physiological manifestations and underlying microbial changes. The fecal score improvement presents an interesting temporal pattern that merits detailed examination in relation to gastrointestinal physiology under stress conditions. The marked improvement from D0 to D1 precedes the significant microbial community changes, indicating that initial gastrointestinal functional recovery may be driven by host physiological adaptations rather than microbial restructuring [25, 26]. However, the sustained improvement through D7, with significant pairwise differences between multiple time points, suggests an increasingly important role of microbial community stabilization in maintaining gastrointestinal health during later recovery stages [25], particularly through the production of gut-health promoting metabolites like short-chain fatty acids and the competitive exclusion of potential pathogens [27].

The behavioral score fluctuations demonstrate the most complex pattern, potentially reflecting the multifaceted nature of stress recovery and its connection to gut-brain axis signaling. The initial reduction at D0–D1 followed by improvement, temporary decline at D5, and final stabilization at D7 suggests that behavioral recovery may involve distinct neurological and physiological processes with different temporal dynamics [28], possibly related to the sequential resolution of different stress response pathways including hypothalamic-pituitary-adrenal axis activation, sympathetic nervous system arousal, and neuro-inflammatory processes [29, 30]. More evidence is needed to provide a deeper explanation for these findings.

### Microbial community succession and functional adaptation

The alpha diversity results reflect fundamental adaptations to the physiological challenges posed by transportation stress. While species richness and evenness decrease temporarily, the increase in phylogenetic diversity suggests that stress exposure may promote the expansion of phylogenetically distinct taxa with specific functional adaptations that enhance community resilience to stress-induced perturbations [31]. The beta diversity results (i.e., PERMANOVA and NMDS), suggested that the microbial community was substantially restructured by D7 compared to D0. The communities at these two time points remained distinct, indicating that the composition observed immediately post-transport had not reverted to the initial state by the end of the study period. These results may reflect a functional adaptation to the post-stress physiological state [26, 32].

The LEfSe analysis identifies specific taxonomic signatures at each recovery phase. The D0 community, characterized by genera including *Prevotella 9* and *Lactobacillus*, represents an arrival state rich in carbohydrate-fermenting specialists that support energy metabolism under stable conditions [33, 34]. The subsequent emergence of *Fusobacterium* at D1 indicates a shift toward mucolytic and inflammatory-associated taxa, consistent with stress-induced gut barrier compromise and the creation of an ecological niche for bacteria capable of utilizing host-derived nutrients released during barrier dysfunction [35]. The dominance of *Clostridium sensu stricto 1* at D3 suggests a transitional phase where both (generally considered) beneficial and (potentially opportunistic) pathogenic taxa co-exist, reflecting the ongoing competition between different ecological strategies during ecosystem recovery [36], while the final predominance of *Erysipelatoclostridium* at D7 indicates establishment of a new stable state focused on secondary metabolite production that may enhance stress tolerance through mechanisms such as butyrate production and bile acid transformation [37].

The functional prediction analysis reveals that these taxonomic changes correspond to significant metabolic shifts with important implications for host physiology during stress recovery. The identified functional pathways encompass core physiological processes ranging from nutrient metabolism to host immune interactions, suggesting that the microbial response to transportation stress involves comprehensive metabolic restructuring that supports host physiological adaptation through multiple mechanisms including energy harvesting, immune modulation, and barrier maintenance [24, 32, 36]. The persistence of pathways related to bacterial pathogenesis throughout the recovery period suggests that even as the community stabilizes, potential pathogenic capacity remains elevated compared to the initial post-transport conditions, possibly representing a trade-off between rapid ecosystem recovery and complete elimination of potential pathogens [16, 38].

### Metabolic shifts and system-level adaptation

The metabolomic profiling provides a system-level perspective on the physiological adaptation process to transportation stress. The PLS-DA results demonstrate that post-arrival metabolic trajectories remain distinct from both microbial community restructuring and clinical parameter normalization, suggesting that metabolic adaptations operate on different temporal scales and may be consistent with both cause and consequence of changes at other organizational levels [39]. The significant separation of D0 from all subsequent time points indicates that metabolic changes persist throughout the recovery period, reflecting the lasting impact of transportation stress on biochemical networks involved in energy production, cellular signaling, and structural maintenance [38, 40], while the attenuated differences from D2 to D7 suggest reduced divergence among later time points, which may be related to the functional needs of the recovering organism [39].

The temporal patterns in specific metabolite classes may suggest sophisticated regulatory mechanisms that coordinate physiological responses to transportation stress. The progressive accumulation of secondary bile acids from D0 to D7 indicates restored microbial metabolic capacity, particularly in the later recovery phases. This likely reflects the importance of bile acid signaling in regulating host metabolism, inflammation, and gut barrier function during stress recovery [41]. This pattern aligns with the establishment of *Erysipelatoclostridium* dominance, as members of this genus have been reported to be involved in bile acid metabolism. Such metabolic feature could reflect co-adaptation between host and microbial metabolism to optimize physiological responses to stress; however, functional inference cannot be confirmed from 16S data alone [41, 42]. The stable proportions of medium-chain fatty acids throughout the observation period suggest preservation of core energy metabolic functions despite community disruptions, possibly representing a metabolic regulatory mechanism that ensures continuous energy supply during the recovery process [43].

The differential metabolite analysis across recovery intervals reveals phase-specific metabolic adaptations. The D1 versus D0 comparison shows potential immediate stress responses characterized by oxidative stress marker elevation (e.g., (9Z)-(12R)-hydroxyoctadecenoic acid) and phospholipid alterations (e.g., 1-linoleoyl-sn-glycero-3-phosphocholine). These marker metabolites have been shown to be involved in redox imbalance and membrane remodeling, potentially reflecting the combined challenges of psychological stress, physical exertion, and environmental changes during transport [44, 45]. The D2 versus D1 transition demonstrates metabolic balance efforts through bile acid metabolism adjustments, this may be due to a shift toward long-term adaptive strategies that support digestive function, metabolic regulation, and inflammatory control as the immediate stress response subsides [41, 42]. The D7 versus D2 comparison reveals extensive metabolic shifts, while the D7 versus D0 analysis confirms distinct and sustained differences from the initial state. These persistent patterns may reflect an altered metabolic profile that could influence the host's response to subsequent stressors [46].

### Integrated cross-domain interactions and behavioral correlations

The correlation analysis between behavioral scores and microbial/metabolite dynamics suggests possible involvement of the gut-brain axis in stress recovery. The positive correlation between behavioral improvement and *Erysipelatoclostridium*/*Faecalibacterium* abundance could suggest that specific microbial taxa may directly or indirectly influence neurological recovery processes through mechanisms such as butyrate production [37, 38, 47], thereby contributing to the restoration of normal behavior patterns following stress. Conversely, the negative correlations with *Fusobacterium* and *Clostridium sensu stricto 1* may reflect that certain microbial patterns may hinder the recovery of behavioral states, possibly through the promotion of systemic inflammation or disruption of gut barrier function [35, 36]. Nevertheless, these hypotheses require further experimental investigation and rigorous causality assessment to establish mechanistic relationships.

The metabolomic analysis reveals complex time-dependent associations that extend beyond simple linear relationships and suggest the existence of coordinated response modules that integrate microbial and host metabolism during stress recovery. The coordinated dynamics of 2-oxo-4-methylthiobutanoic acid and nervonic acid with *Prevotella 9* and *Lactobacillus* abundance suggests the existence of specific microbial-metabolite modules that function as integrated units during recovery, possibly representing synergistic relationships that support neurological function through the provision of neuroprotective fatty acids and keto acids that serve as alternative energy sources for the brain during the metabolic challenges of recovery [33, 34, 48, 49]. The persistent elevation of L-argininosuccinate aligned with *Fusobacterium* and *Clostridium sensu stricto 1* dynamics indicates sustained alterations in nitrogen metabolism that may influence multiple physiological systems including nitric oxide signaling, urea cycle function, and polyamine metabolism that collectively regulate vascular function, immune responses, and cellular proliferation during tissue repair and recovery processes [35, 36, 50].

The pathway analysis identifies seven core metabolic pathways that undergo significant alterations throughout the recovery process, highlighting the key biochemical systems that are prioritized during physiological adaptation to transportation stress. The prominence of steroid hormone biosynthesis and steroid biosynthesis pathways suggests ongoing changes in endocrine function that may regulate stress responsiveness, immune function, and metabolic programming long after the cessation of the initial stressor, potentially representing a mechanism for enhanced preparedness for future challenges [51, 52]. The alterations in amino acid metabolism pathways (alanine, aspartate, glutamate, histidine, and beta-alanine) indicate fundamental alterations in nitrogen metabolism that supports the biosynthetic demands of tissue repair, immune cell proliferation, and neurotransmitter synthesis during recovery [53], while the inclusion of butanoate metabolism reflects the importance of short-chain fatty acid production in recovery processes, particularly through its roles in providing colonocyte energy, regulating immune function, and maintaining gut barrier integrity [54].

### Limitations and future research directions

While this study provides comprehensive insights into the recovery process from transportation stress, several limitations should be acknowledged in interpreting the physiological significance of the findings. The absence of pre-transport baseline measurements limits our ability to distinguish between stress-induced changes and normal temporal variations, making it difficult to determine whether the observed D0 state represents the true baseline or already reflects early stress adaptation. The focus on fecal microbiota may not fully capture changes in mucosal-associated communities that might have more direct host interactions and potentially greater physiological significance in stress responses, particularly regarding immune regulation and gut barrier function. Another limitation is that repeated on-site behavior/clinical scoring, while randomized, may not have been fully blinded to timepoint, introducing potential for expectation bias. Moreover, it is important to acknowledge that transportation is a complex event accompanied by several potential confounders, including environmental novelty, climate variations, and changes in handling and housing. These co-occurring factors are likely significant drivers of the observed microbiome and metabolome changes, and their individual contributions cannot be disentangled in the present study design.

Notably, quantifying “stress” in dogs is inherently challenging, as no single metric provides a definitive readout. Behavioral and fecal scoring are subjective and susceptible to observer/context effects. Even objective measures like cortisol can be difficult to interpret due to timing, handling effects, and inter-individual variability. Physiologic markers such as heart rate, body temperature, and pupillary dilation can add context but still do not constitute a standardized, universally accepted protocol for “stress” assessment in dogs. Further studies are needed to better measure stress in dogs.

Future research should incorporate longitudinal sampling before, during, and after transportation to establish true baseline values and capture dynamic changes during stress exposure itself, providing a more complete picture of the physiological adaptation process. Enhanced standardisation of interfering factors that co-occur with transport and arrival (e.g., environmental and climate changes) is urgently required in future experimental designs. Additional omics approaches, including transcriptomics and proteomics, would provide deeper insights into the functional state of both host and microbiota, revealing how genetic potential translates into actual physiological activity during stress recovery [55]. Intervention studies targeting specific microbial taxa or metabolic pathways could help establish causal relationships and identify potential therapeutic targets for enhancing stress resilience and promoting recovery in transported animals [2, 8].

The observed coordination of recovery across clinical, microbial, and metabolic domains may suggest the involvement of system-level regulatory mechanisms. Future controlled studies are needed to test whether interconnected neural, endocrine, and immune signaling pathways, potentially involving the hypothalamic-pituitary-adrenal axis, sympathetic nervous system, and circadian rhythms, orchestrate this multi-system adaptation to stress.

## Conclusion

In summary, this integrated analysis characterizes a recovery trajectory involving complex changes across clinical, microbial, and metabolic domains during the first week post-transport of dogs. The establishment of sustained multi-system differences raises the hypothesis that such experiences could influence longer-term physiological regulation. The specific microbial taxa and metabolic pathways identified, such as those involving *Erysipelatoclostridium*, secondary bile acid metabolism, and cholinergic signaling, represent candidate targets for future interventions aimed at enhancing recovery and resilience in transported dogs.

## Supplementary Information

Additional file 1. The summary of unprocessed clinincal and behavior data and marker bacteria and metabolites.

Additional file 2. Raw data of the microbiome taxa.

Additional file 3. Raw data of the metabolomic compounds.

## Data Availability

The data supporting the findings of this study are included within the article. Further inquiries can be directed to the corresponding author.

## Abbreviations

FDR: False discovery rate
FEDIAF: The European Pet Food Industry Federation
GI: Gastrointestinal
HMDB: Human Metabolome Database
IAABC: International Association of Animal Behaviour Consultants
KEGG: Kyoto Encyclopedia of Genes and Genomes
LEfSe: Linear discriminant analysis effect size
MS: Mass spectrometry
NMDS: Non-metric multidimensional scaling
PD: Phylogenetic diversity
PERMANOVA: Permutational multivariate analysis of variance
PLS-DA: Partial least squares discriminant analysis
SCFA: Short-chain fatty acid
SD: Standard deviation
UPLC: Ultra-high-performance liquid chromatography
