# Supplementary figures and images for: Post-transport recovery trajectory of the canine gut microbiome and metabolome
Source: J Anim Sci Biotechnol. 2026 Apr 12;17:65. doi: 10.1186/s40104-026-01385-z (PMC13070285; doi:10.1186/s40104-026-01385-z)

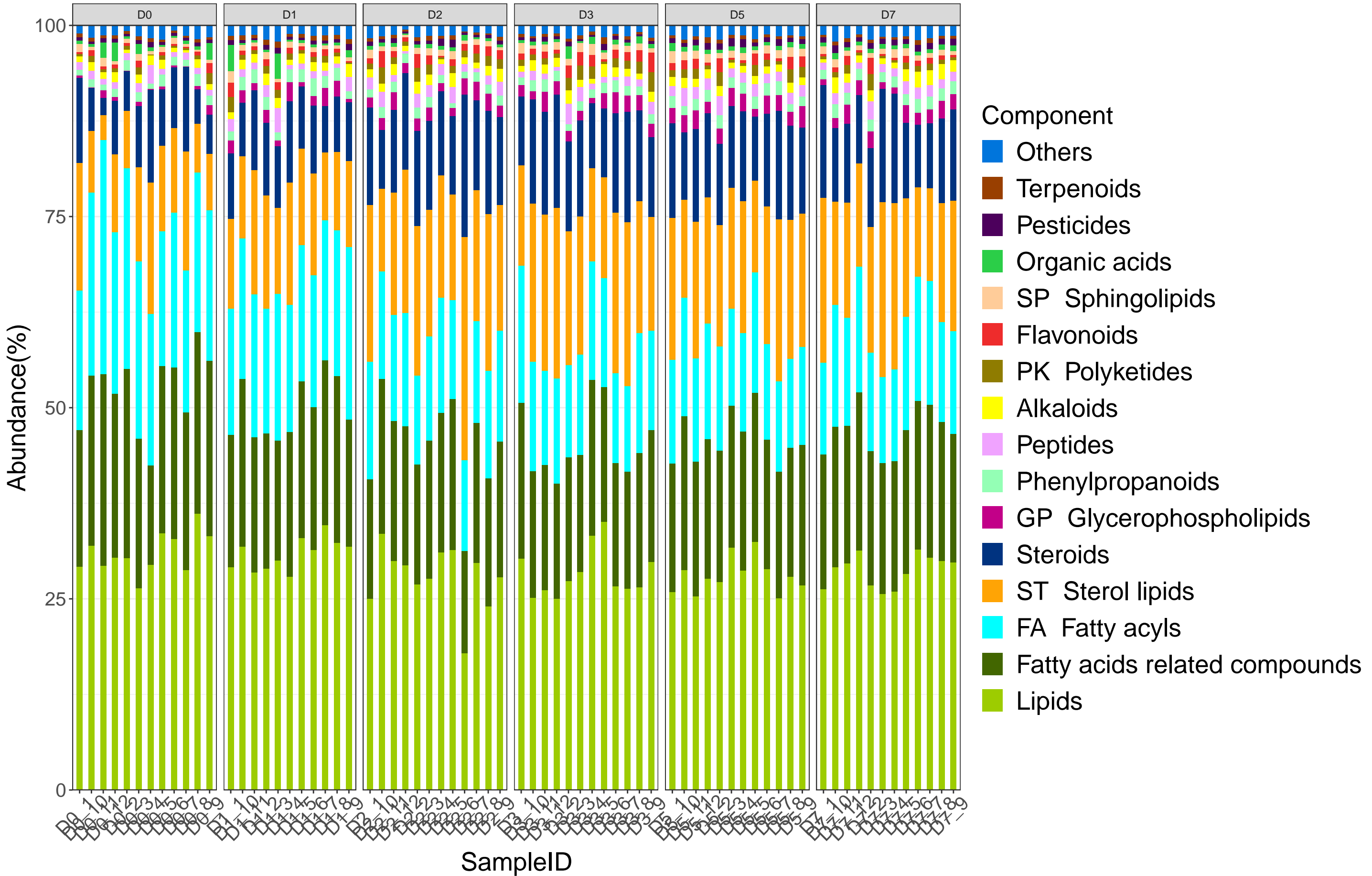

Supplement: Supplementary file 3 — Additional file 3. Raw data of the metabolomic compounds. [file 40104_2026_1385_MOESM3_ESM.zip › mix/KEGG_compound_summary/Barplot/compound_summary_level1_barplot.pdf]

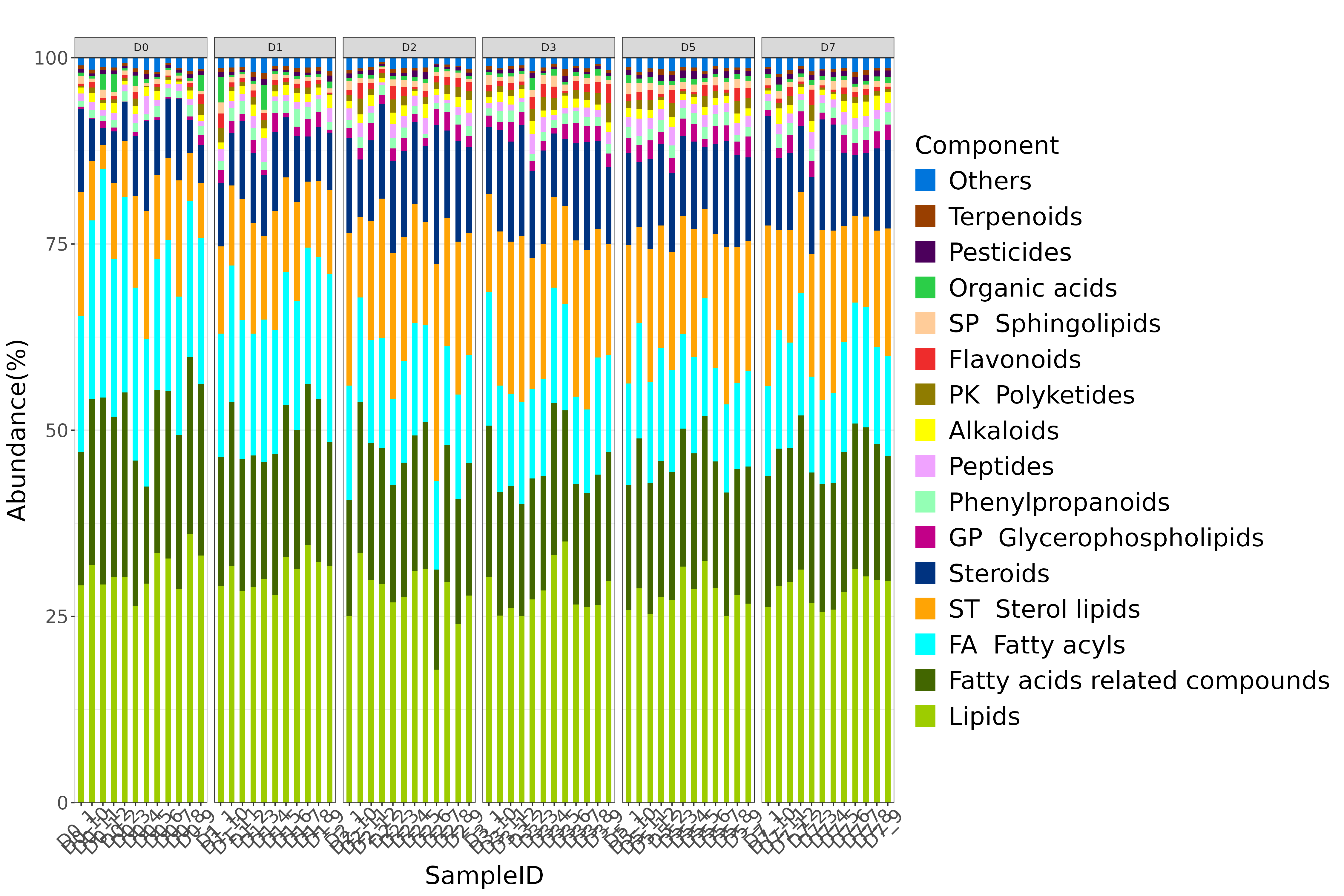

Supplement: Supplementary file 3 — Additional file 3. Raw data of the metabolomic compounds. [file 40104_2026_1385_MOESM3_ESM.zip › mix/KEGG_compound_summary/Barplot/compound_summary_level1_barplot.png]

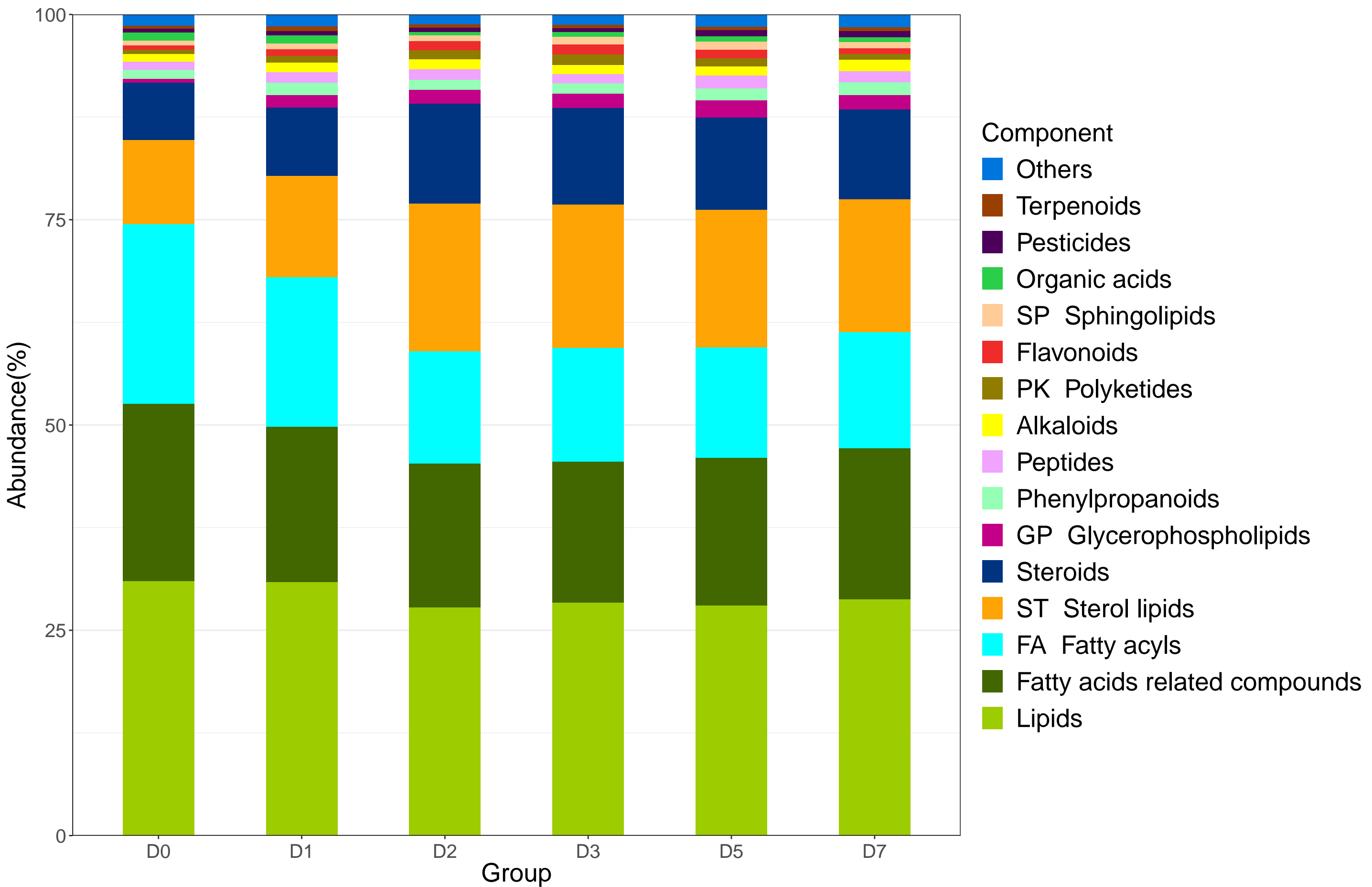

Supplement: Supplementary file 3 — Additional file 3. Raw data of the metabolomic compounds. [file 40104_2026_1385_MOESM3_ESM.zip › mix/KEGG_compound_summary/Barplot/compound_summary_level1_Group_barplot.pdf]

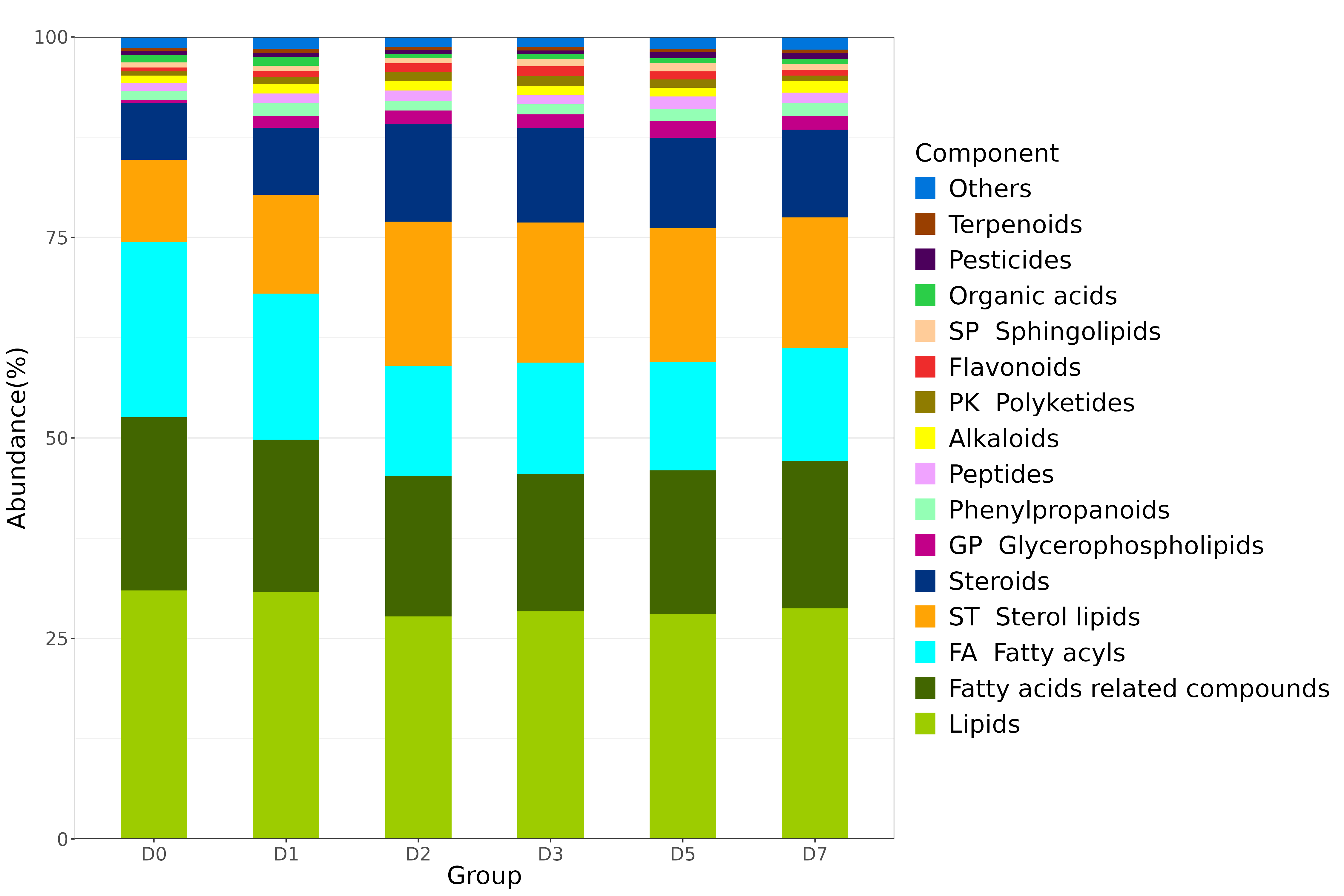

Supplement: Supplementary file 3 — Additional file 3. Raw data of the metabolomic compounds. [file 40104_2026_1385_MOESM3_ESM.zip › mix/KEGG_compound_summary/Barplot/compound_summary_level1_Group_barplot.png]

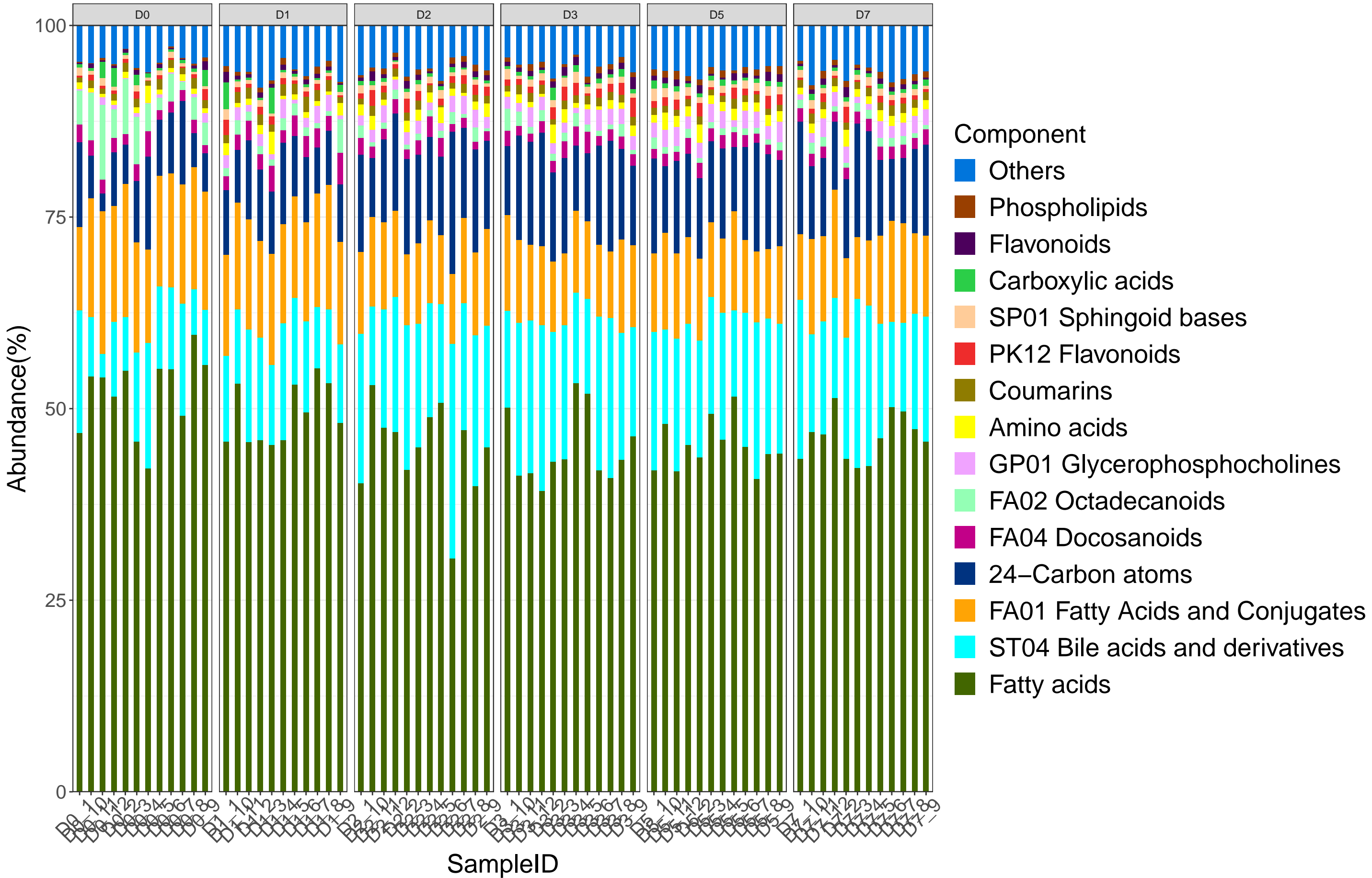

Supplement: Supplementary file 3 — Additional file 3. Raw data of the metabolomic compounds. [file 40104_2026_1385_MOESM3_ESM.zip › mix/KEGG_compound_summary/Barplot/compound_summary_level2_barplot.pdf]

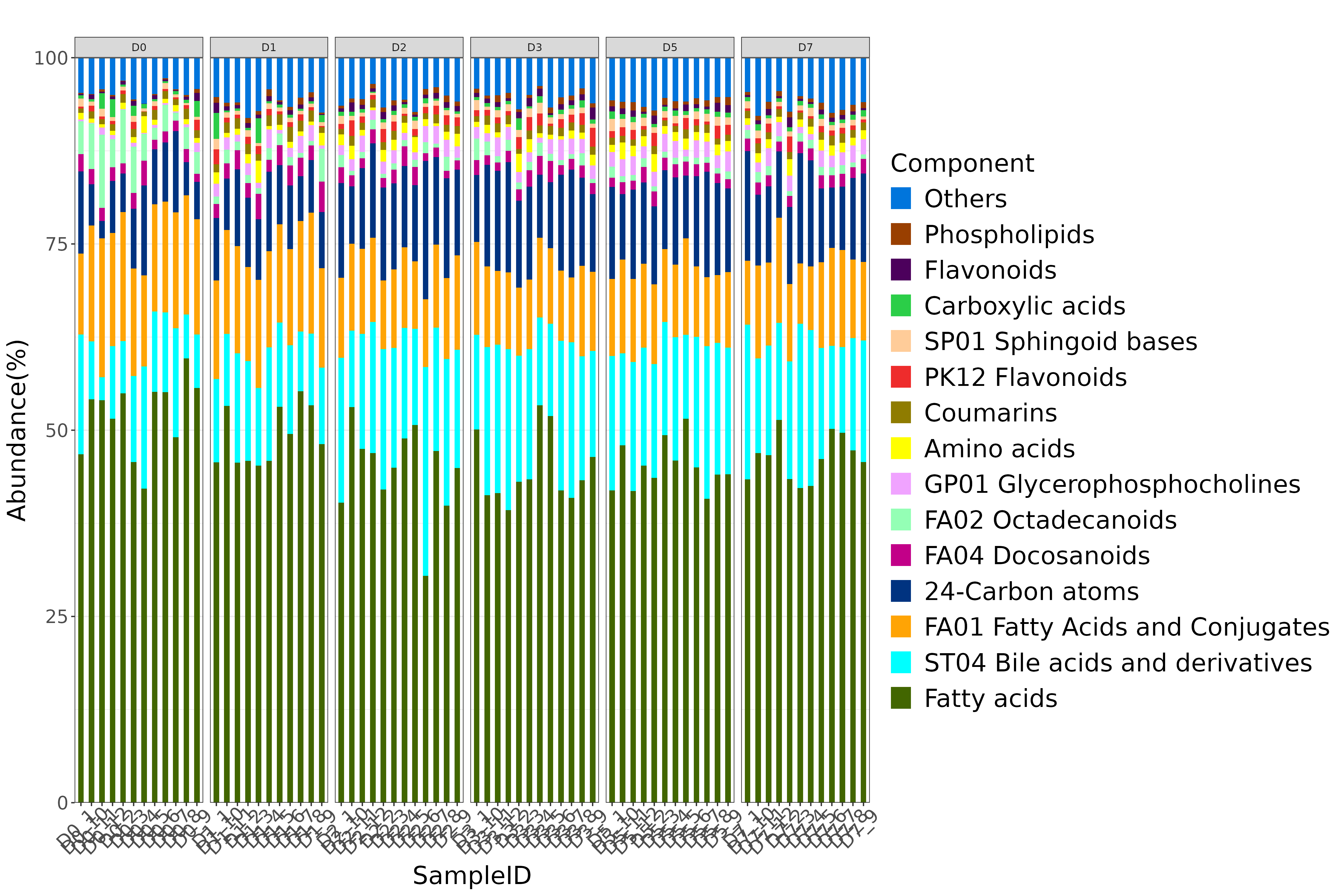

Supplement: Supplementary file 3 — Additional file 3. Raw data of the metabolomic compounds. [file 40104_2026_1385_MOESM3_ESM.zip › mix/KEGG_compound_summary/Barplot/compound_summary_level2_barplot.png]

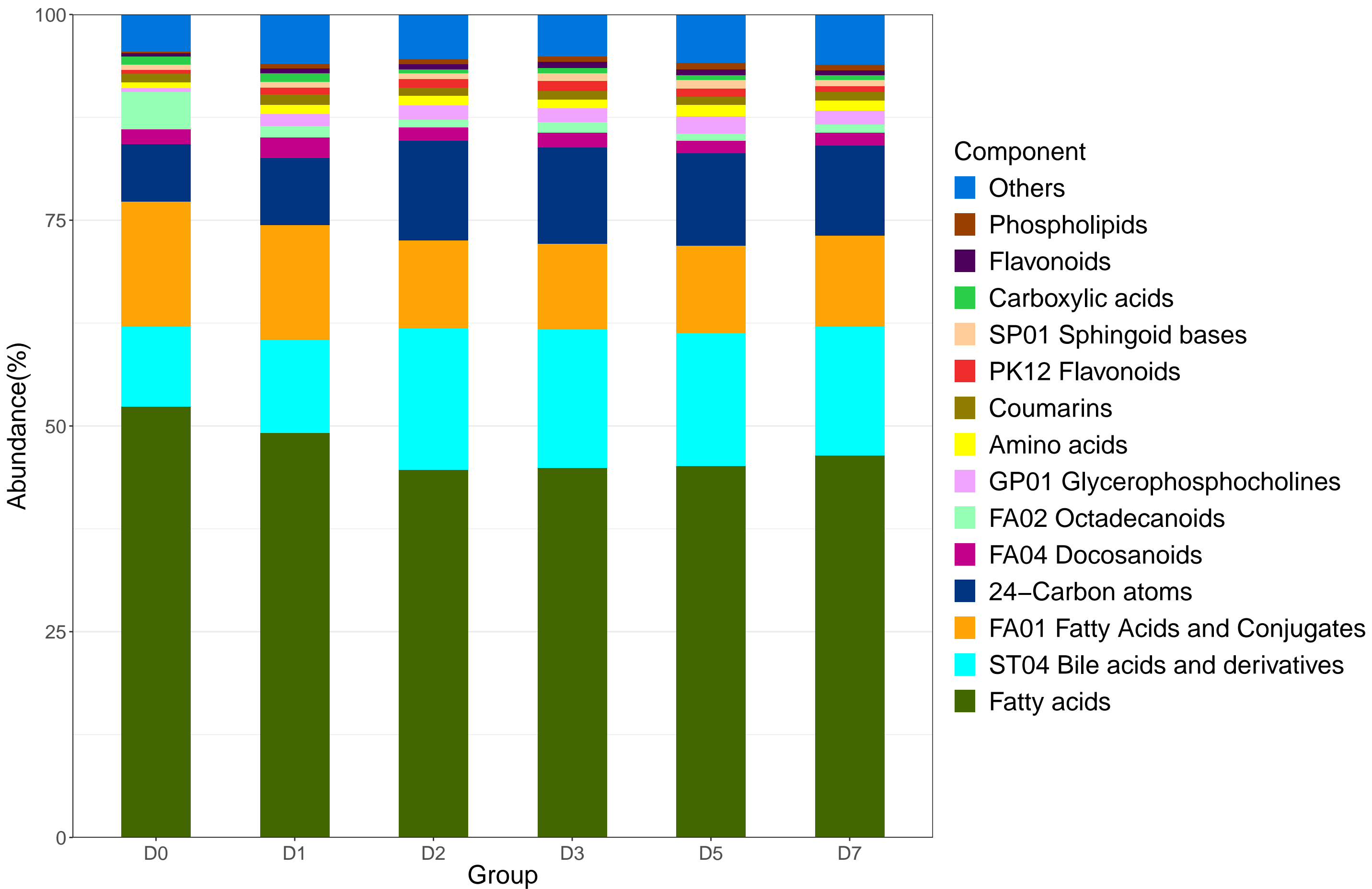

Supplement: Supplementary file 3 — Additional file 3. Raw data of the metabolomic compounds. [file 40104_2026_1385_MOESM3_ESM.zip › mix/KEGG_compound_summary/Barplot/compound_summary_level2_Group_barplot.pdf]

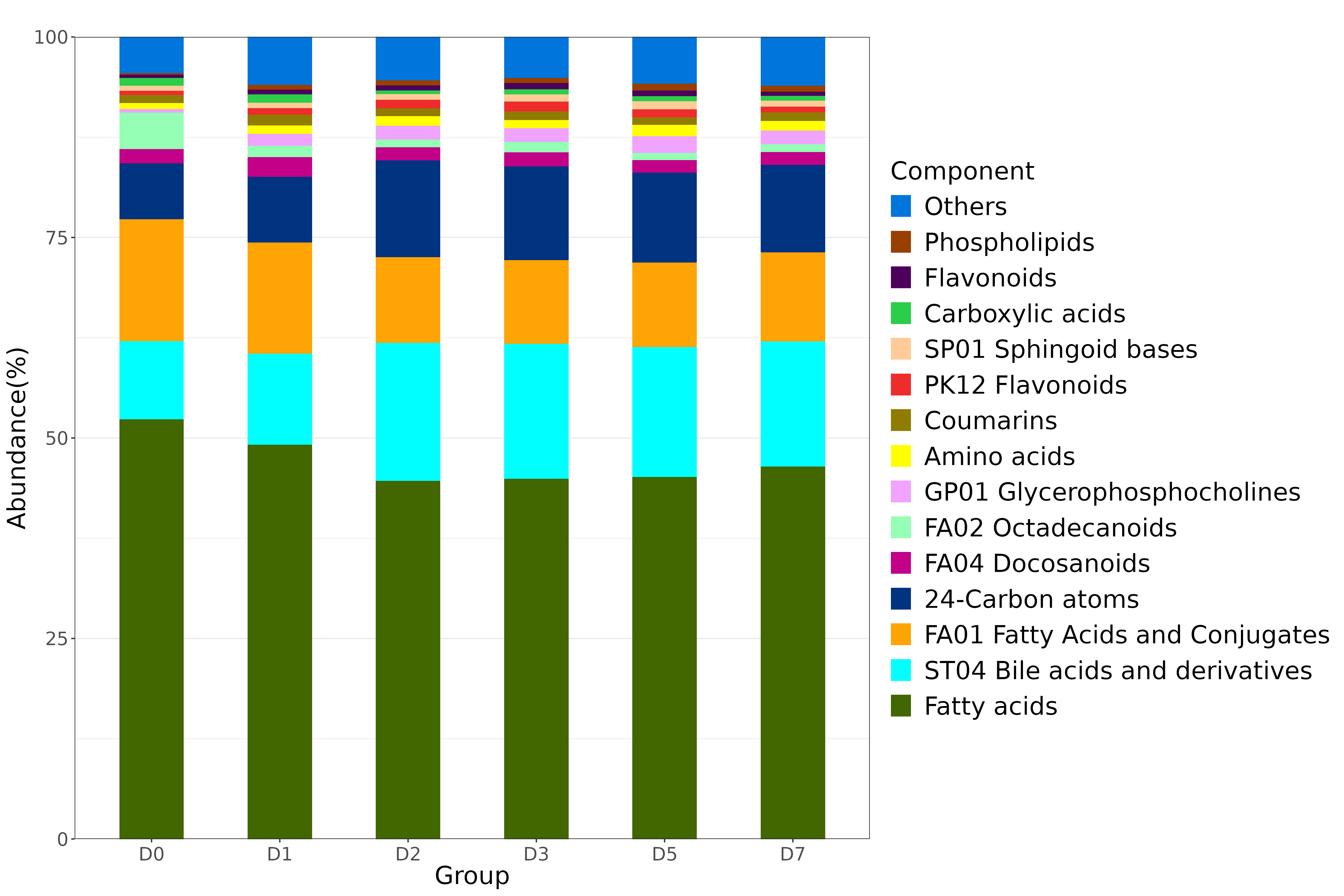

Supplement: Supplementary file 3 — Additional file 3. Raw data of the metabolomic compounds. [file 40104_2026_1385_MOESM3_ESM.zip › mix/KEGG_compound_summary/Barplot/compound_summary_level2_Group_barplot.png]

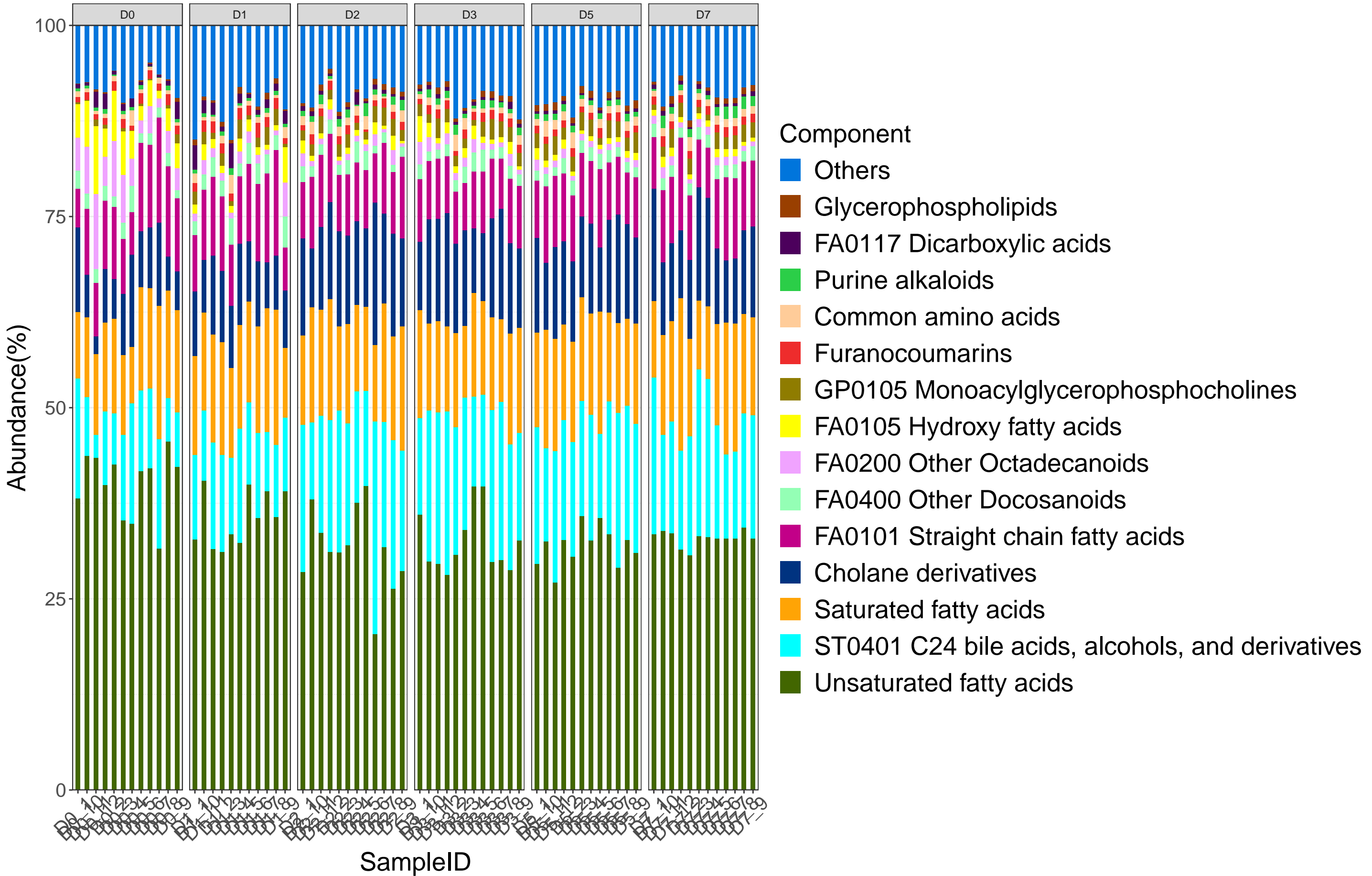

Supplement: Supplementary file 3 — Additional file 3. Raw data of the metabolomic compounds. [file 40104_2026_1385_MOESM3_ESM.zip › mix/KEGG_compound_summary/Barplot/compound_summary_level3_barplot.pdf]

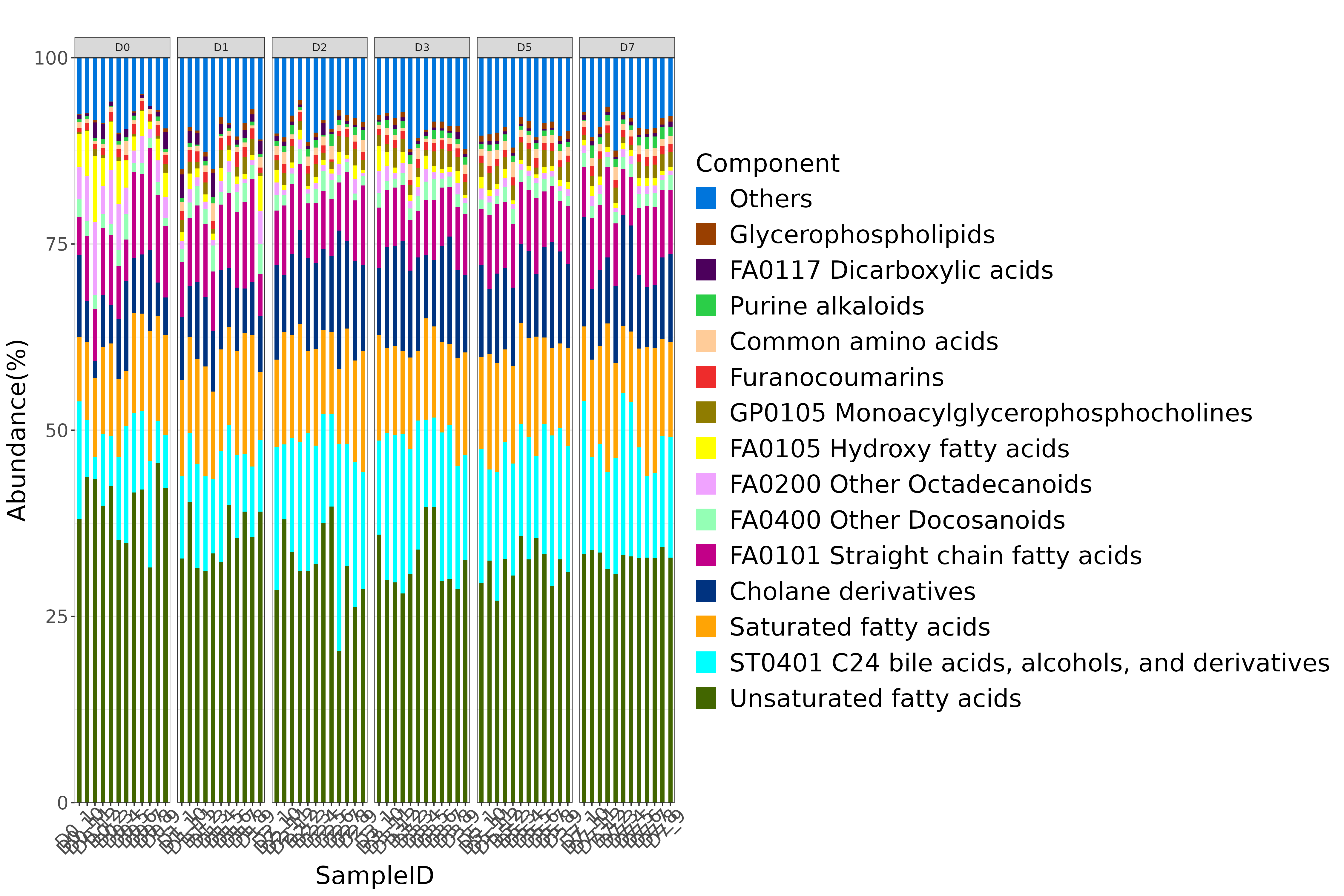

Supplement: Supplementary file 3 — Additional file 3. Raw data of the metabolomic compounds. [file 40104_2026_1385_MOESM3_ESM.zip › mix/KEGG_compound_summary/Barplot/compound_summary_level3_barplot.png]

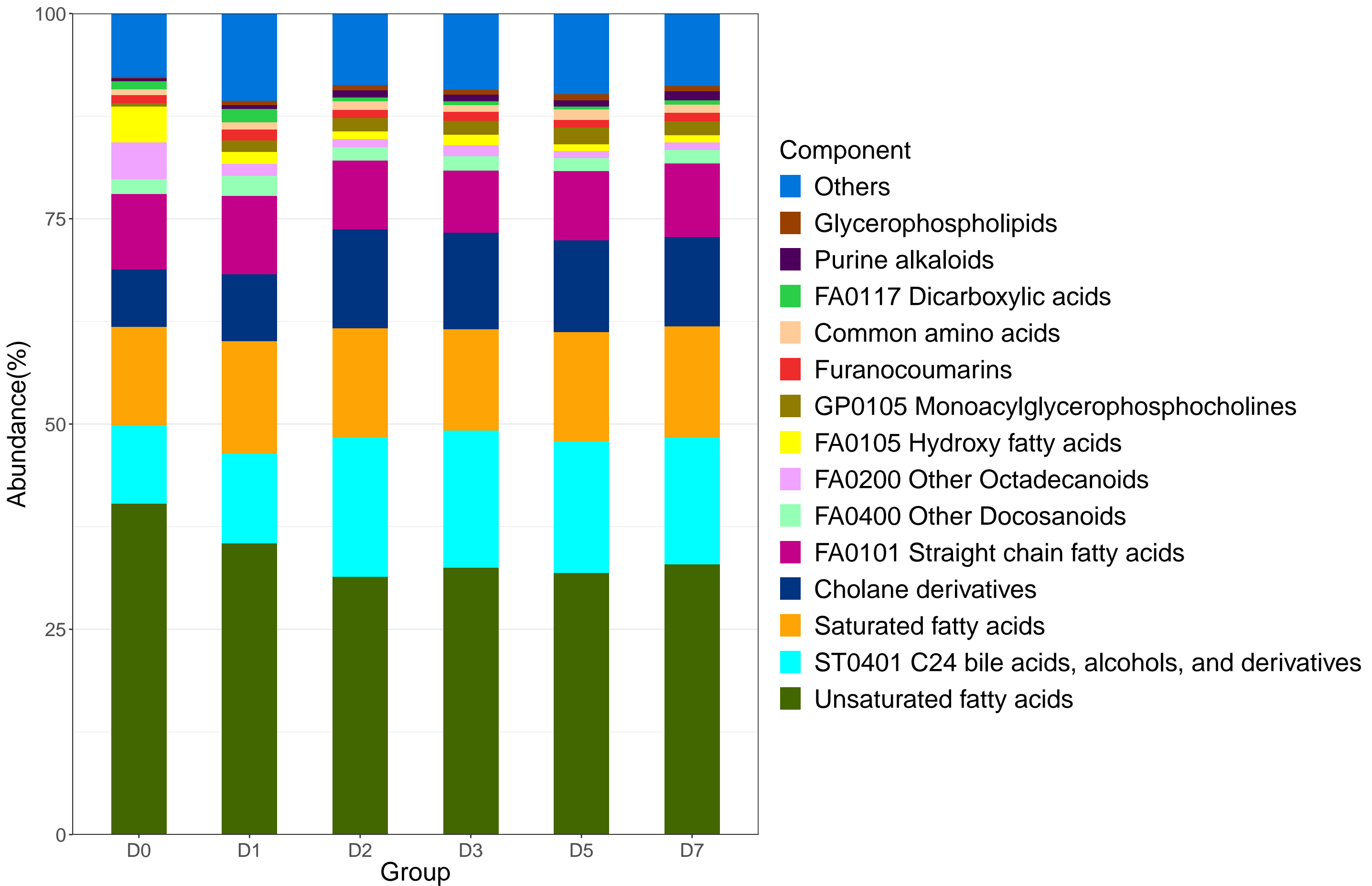

Supplement: Supplementary file 3 — Additional file 3. Raw data of the metabolomic compounds. [file 40104_2026_1385_MOESM3_ESM.zip › mix/KEGG_compound_summary/Barplot/compound_summary_level3_Group_barplot.pdf]

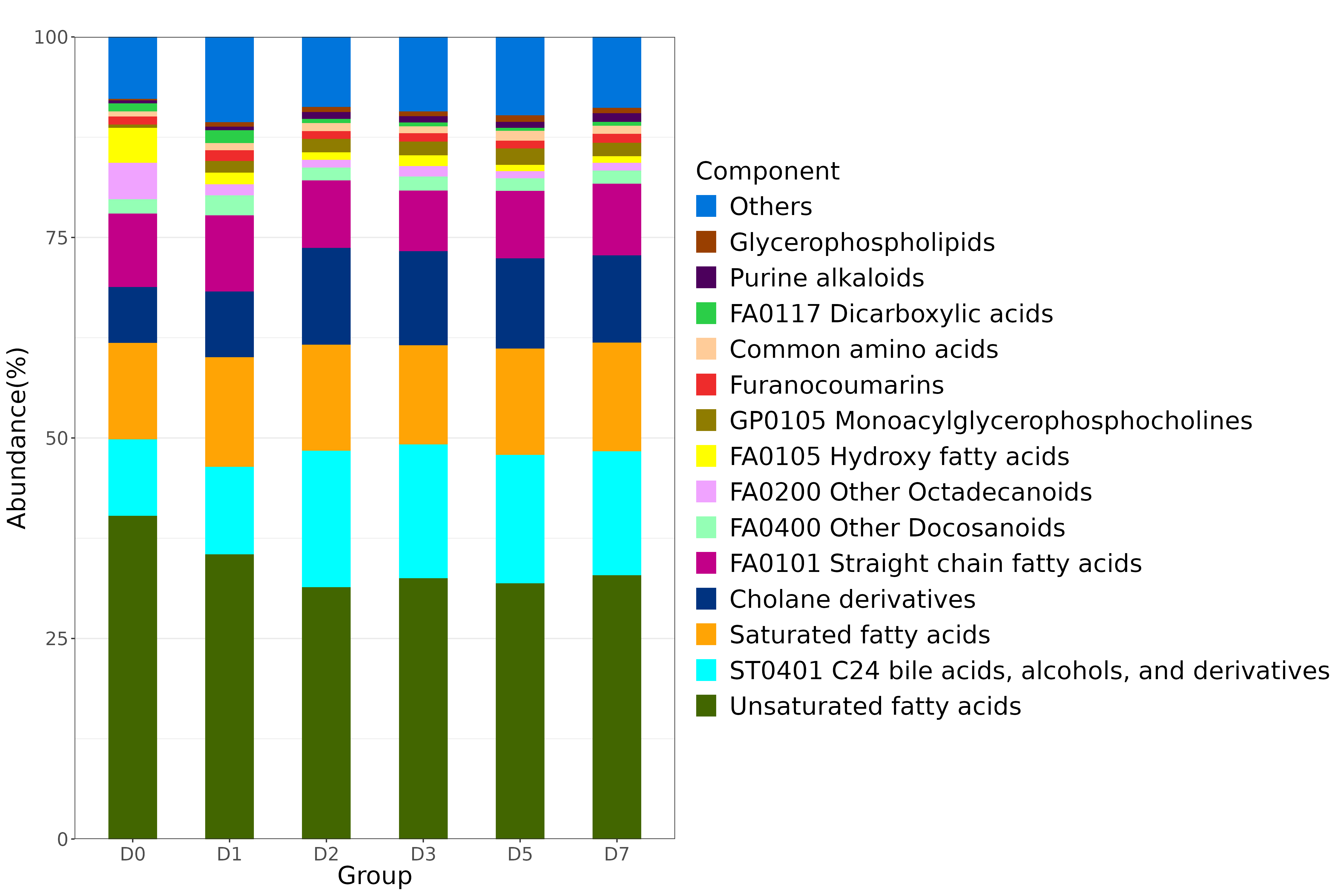

Supplement: Supplementary file 3 — Additional file 3. Raw data of the metabolomic compounds. [file 40104_2026_1385_MOESM3_ESM.zip › mix/KEGG_compound_summary/Barplot/compound_summary_level3_Group_barplot.png]

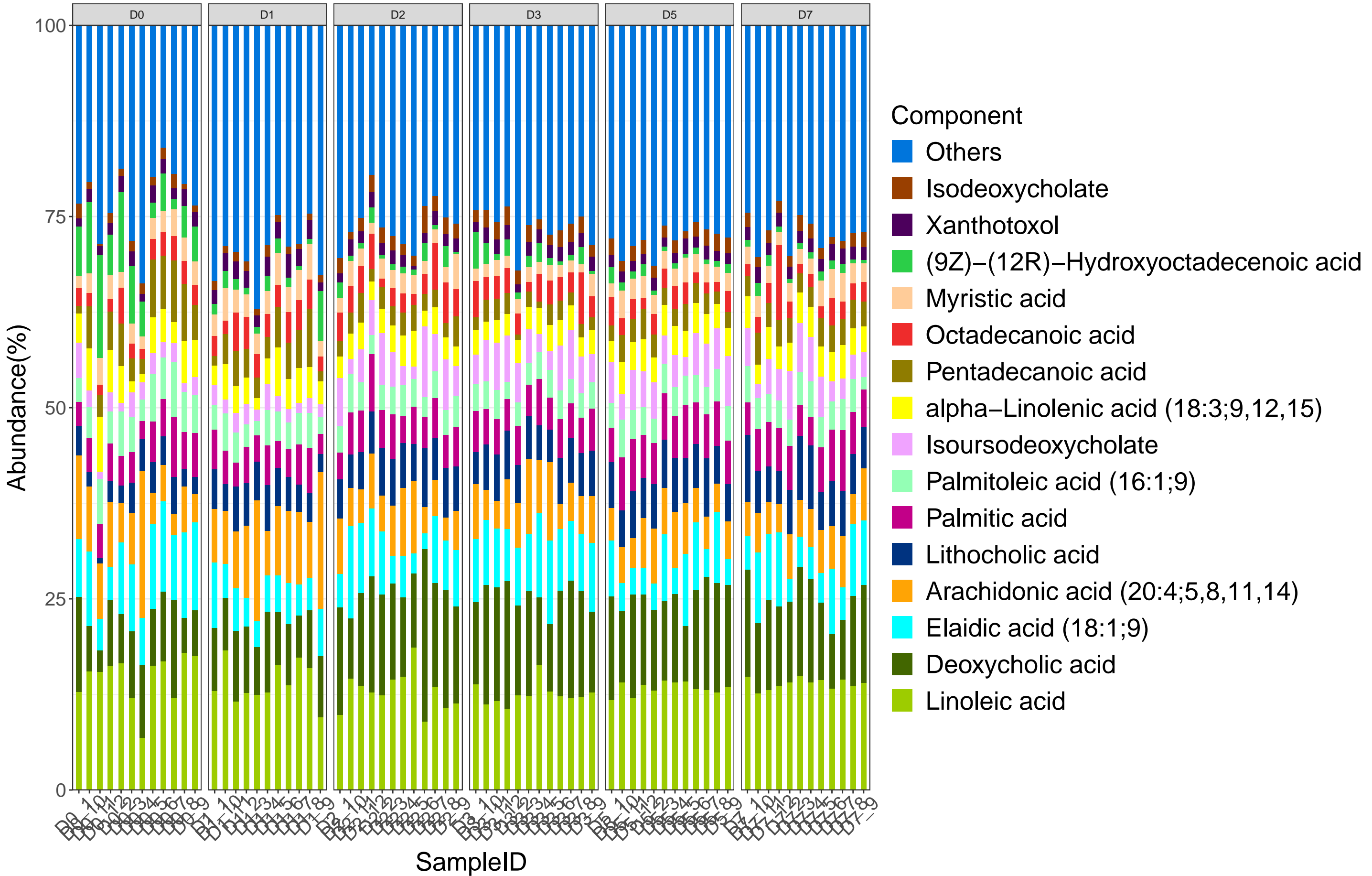

Supplement: Supplementary file 3 — Additional file 3. Raw data of the metabolomic compounds. [file 40104_2026_1385_MOESM3_ESM.zip › mix/KEGG_compound_summary/Barplot/compound_summary_level4_barplot.pdf]

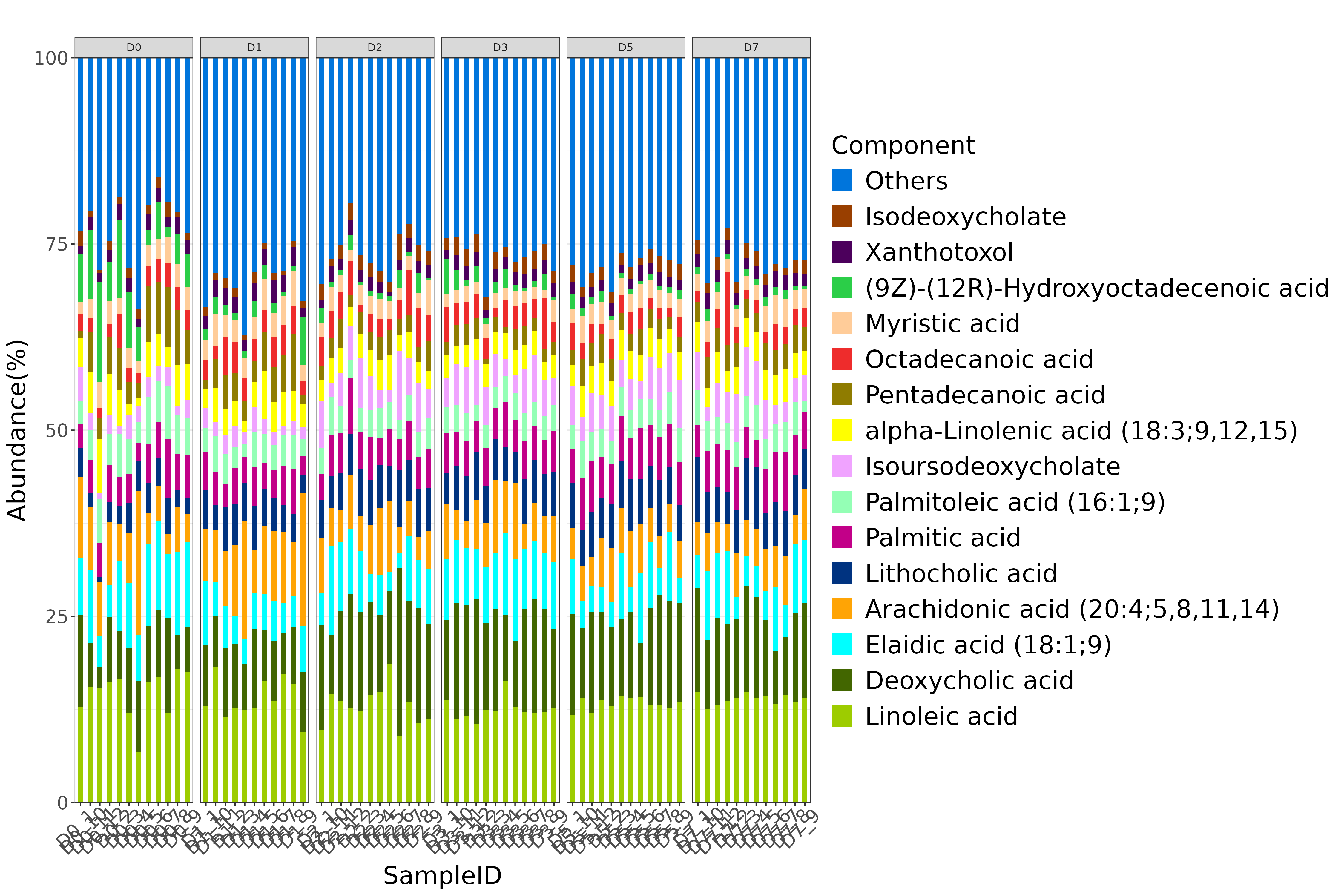

Supplement: Supplementary file 3 — Additional file 3. Raw data of the metabolomic compounds. [file 40104_2026_1385_MOESM3_ESM.zip › mix/KEGG_compound_summary/Barplot/compound_summary_level4_barplot.png]

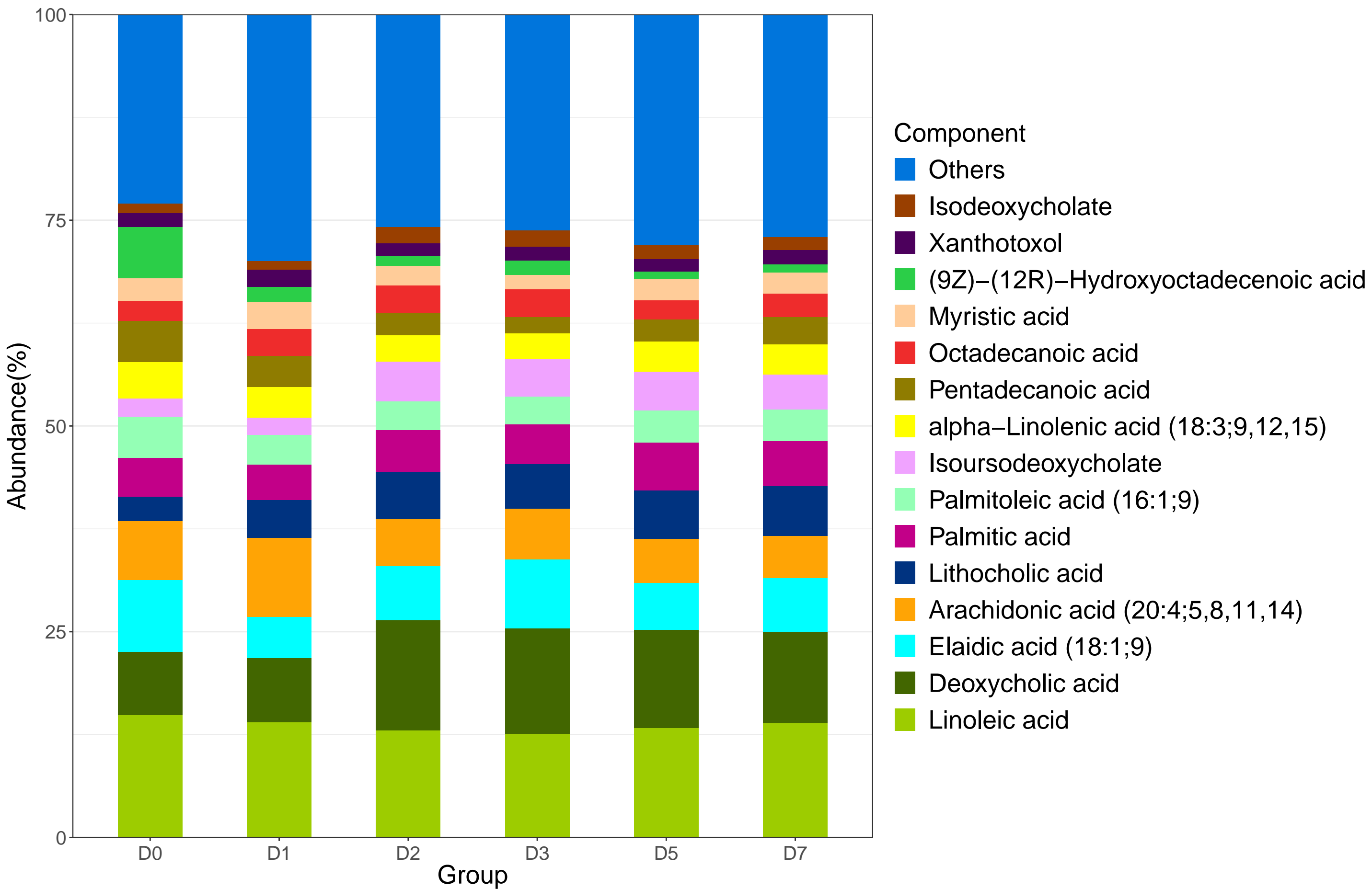

Supplement: Supplementary file 3 — Additional file 3. Raw data of the metabolomic compounds. [file 40104_2026_1385_MOESM3_ESM.zip › mix/KEGG_compound_summary/Barplot/compound_summary_level4_Group_barplot.pdf]

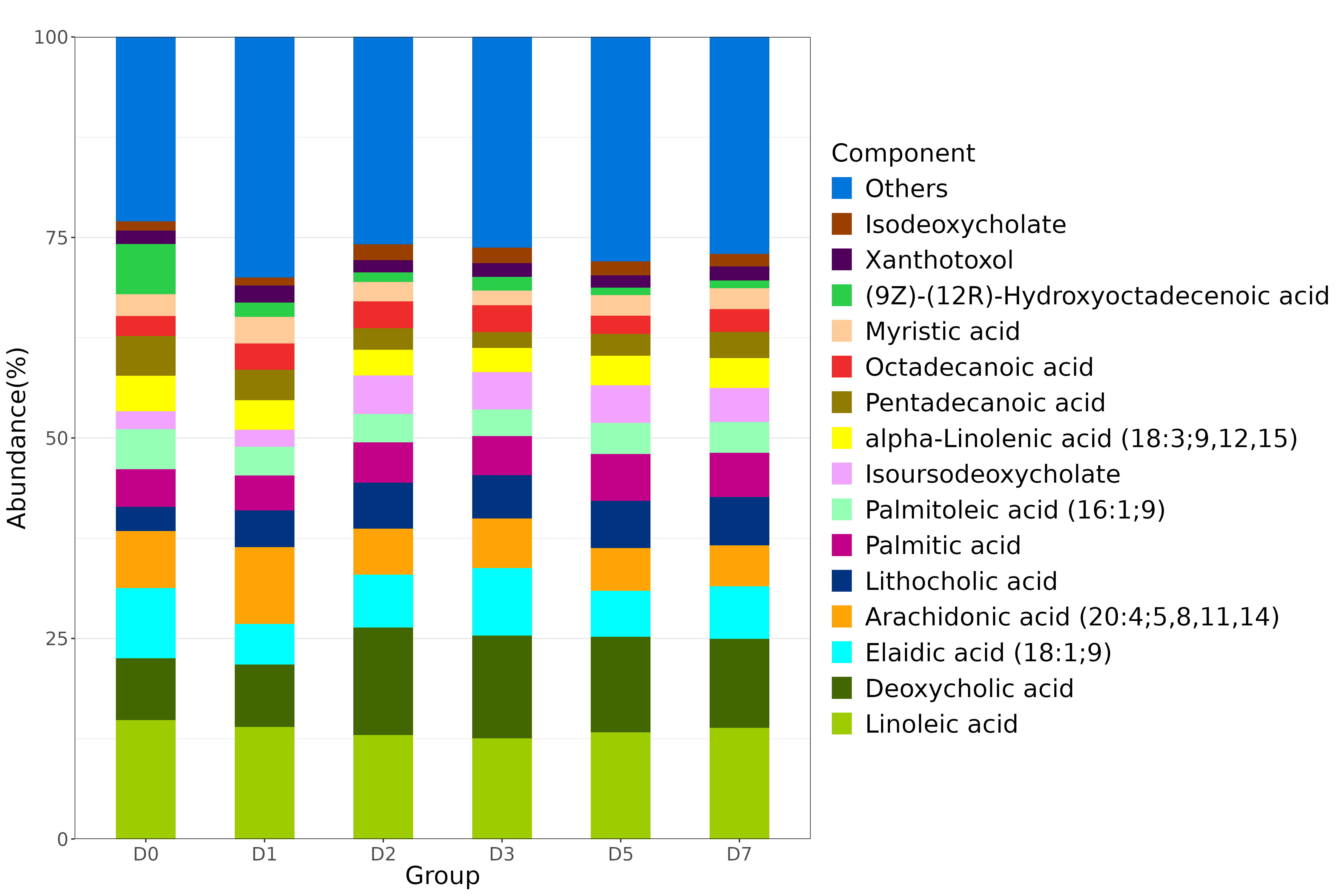

Supplement: Supplementary file 3 — Additional file 3. Raw data of the metabolomic compounds. [file 40104_2026_1385_MOESM3_ESM.zip › mix/KEGG_compound_summary/Barplot/compound_summary_level4_Group_barplot.png]

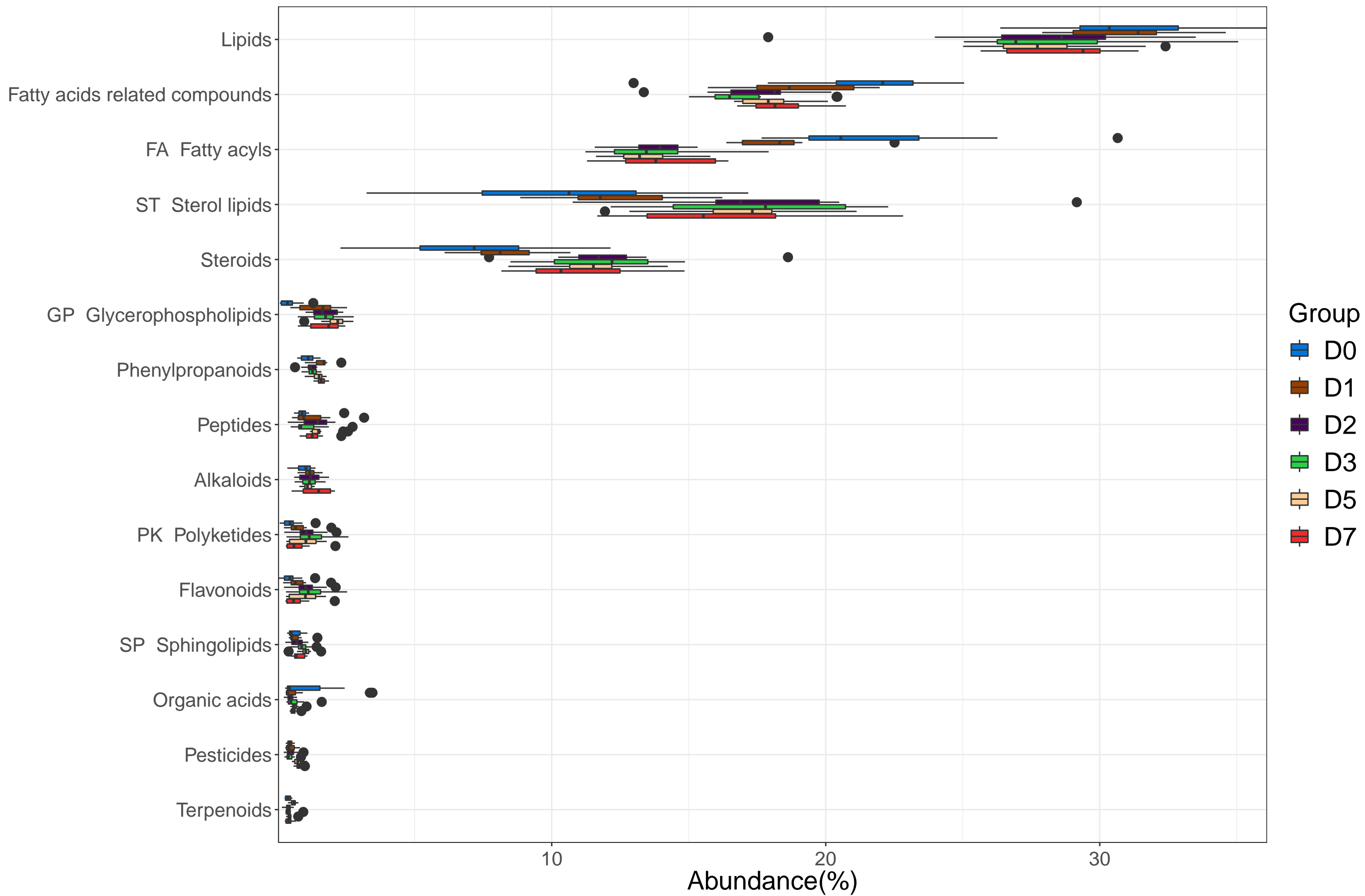

Supplement: Supplementary file 3 — Additional file 3. Raw data of the metabolomic compounds. [file 40104_2026_1385_MOESM3_ESM.zip › mix/KEGG_compound_summary/Boxplot/compound_summary_level1_boxplot.pdf]

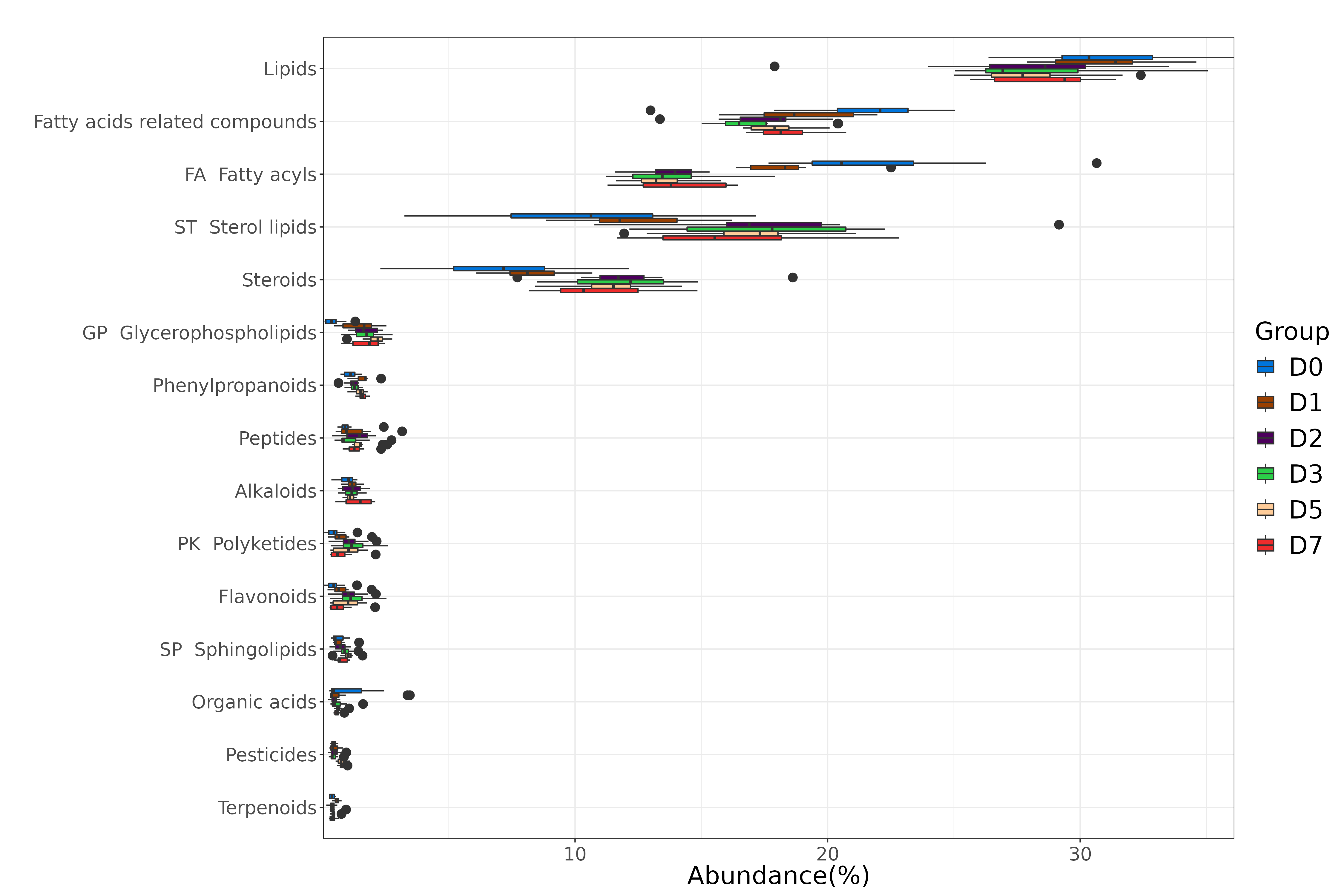

Supplement: Supplementary file 3 — Additional file 3. Raw data of the metabolomic compounds. [file 40104_2026_1385_MOESM3_ESM.zip › mix/KEGG_compound_summary/Boxplot/compound_summary_level1_boxplot.png]

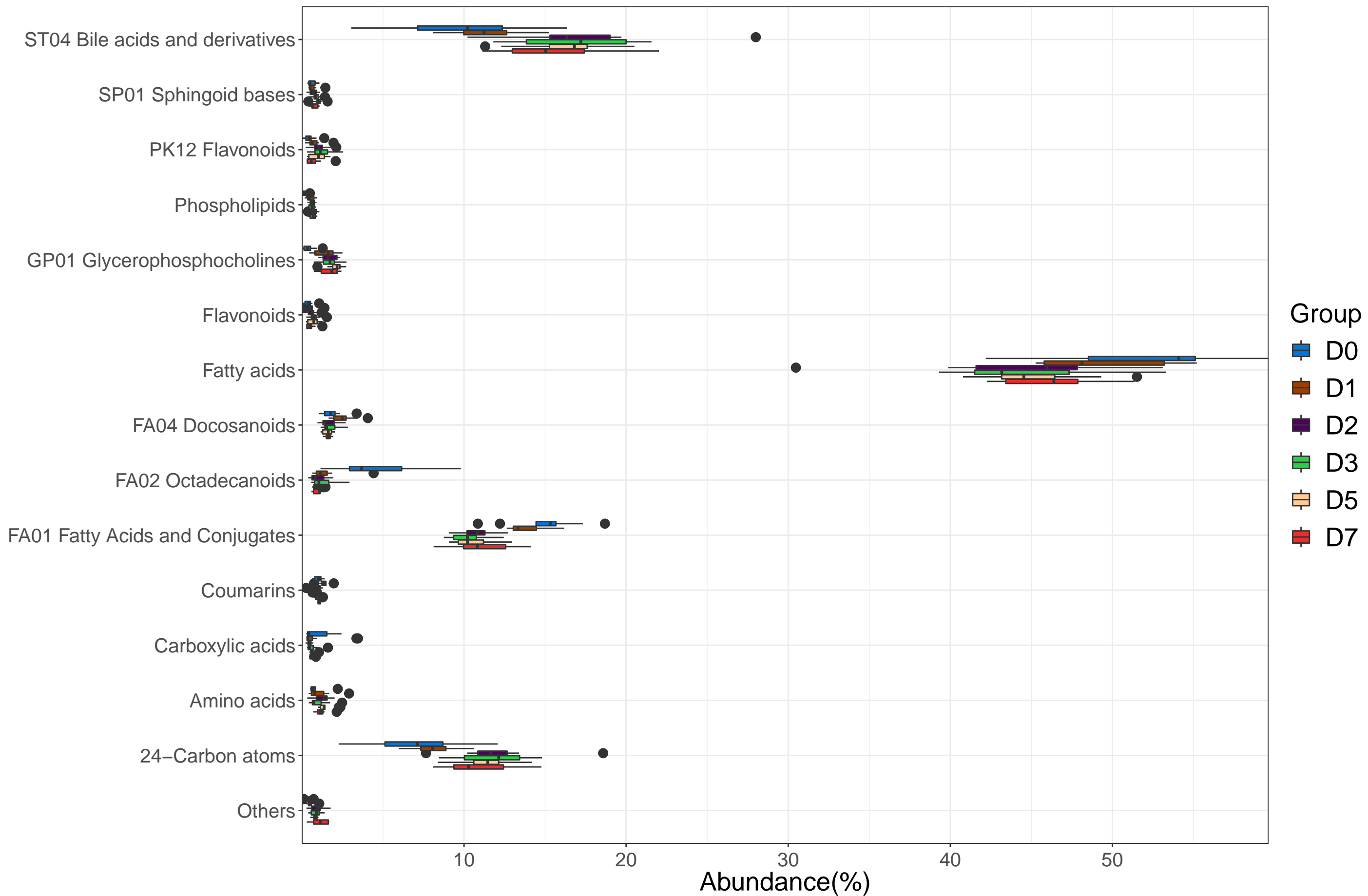

Supplement: Supplementary file 3 — Additional file 3. Raw data of the metabolomic compounds. [file 40104_2026_1385_MOESM3_ESM.zip › mix/KEGG_compound_summary/Boxplot/compound_summary_level2_boxplot.pdf]

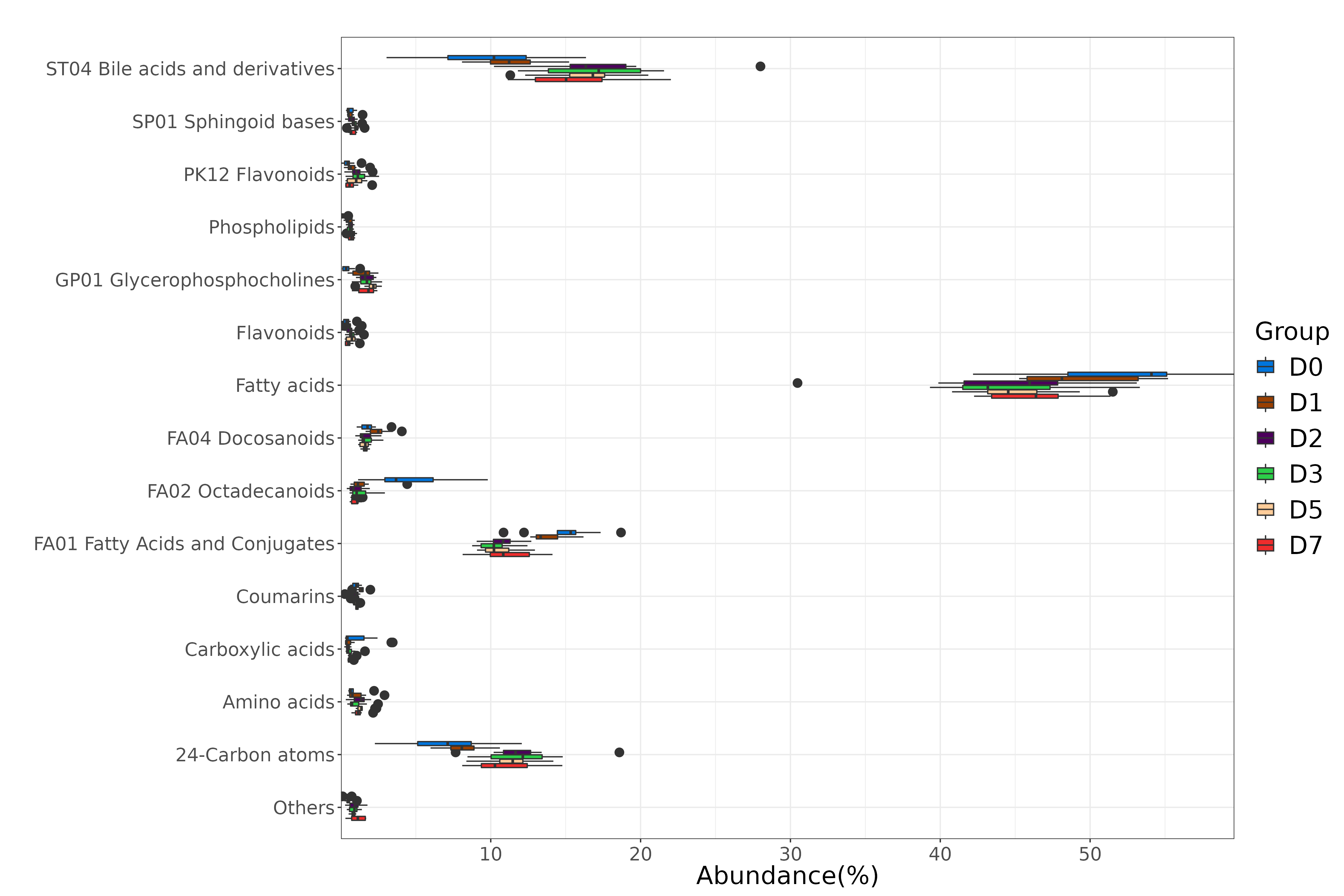

Supplement: Supplementary file 3 — Additional file 3. Raw data of the metabolomic compounds. [file 40104_2026_1385_MOESM3_ESM.zip › mix/KEGG_compound_summary/Boxplot/compound_summary_level2_boxplot.png]

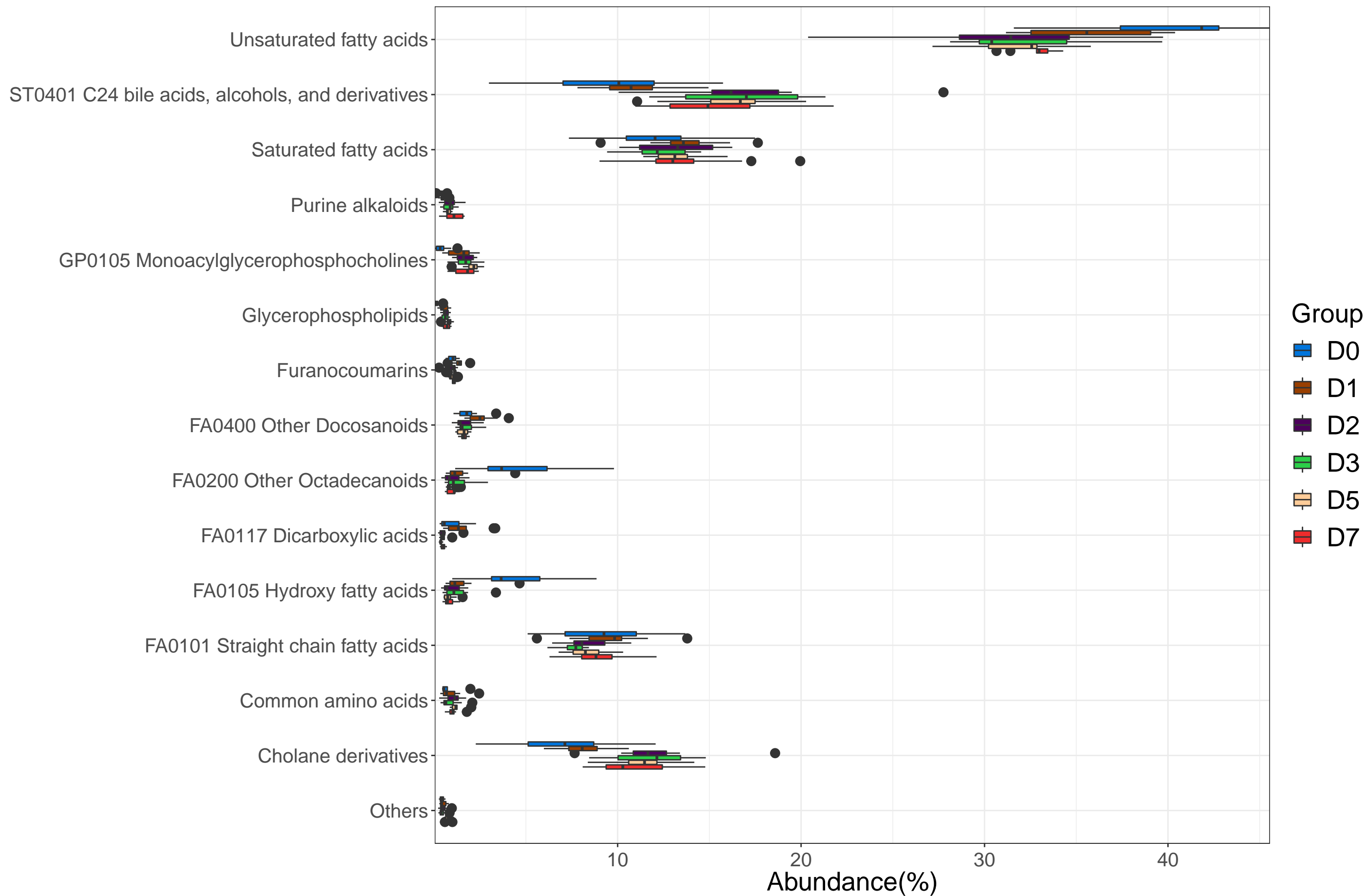

Supplement: Supplementary file 3 — Additional file 3. Raw data of the metabolomic compounds. [file 40104_2026_1385_MOESM3_ESM.zip › mix/KEGG_compound_summary/Boxplot/compound_summary_level3_boxplot.pdf]

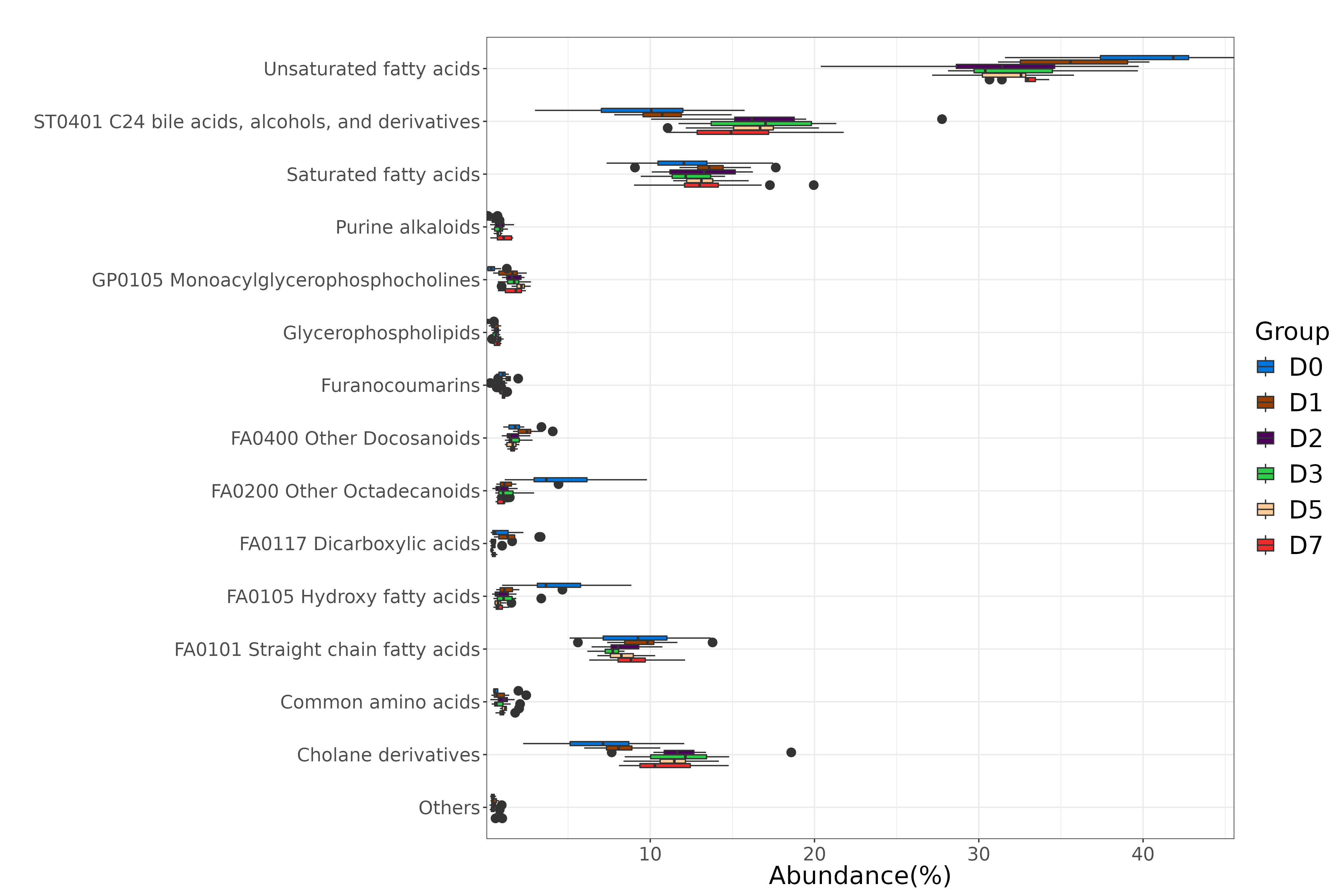

Supplement: Supplementary file 3 — Additional file 3. Raw data of the metabolomic compounds. [file 40104_2026_1385_MOESM3_ESM.zip › mix/KEGG_compound_summary/Boxplot/compound_summary_level3_boxplot.png]

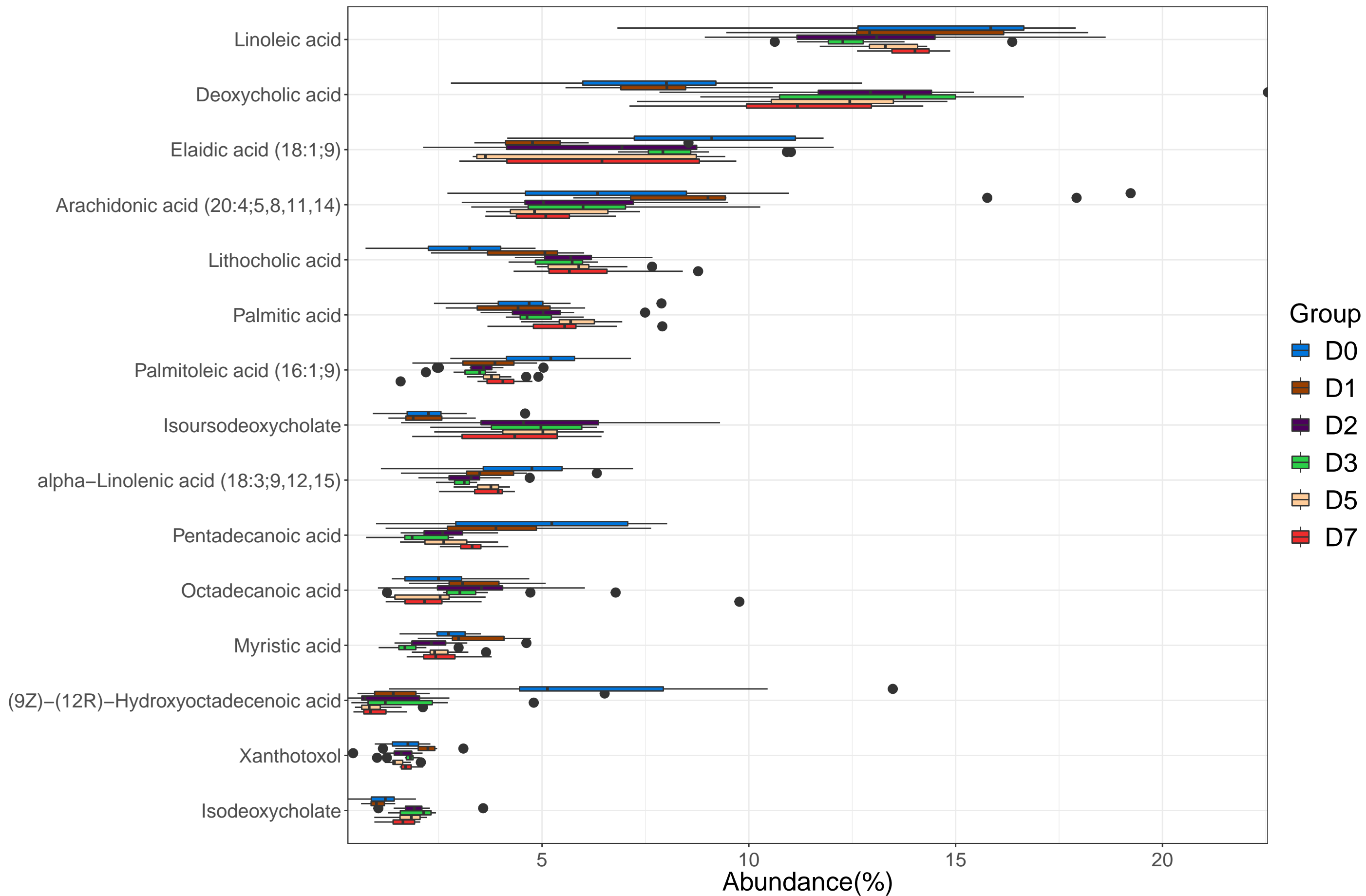

Supplement: Supplementary file 3 — Additional file 3. Raw data of the metabolomic compounds. [file 40104_2026_1385_MOESM3_ESM.zip › mix/KEGG_compound_summary/Boxplot/compound_summary_level4_boxplot.pdf]

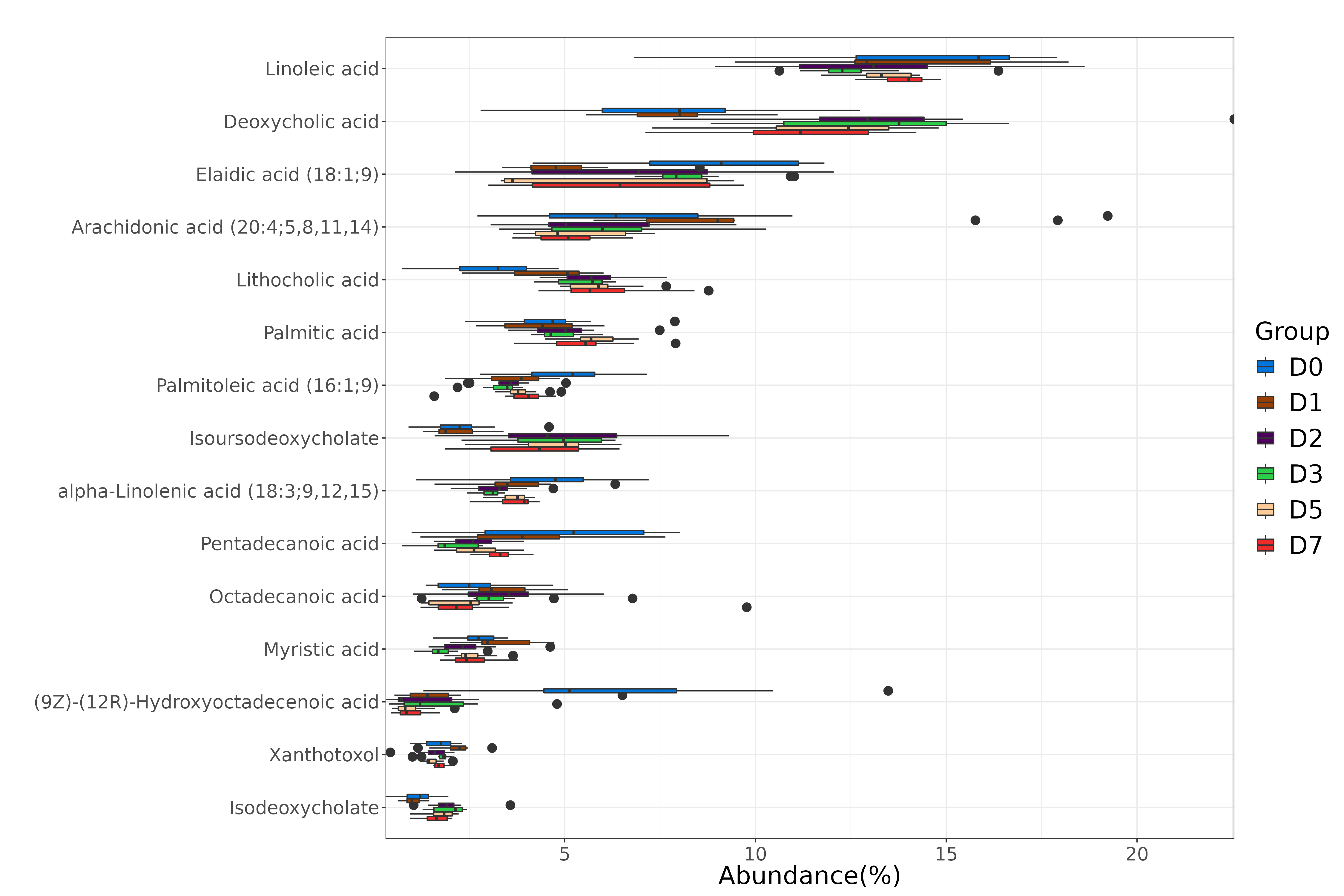

Supplement: Supplementary file 3 — Additional file 3. Raw data of the metabolomic compounds. [file 40104_2026_1385_MOESM3_ESM.zip › mix/KEGG_compound_summary/Boxplot/compound_summary_level4_boxplot.png]

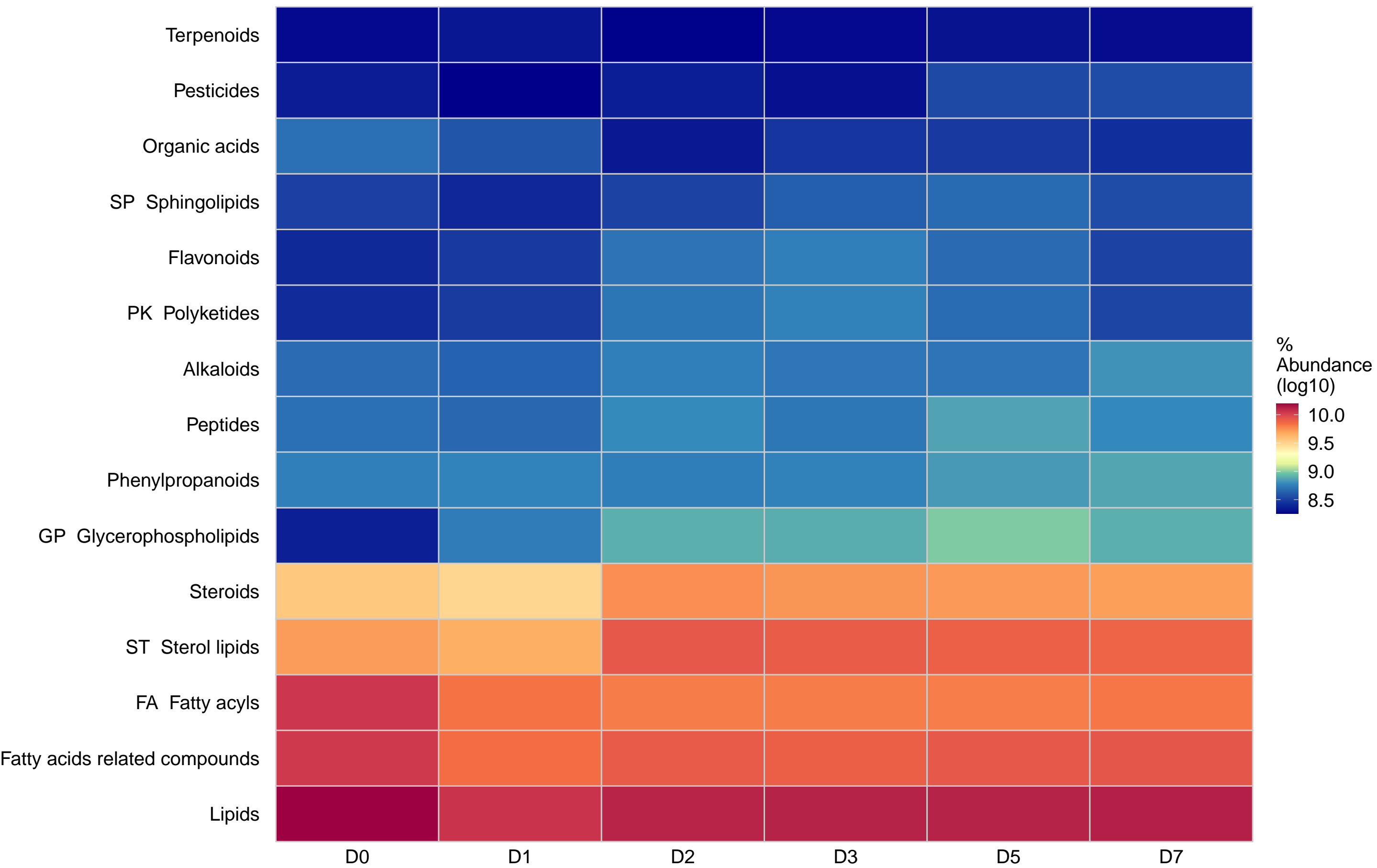

Supplement: Supplementary file 3 — Additional file 3. Raw data of the metabolomic compounds. [file 40104_2026_1385_MOESM3_ESM.zip › mix/KEGG_compound_summary/Heatmap/compound_summary_level1_Group_heatmap.pdf]

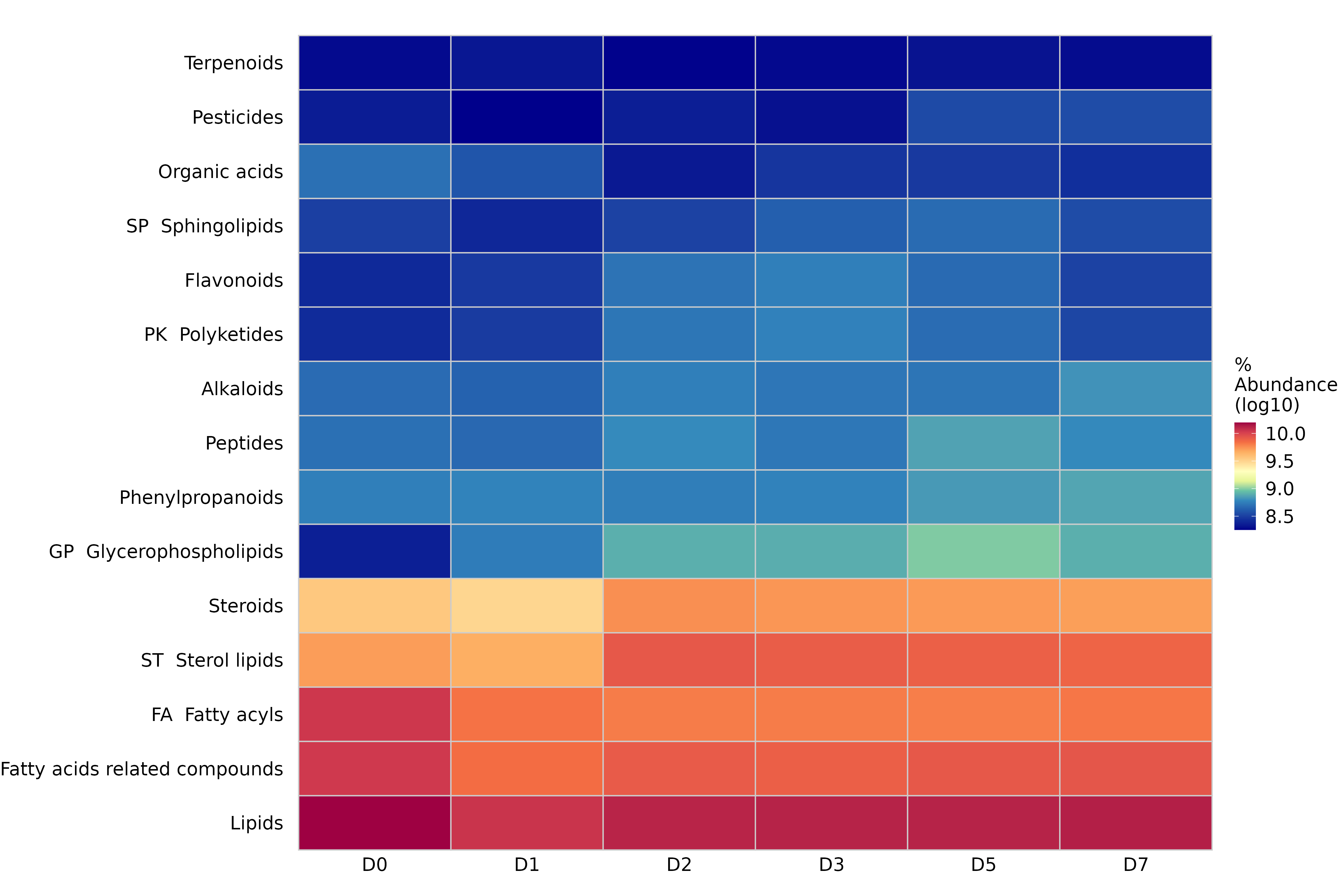

Supplement: Supplementary file 3 — Additional file 3. Raw data of the metabolomic compounds. [file 40104_2026_1385_MOESM3_ESM.zip › mix/KEGG_compound_summary/Heatmap/compound_summary_level1_Group_heatmap.png]

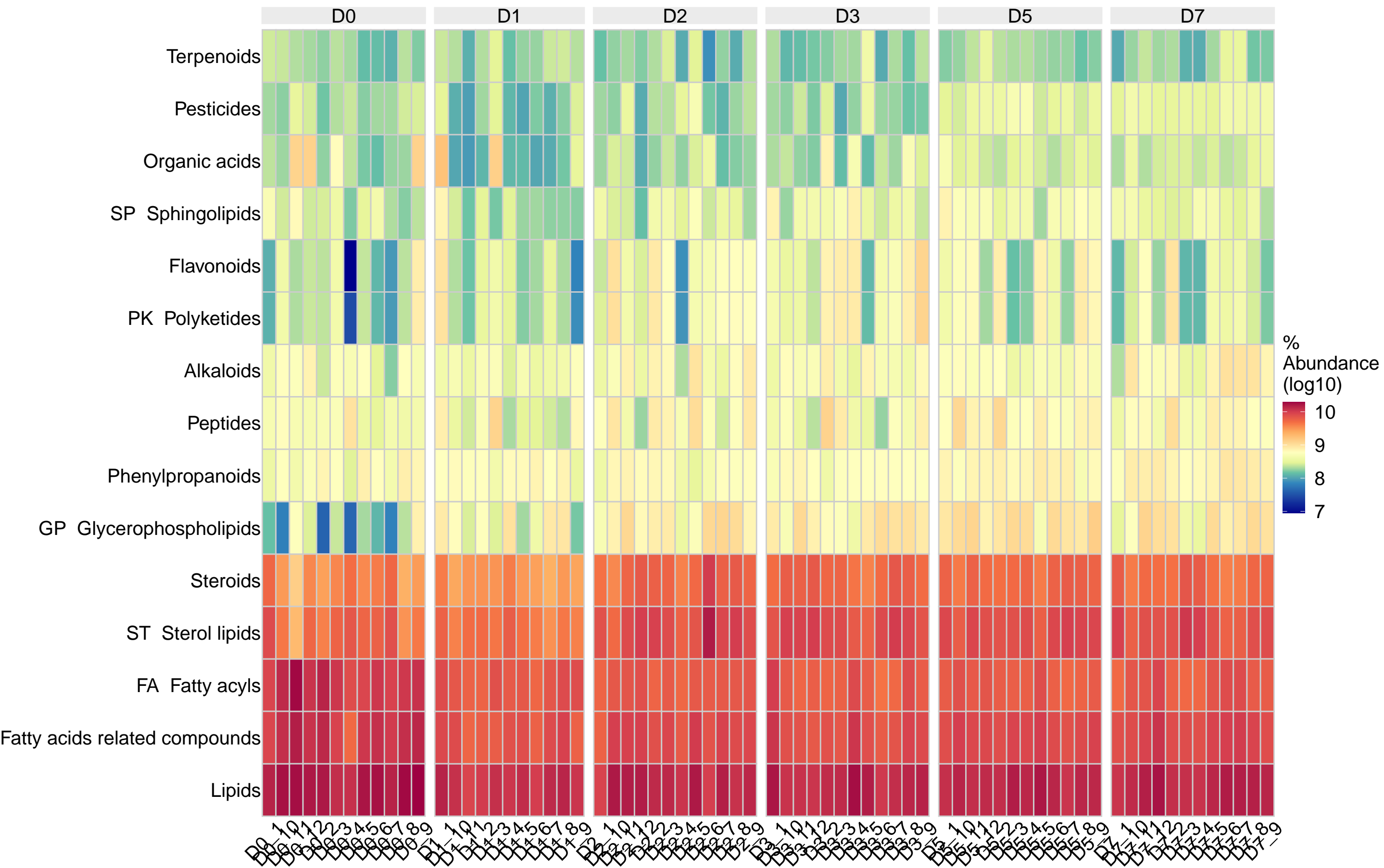

Supplement: Supplementary file 3 — Additional file 3. Raw data of the metabolomic compounds. [file 40104_2026_1385_MOESM3_ESM.zip › mix/KEGG_compound_summary/Heatmap/compound_summary_level1_heatmap.pdf]

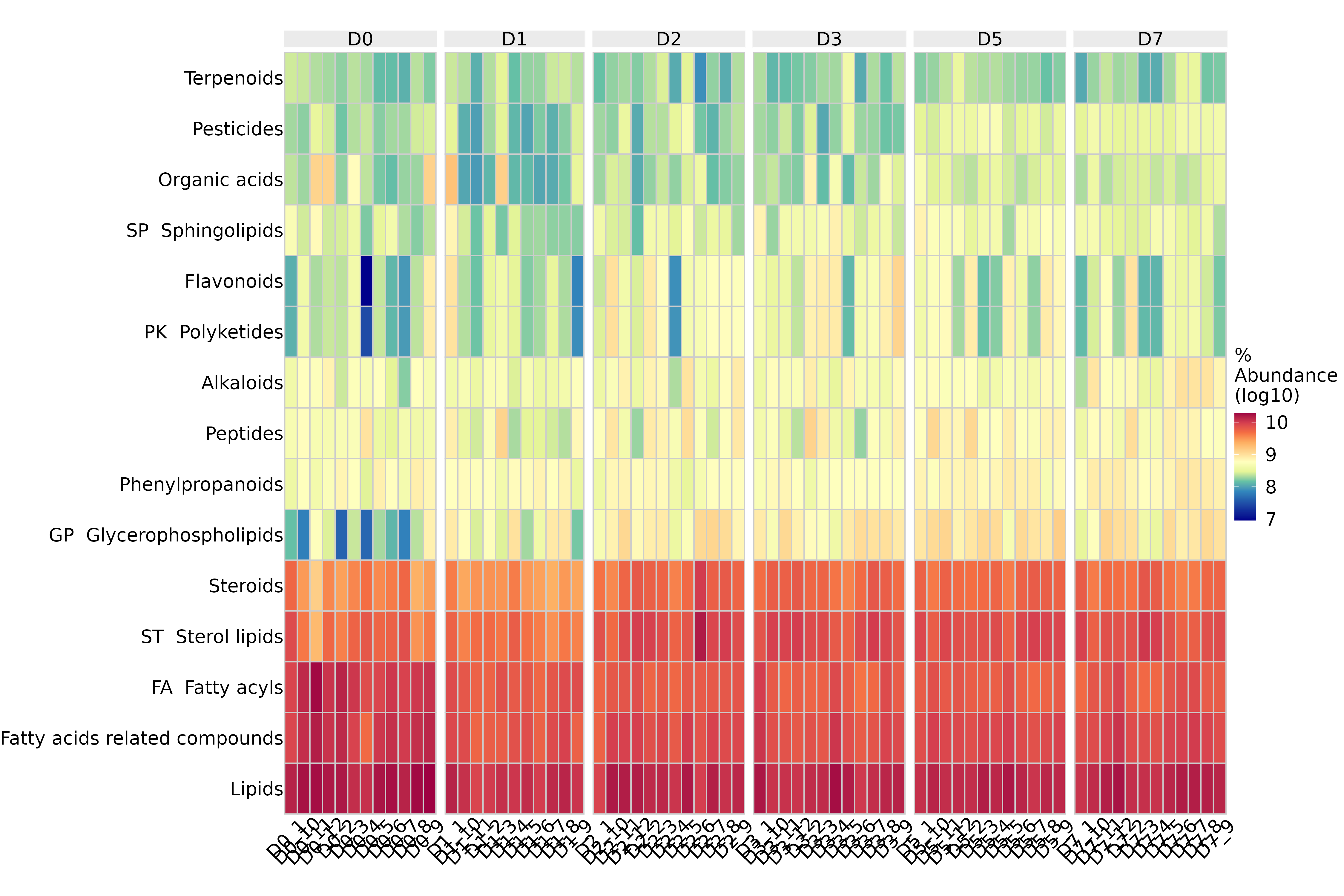

Supplement: Supplementary file 3 — Additional file 3. Raw data of the metabolomic compounds. [file 40104_2026_1385_MOESM3_ESM.zip › mix/KEGG_compound_summary/Heatmap/compound_summary_level1_heatmap.png]

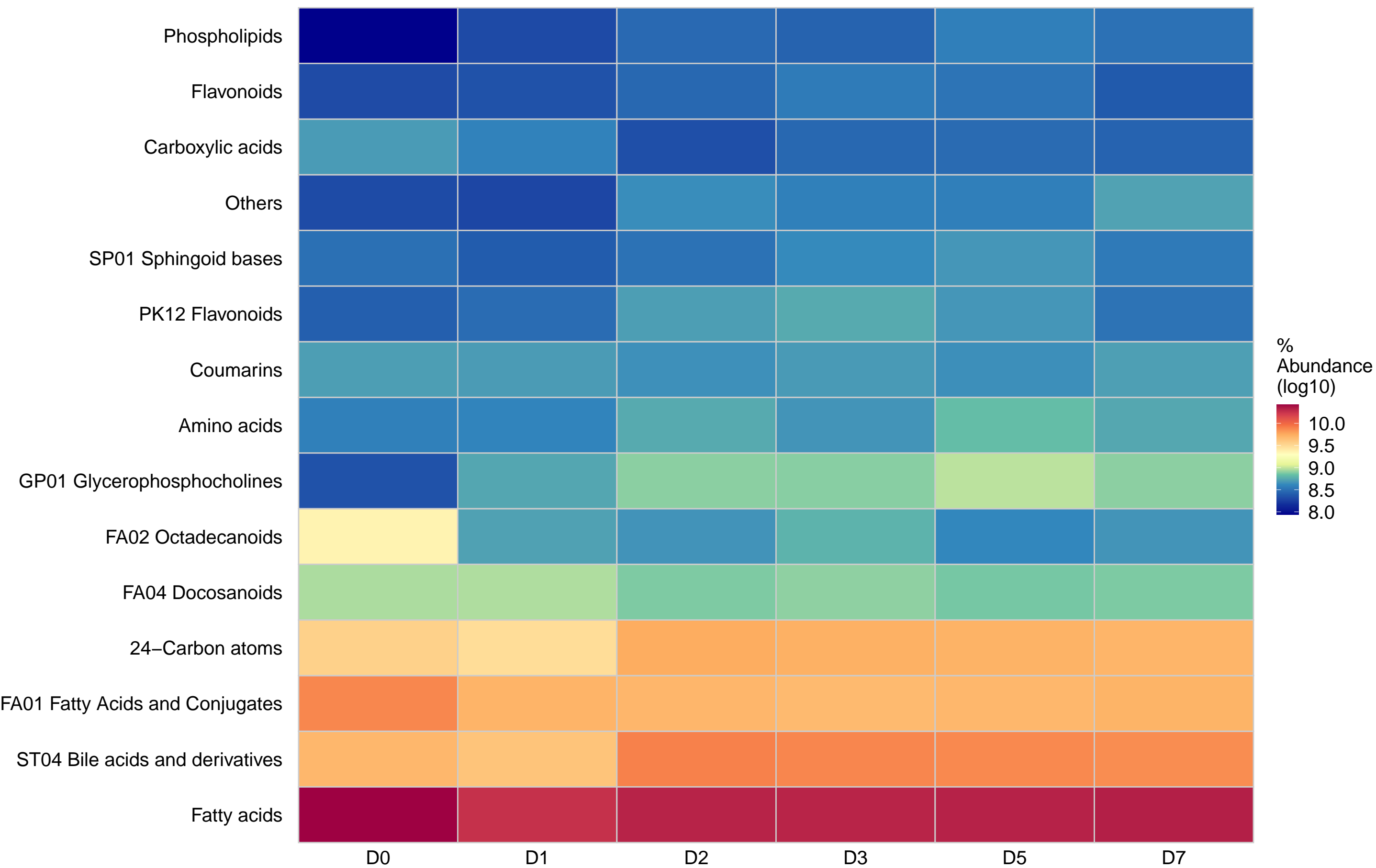

Supplement: Supplementary file 3 — Additional file 3. Raw data of the metabolomic compounds. [file 40104_2026_1385_MOESM3_ESM.zip › mix/KEGG_compound_summary/Heatmap/compound_summary_level2_Group_heatmap.pdf]

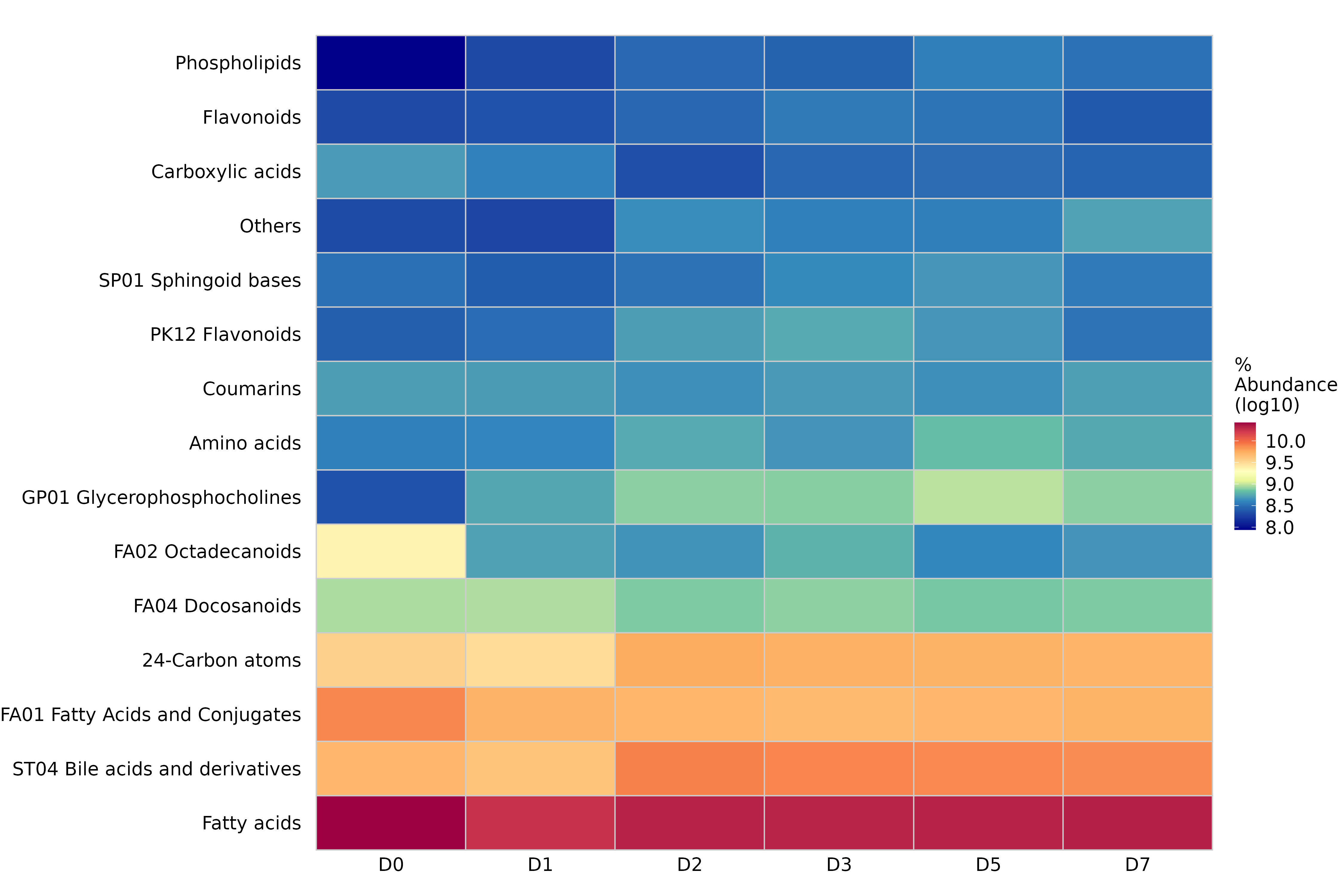

Supplement: Supplementary file 3 — Additional file 3. Raw data of the metabolomic compounds. [file 40104_2026_1385_MOESM3_ESM.zip › mix/KEGG_compound_summary/Heatmap/compound_summary_level2_Group_heatmap.png]

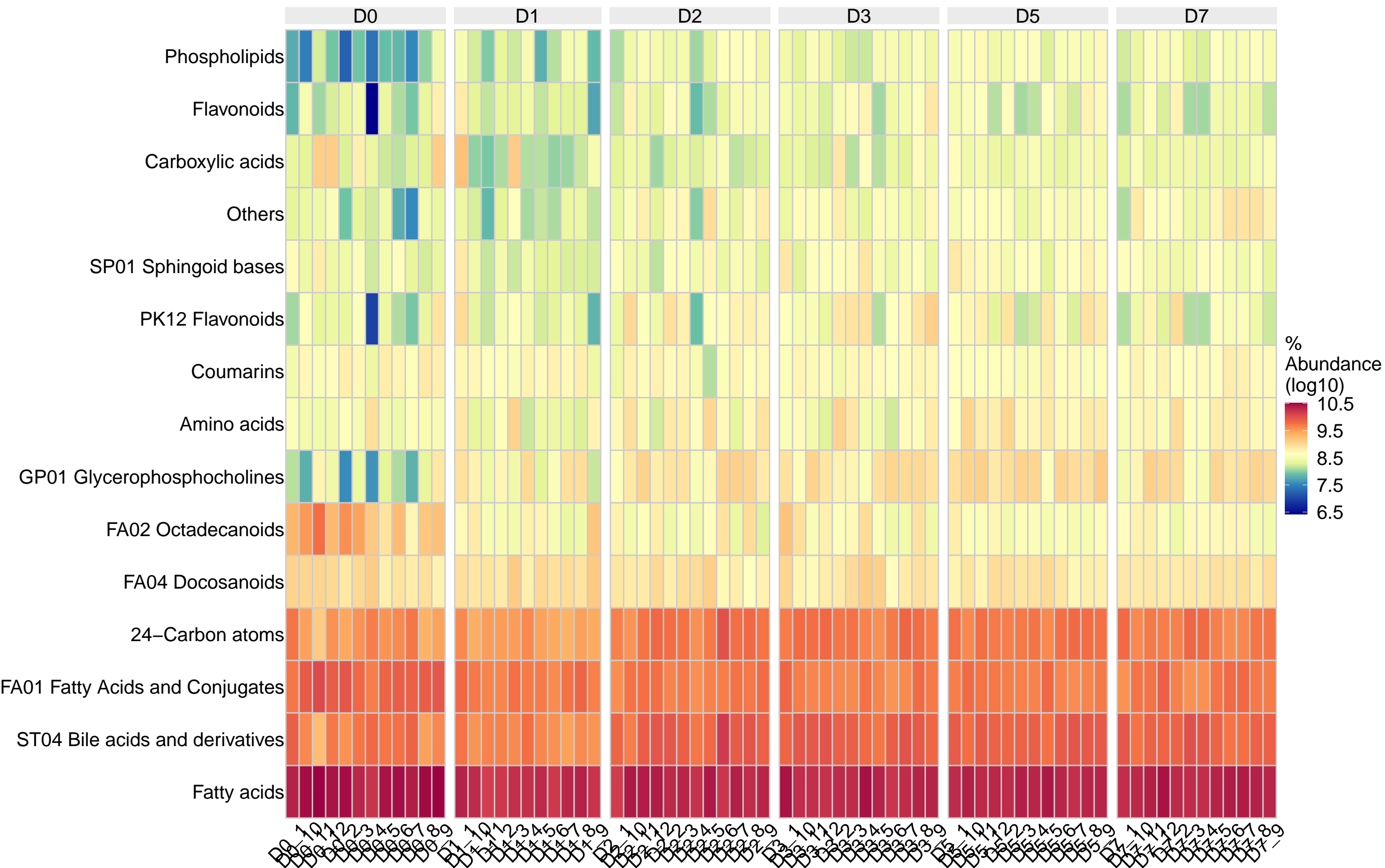

Supplement: Supplementary file 3 — Additional file 3. Raw data of the metabolomic compounds. [file 40104_2026_1385_MOESM3_ESM.zip › mix/KEGG_compound_summary/Heatmap/compound_summary_level2_heatmap.pdf]

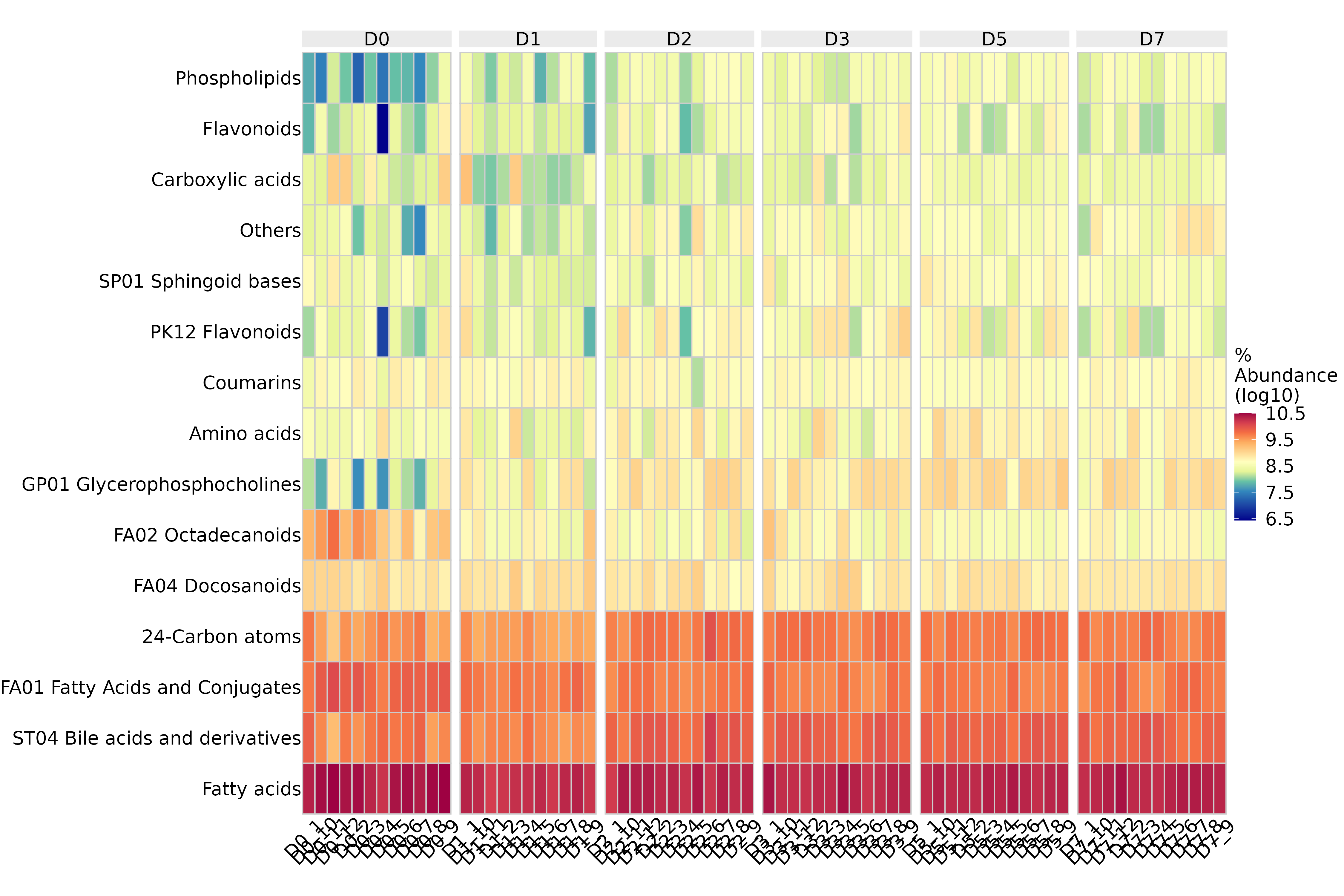

Supplement: Supplementary file 3 — Additional file 3. Raw data of the metabolomic compounds. [file 40104_2026_1385_MOESM3_ESM.zip › mix/KEGG_compound_summary/Heatmap/compound_summary_level2_heatmap.png]

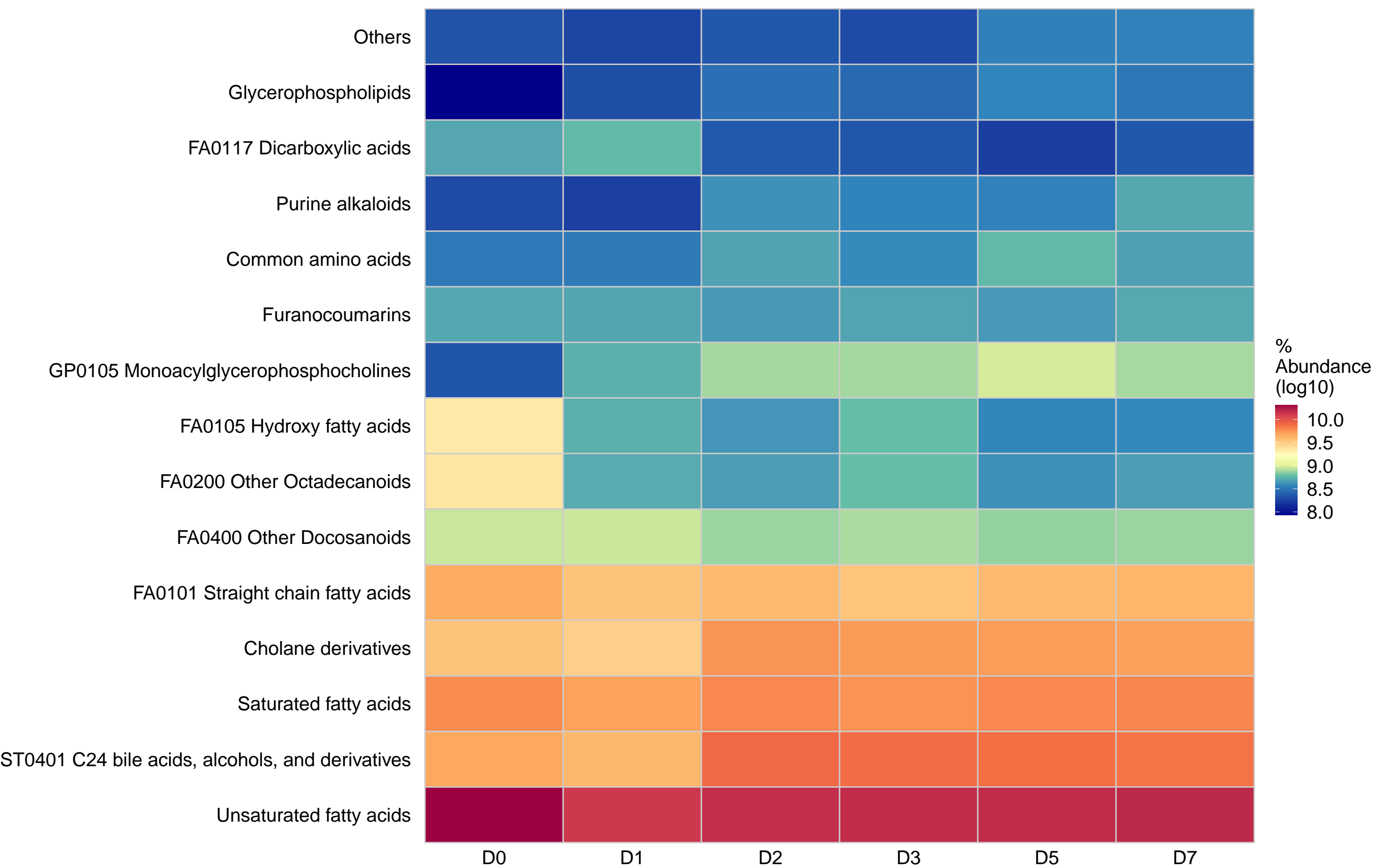

Supplement: Supplementary file 3 — Additional file 3. Raw data of the metabolomic compounds. [file 40104_2026_1385_MOESM3_ESM.zip › mix/KEGG_compound_summary/Heatmap/compound_summary_level3_Group_heatmap.pdf]

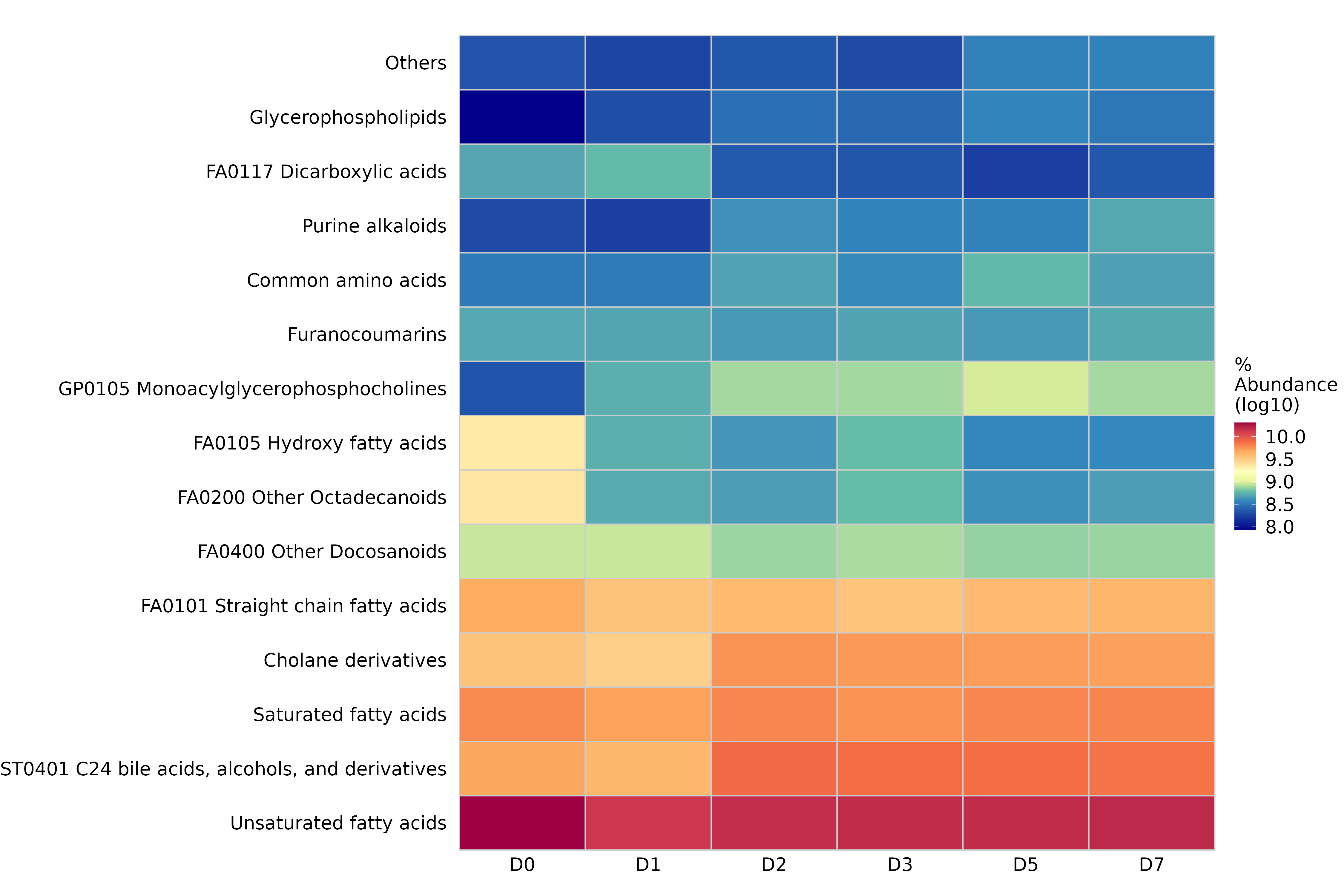

Supplement: Supplementary file 3 — Additional file 3. Raw data of the metabolomic compounds. [file 40104_2026_1385_MOESM3_ESM.zip › mix/KEGG_compound_summary/Heatmap/compound_summary_level3_Group_heatmap.png]

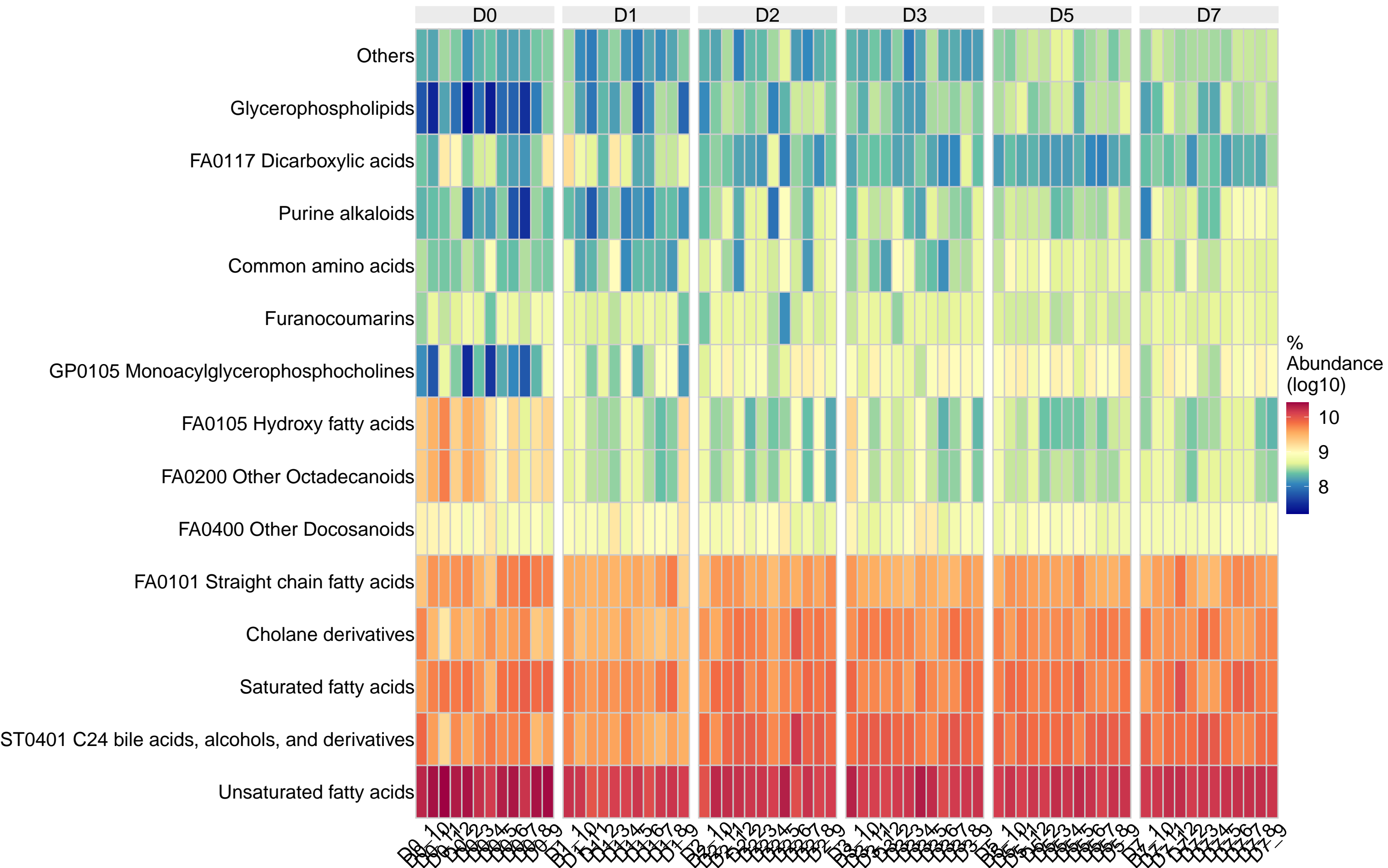

Supplement: Supplementary file 3 — Additional file 3. Raw data of the metabolomic compounds. [file 40104_2026_1385_MOESM3_ESM.zip › mix/KEGG_compound_summary/Heatmap/compound_summary_level3_heatmap.pdf]

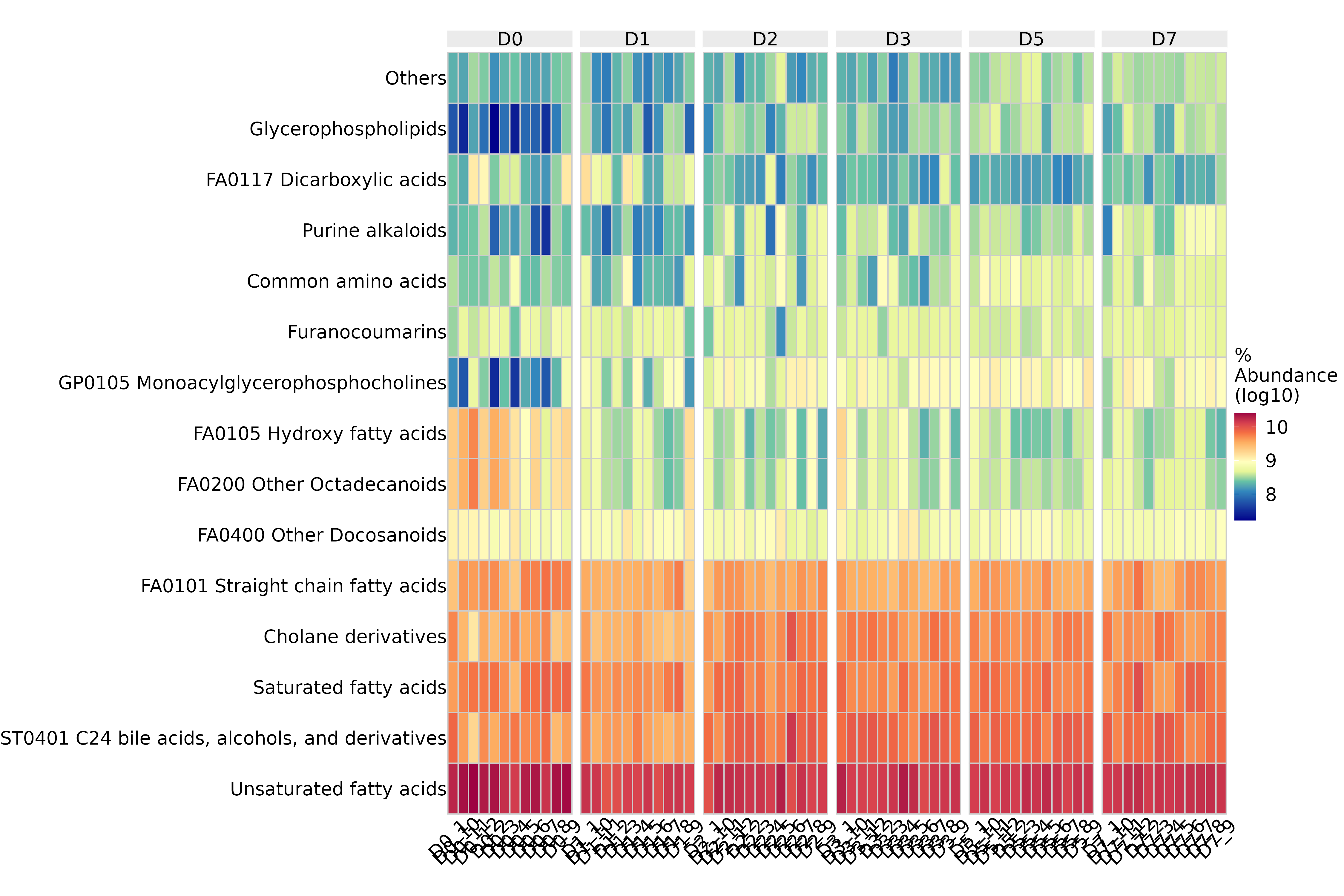

Supplement: Supplementary file 3 — Additional file 3. Raw data of the metabolomic compounds. [file 40104_2026_1385_MOESM3_ESM.zip › mix/KEGG_compound_summary/Heatmap/compound_summary_level3_heatmap.png]

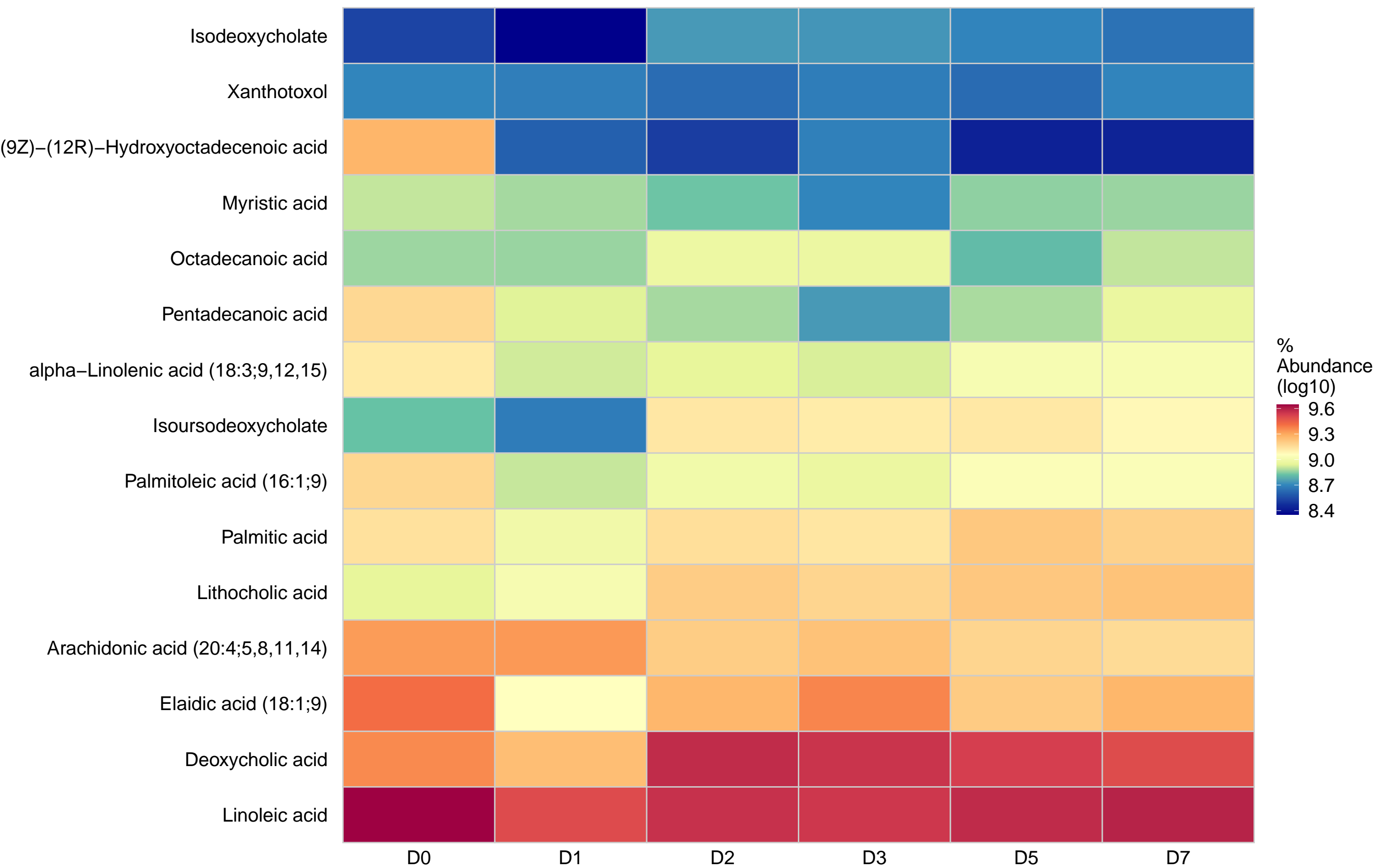

Supplement: Supplementary file 3 — Additional file 3. Raw data of the metabolomic compounds. [file 40104_2026_1385_MOESM3_ESM.zip › mix/KEGG_compound_summary/Heatmap/compound_summary_level4_Group_heatmap.pdf]

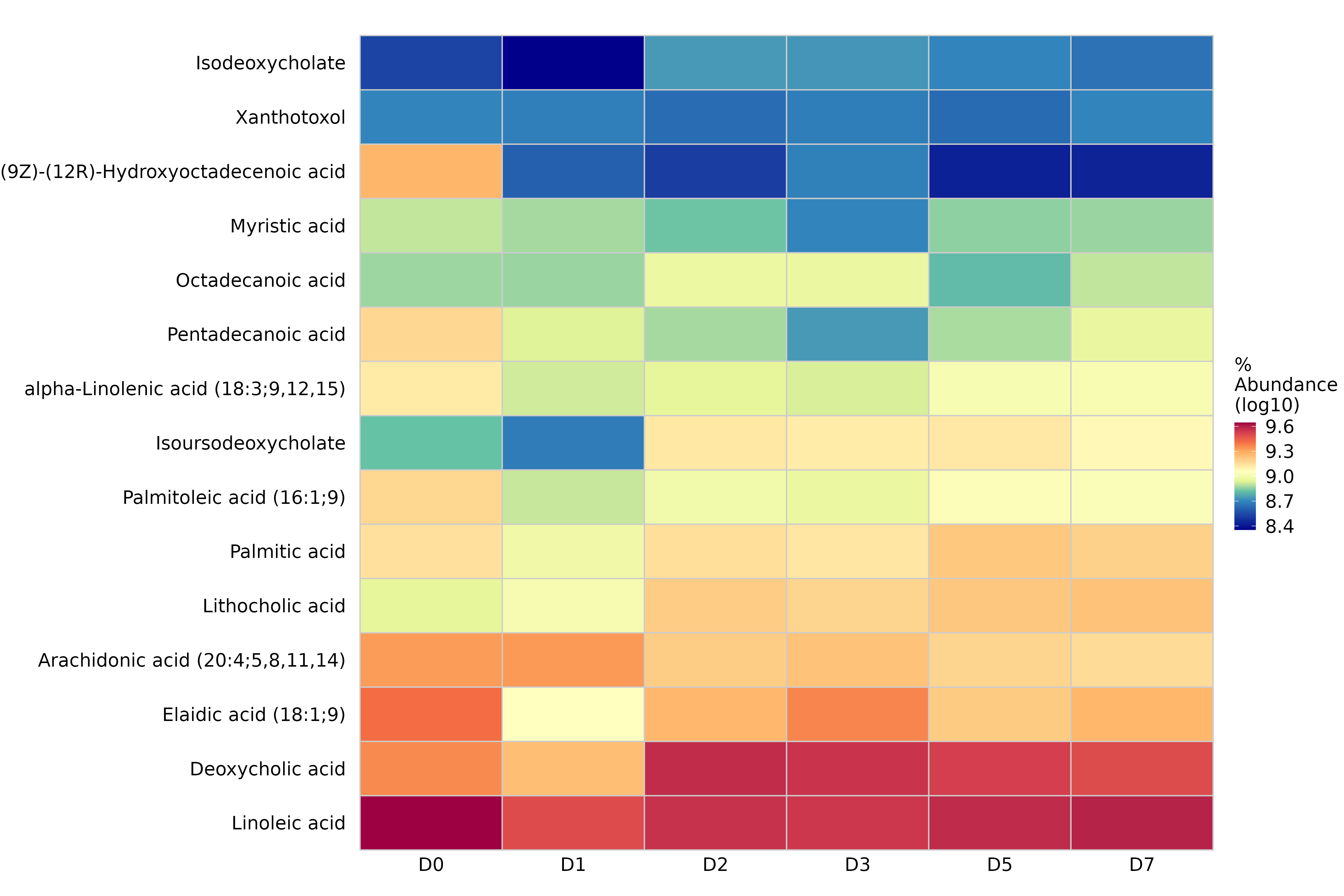

Supplement: Supplementary file 3 — Additional file 3. Raw data of the metabolomic compounds. [file 40104_2026_1385_MOESM3_ESM.zip › mix/KEGG_compound_summary/Heatmap/compound_summary_level4_Group_heatmap.png]

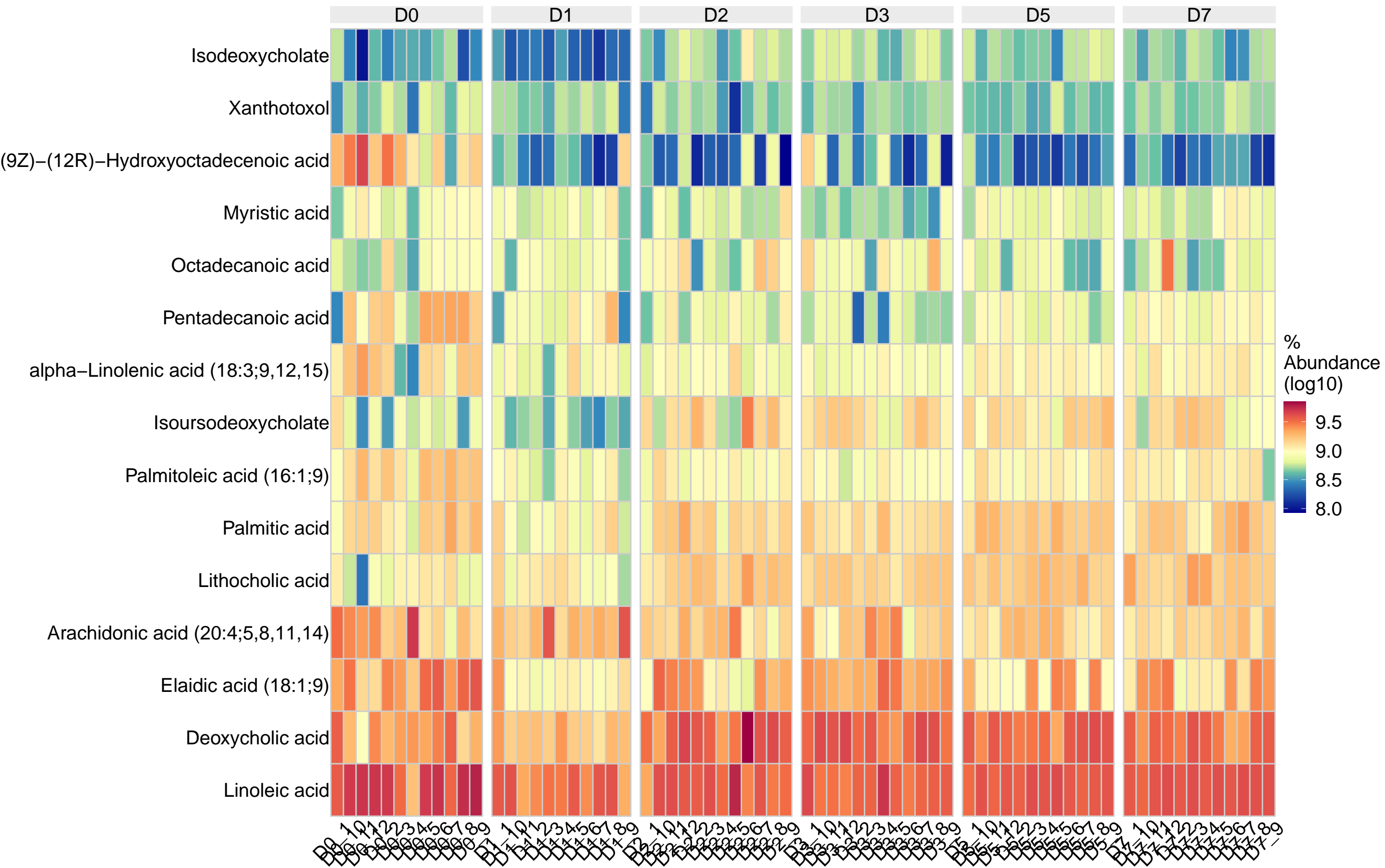

Supplement: Supplementary file 3 — Additional file 3. Raw data of the metabolomic compounds. [file 40104_2026_1385_MOESM3_ESM.zip › mix/KEGG_compound_summary/Heatmap/compound_summary_level4_heatmap.pdf]

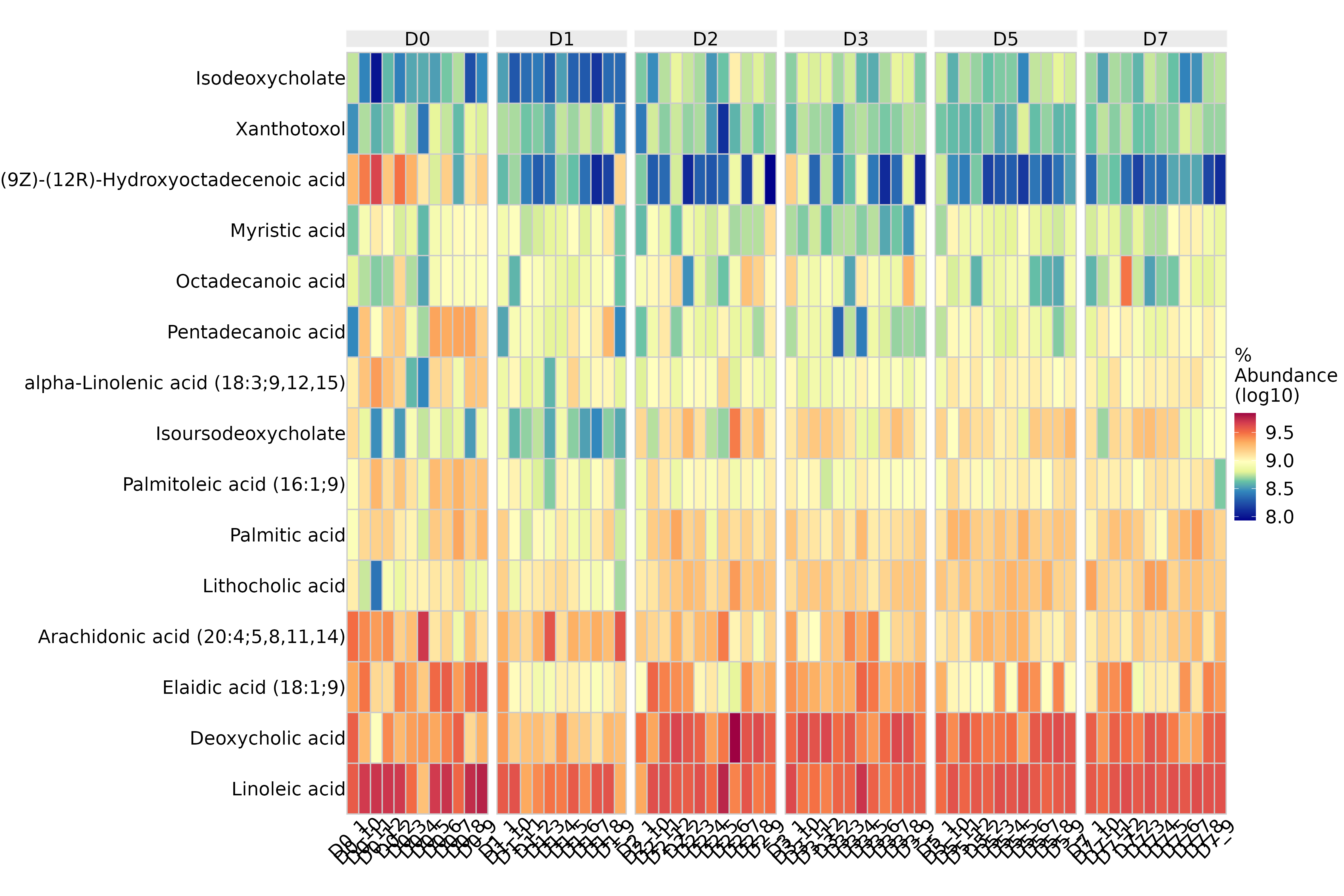

Supplement: Supplementary file 3 — Additional file 3. Raw data of the metabolomic compounds. [file 40104_2026_1385_MOESM3_ESM.zip › mix/KEGG_compound_summary/Heatmap/compound_summary_level4_heatmap.png]

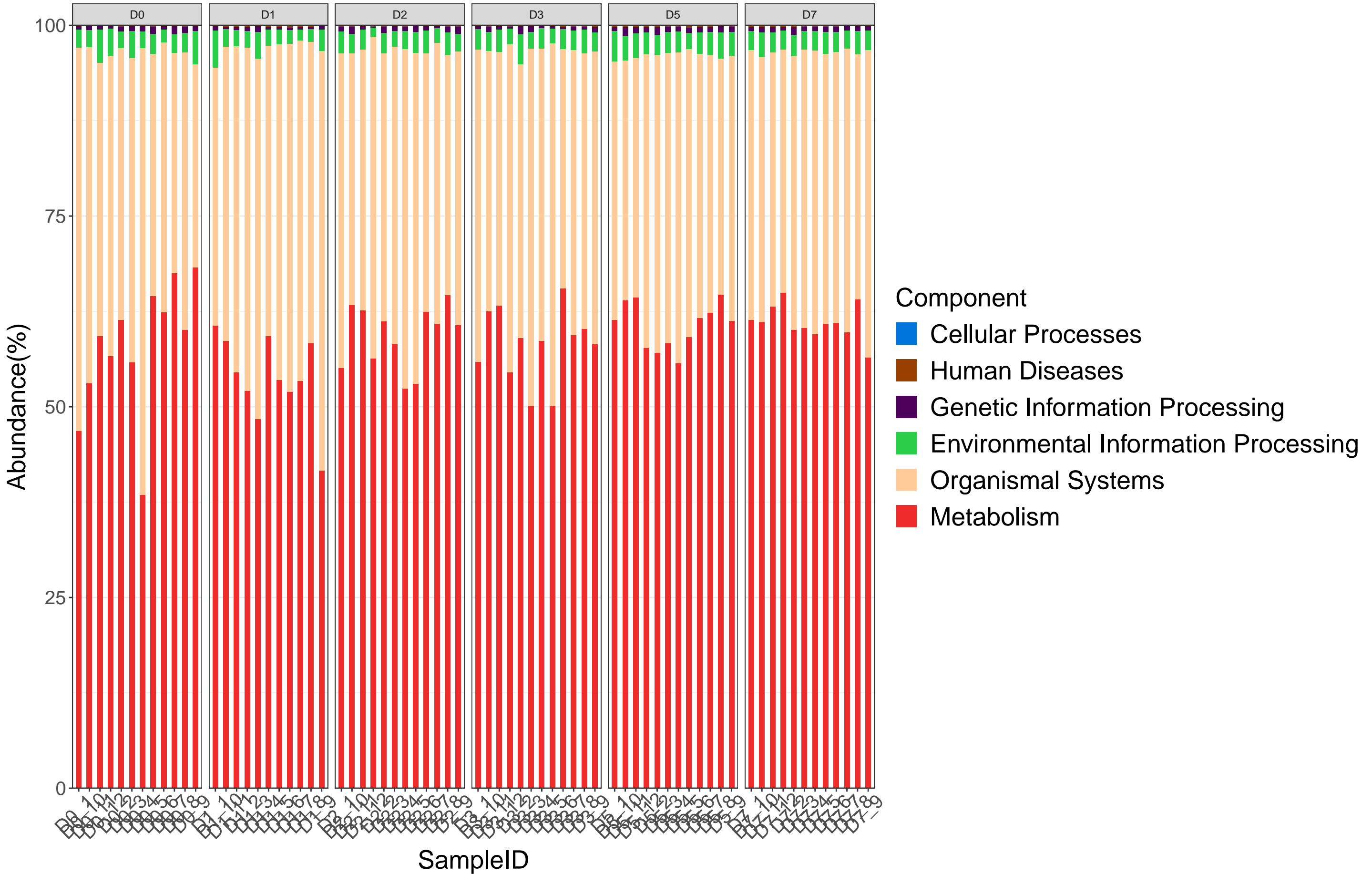

Supplement: Supplementary file 3 — Additional file 3. Raw data of the metabolomic compounds. [file 40104_2026_1385_MOESM3_ESM.zip › mix/KEGG_function_summary/Barplot/function_summary_level1_barplot.pdf]

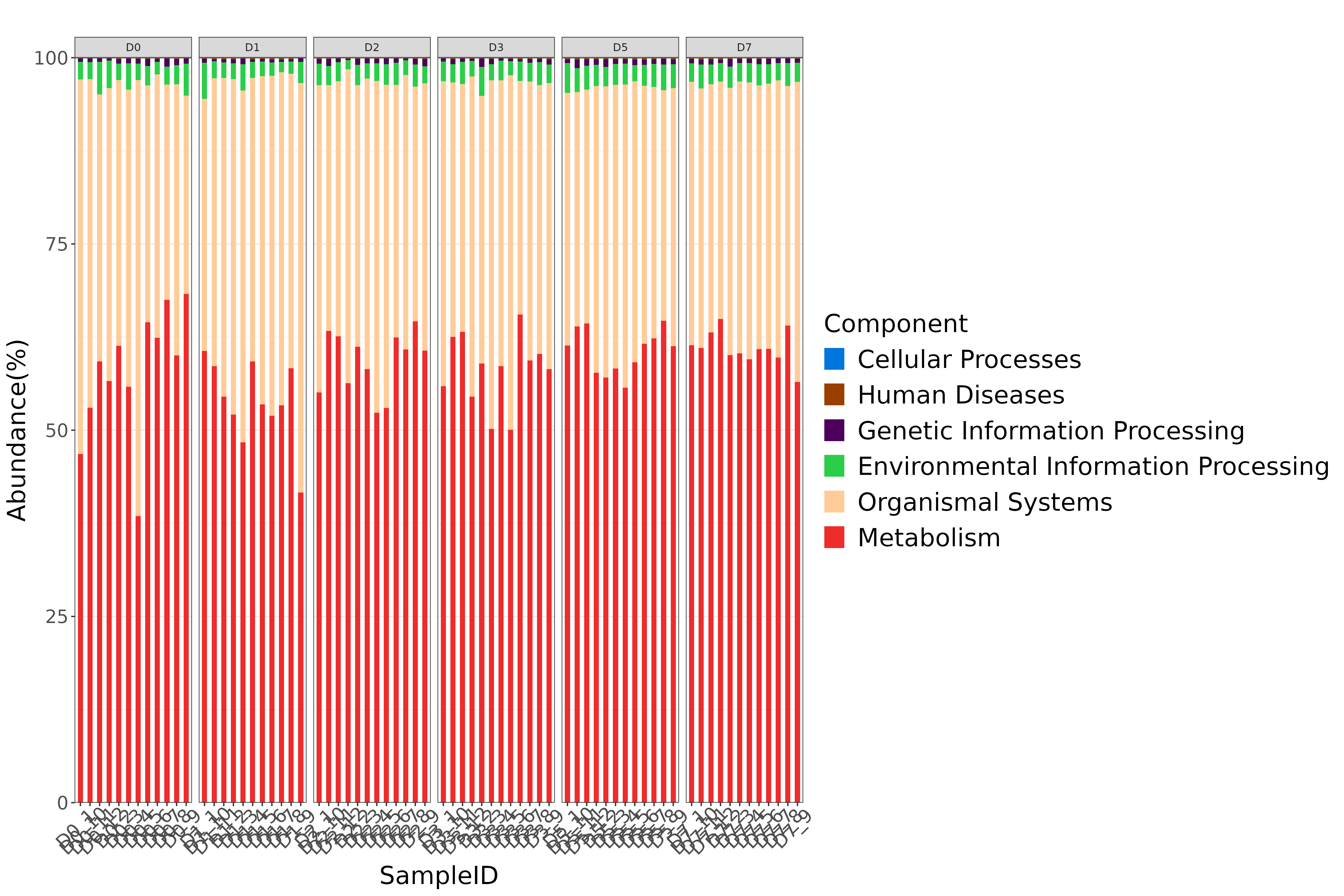

Supplement: Supplementary file 3 — Additional file 3. Raw data of the metabolomic compounds. [file 40104_2026_1385_MOESM3_ESM.zip › mix/KEGG_function_summary/Barplot/function_summary_level1_barplot.png]

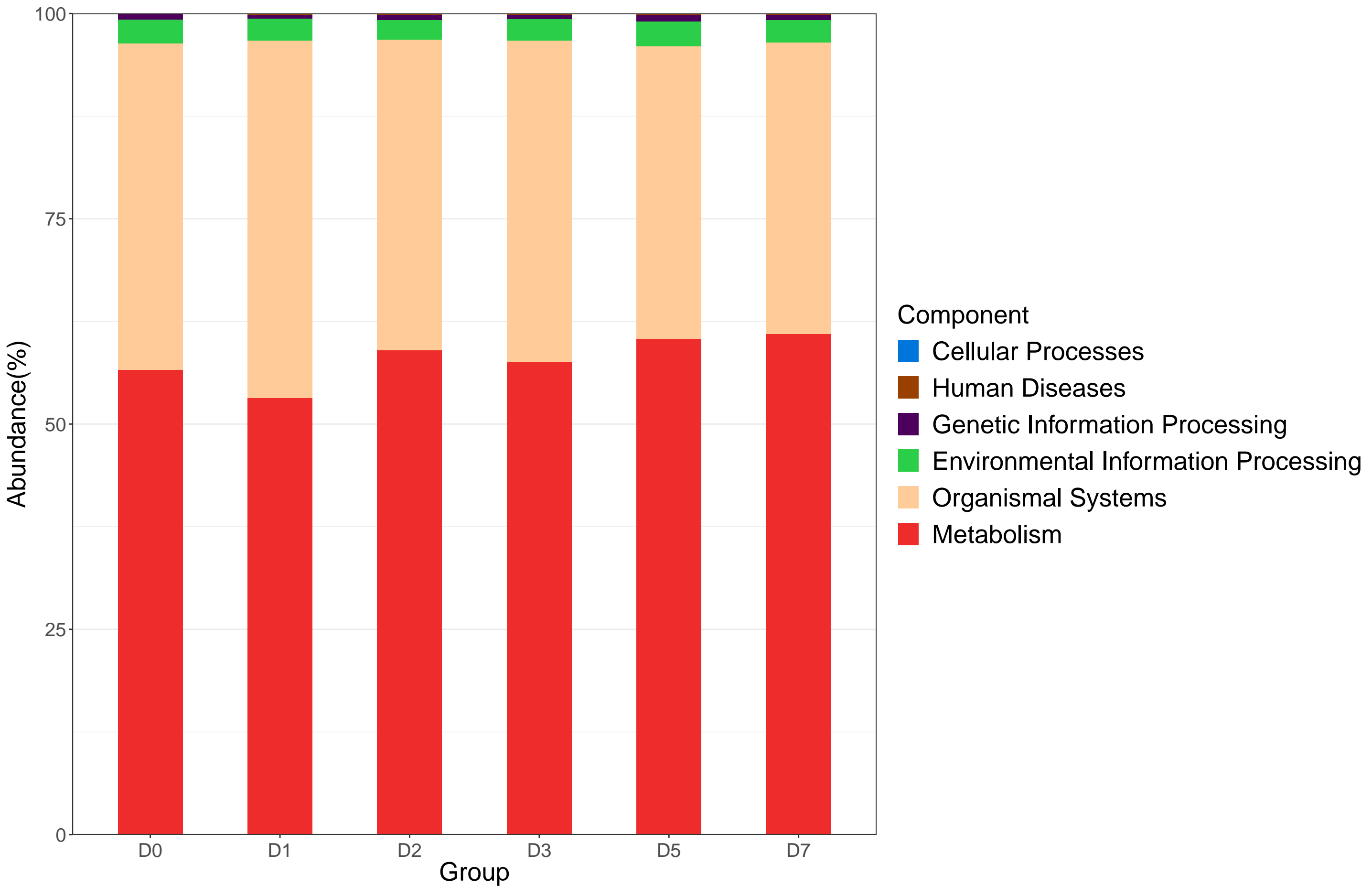

Supplement: Supplementary file 3 — Additional file 3. Raw data of the metabolomic compounds. [file 40104_2026_1385_MOESM3_ESM.zip › mix/KEGG_function_summary/Barplot/function_summary_level1_Group_barplot.pdf]

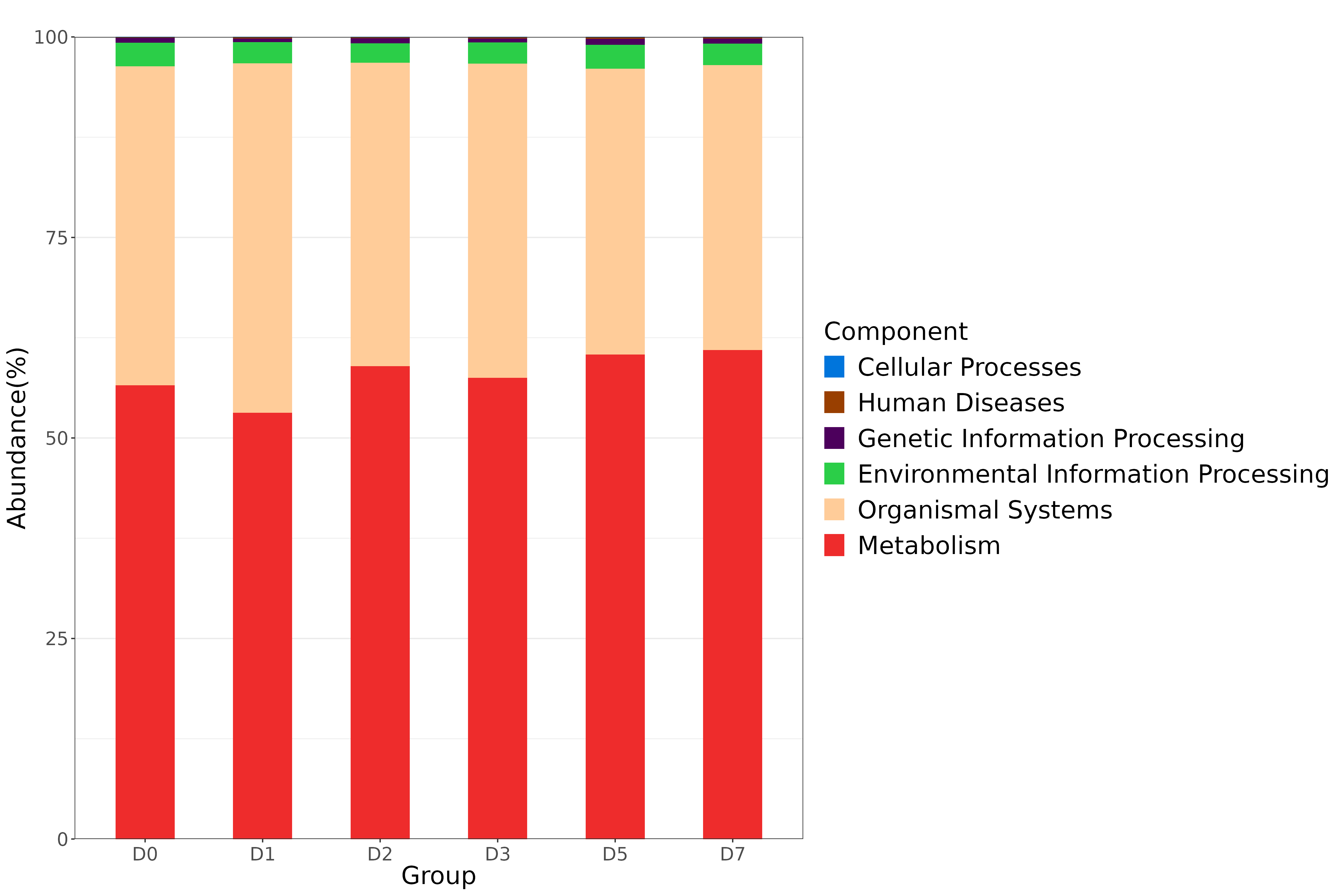

Supplement: Supplementary file 3 — Additional file 3. Raw data of the metabolomic compounds. [file 40104_2026_1385_MOESM3_ESM.zip › mix/KEGG_function_summary/Barplot/function_summary_level1_Group_barplot.png]

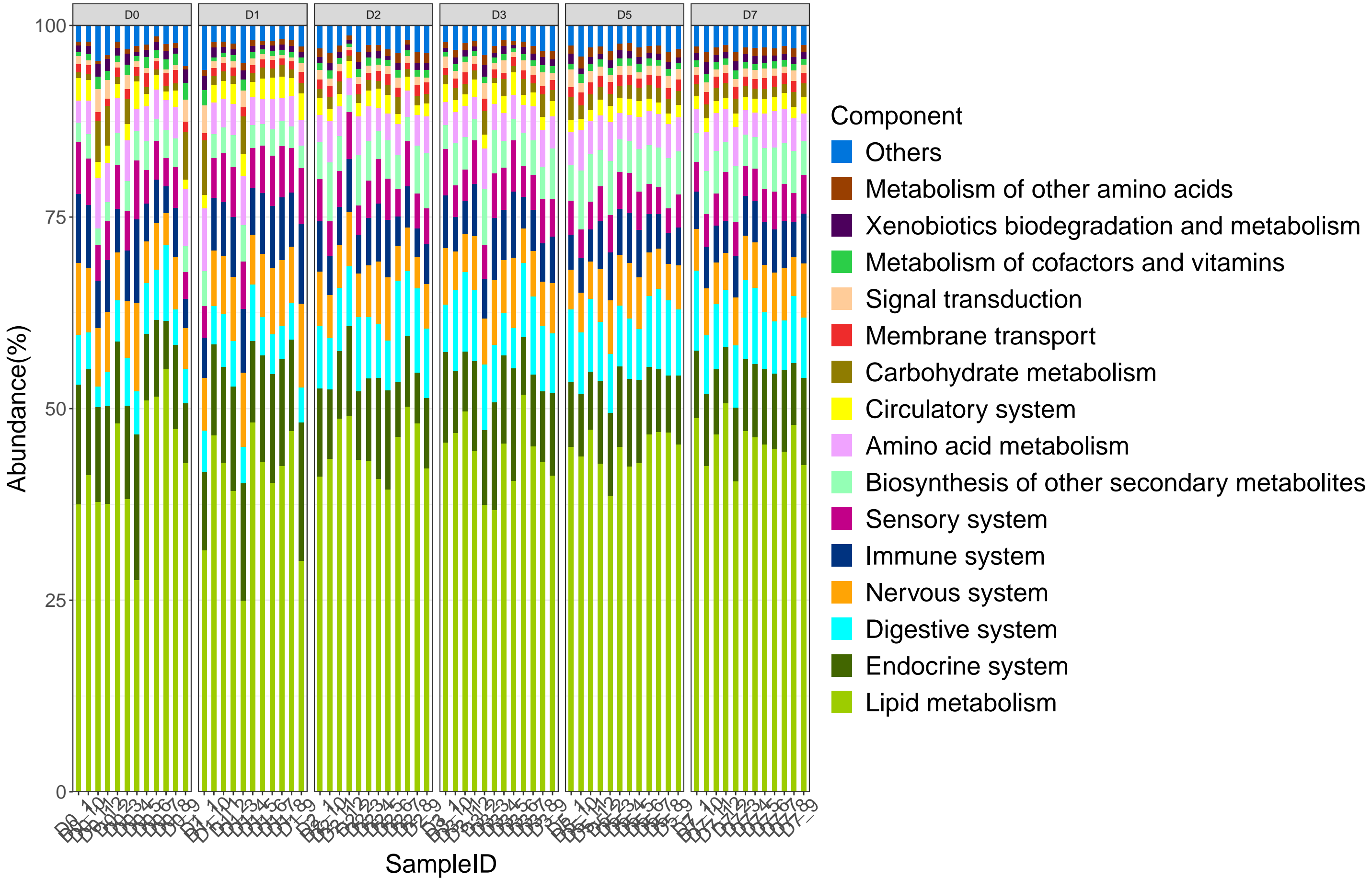

Supplement: Supplementary file 3 — Additional file 3. Raw data of the metabolomic compounds. [file 40104_2026_1385_MOESM3_ESM.zip › mix/KEGG_function_summary/Barplot/function_summary_level2_barplot.pdf]

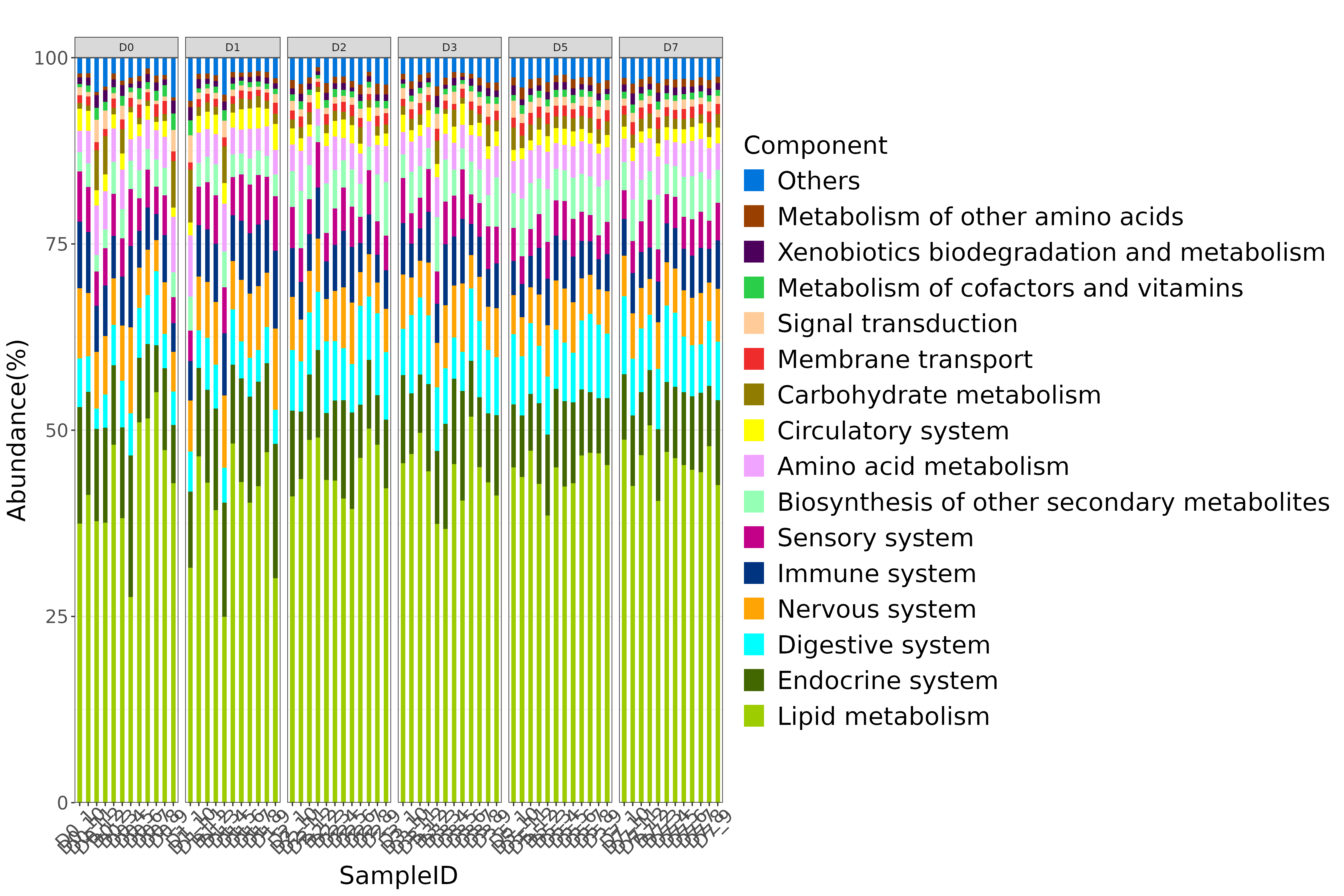

Supplement: Supplementary file 3 — Additional file 3. Raw data of the metabolomic compounds. [file 40104_2026_1385_MOESM3_ESM.zip › mix/KEGG_function_summary/Barplot/function_summary_level2_barplot.png]

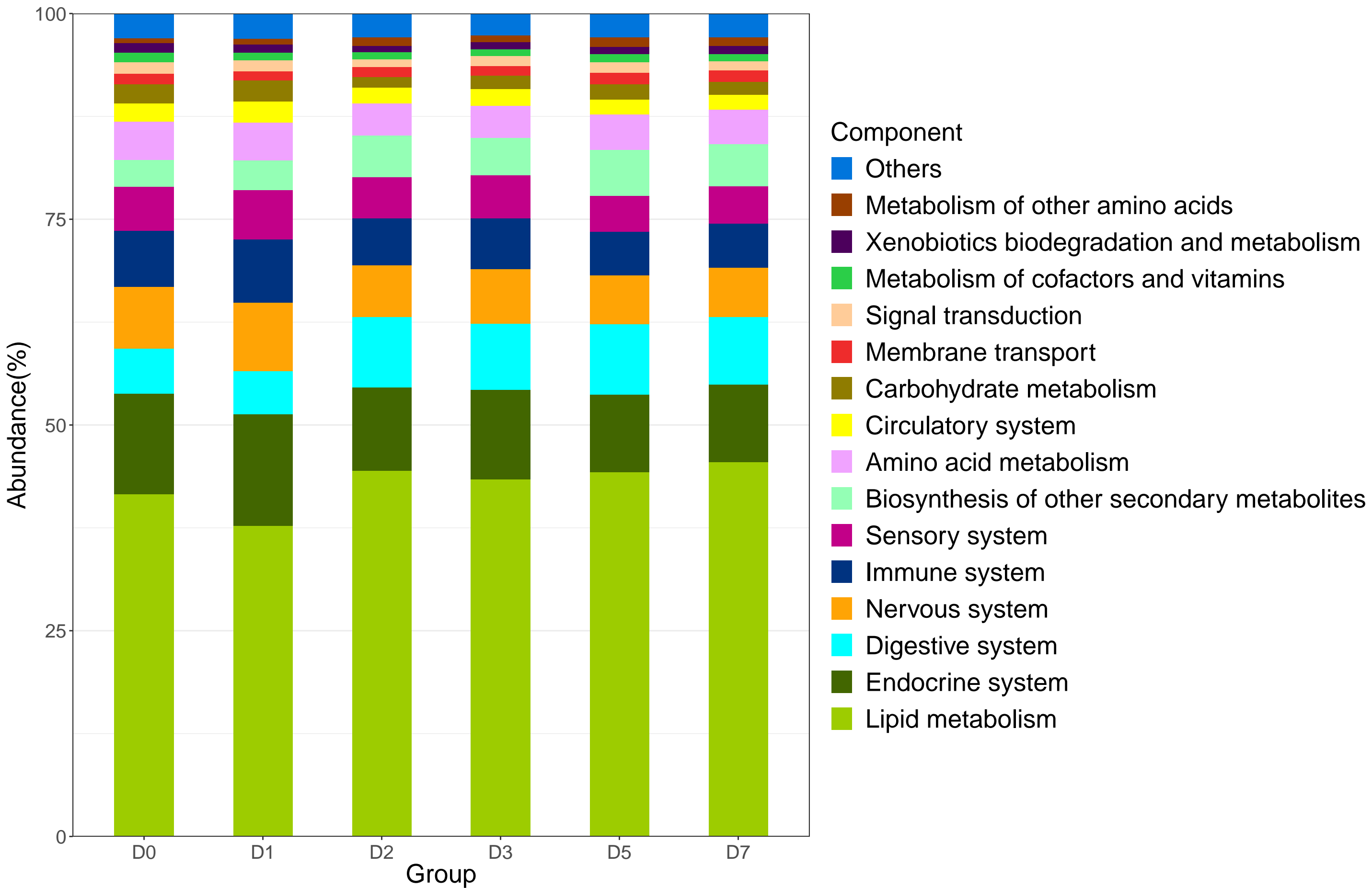

Supplement: Supplementary file 3 — Additional file 3. Raw data of the metabolomic compounds. [file 40104_2026_1385_MOESM3_ESM.zip › mix/KEGG_function_summary/Barplot/function_summary_level2_Group_barplot.pdf]

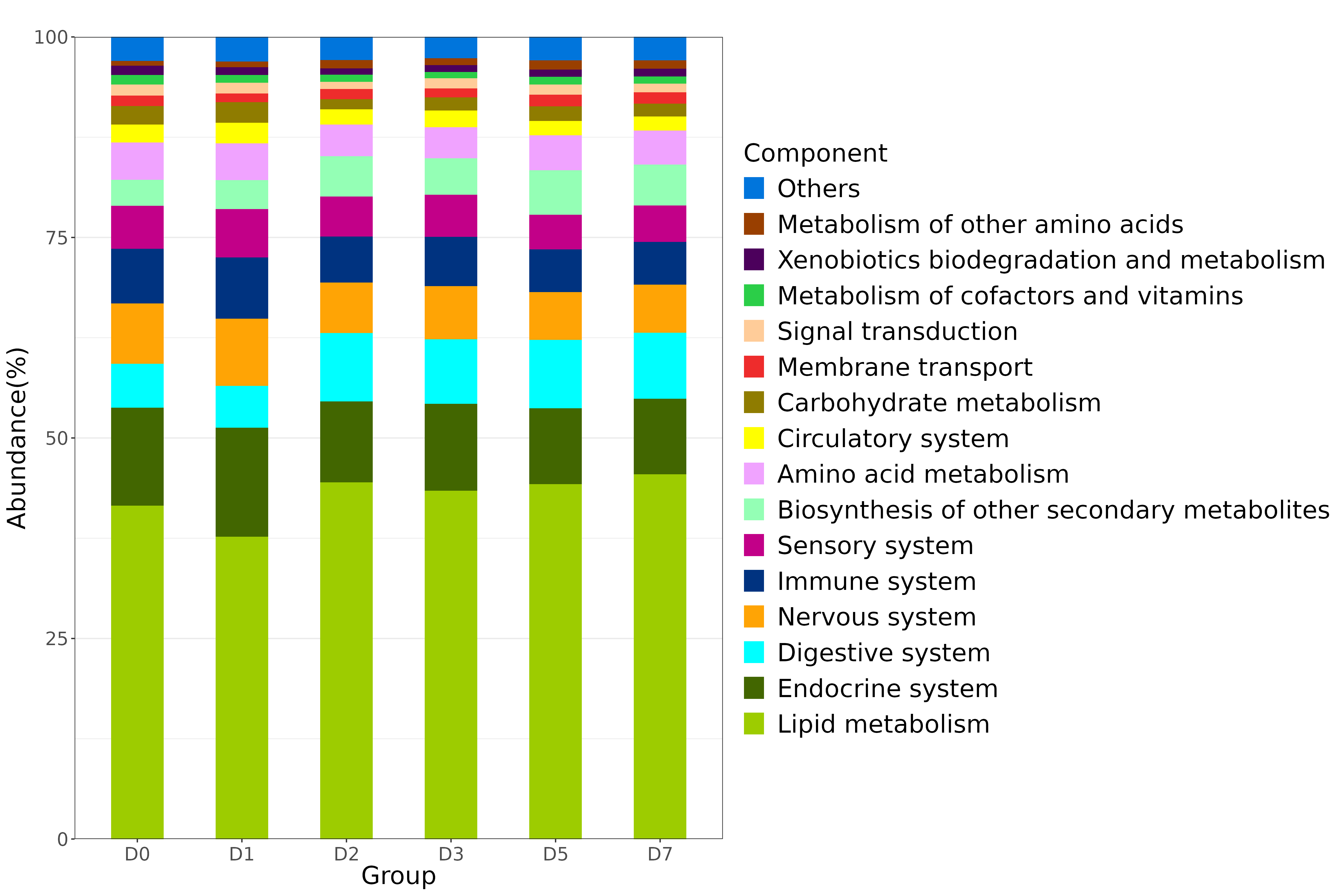

Supplement: Supplementary file 3 — Additional file 3. Raw data of the metabolomic compounds. [file 40104_2026_1385_MOESM3_ESM.zip › mix/KEGG_function_summary/Barplot/function_summary_level2_Group_barplot.png]

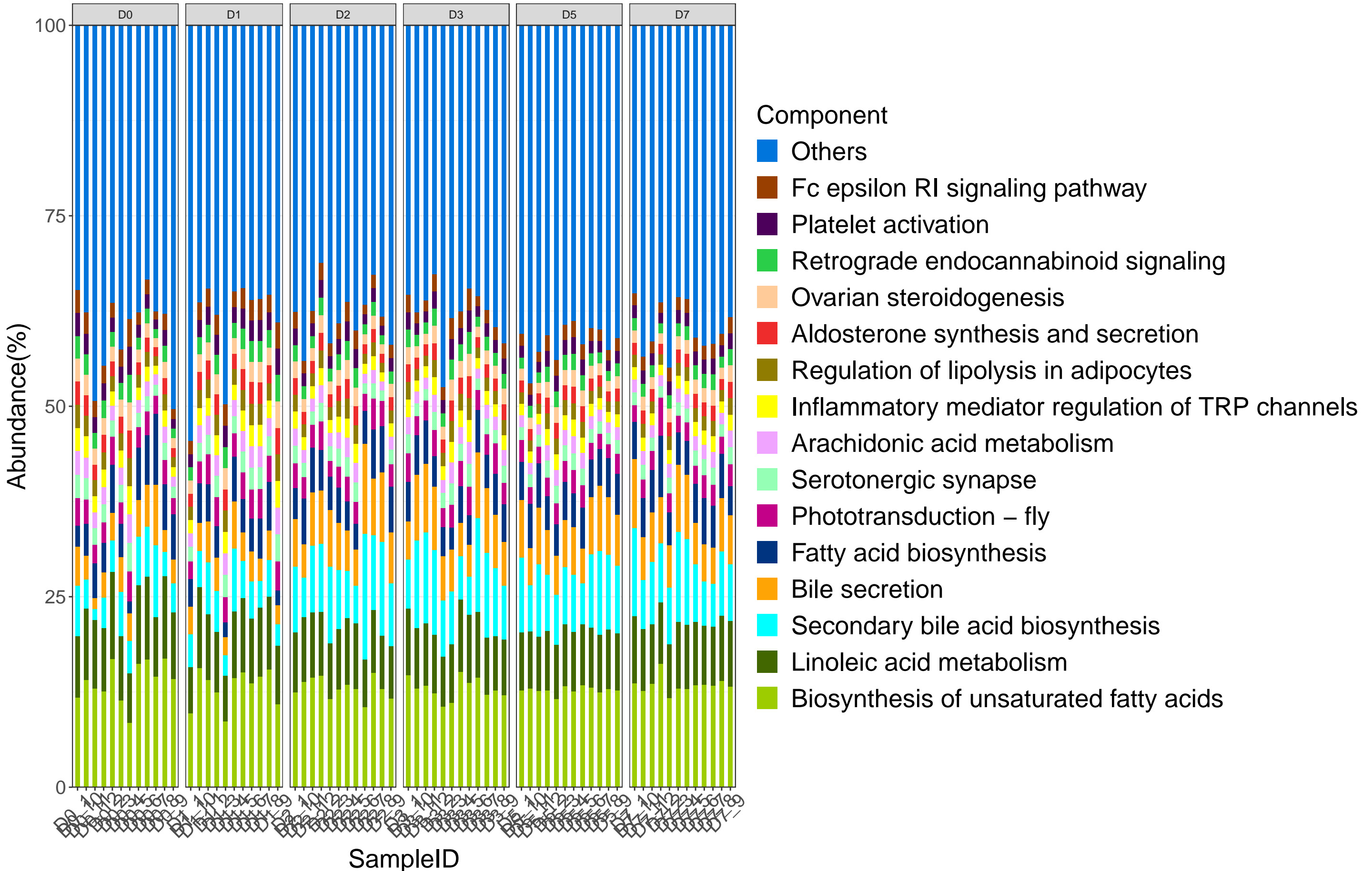

Supplement: Supplementary file 3 — Additional file 3. Raw data of the metabolomic compounds. [file 40104_2026_1385_MOESM3_ESM.zip › mix/KEGG_function_summary/Barplot/function_summary_level3_barplot.pdf]

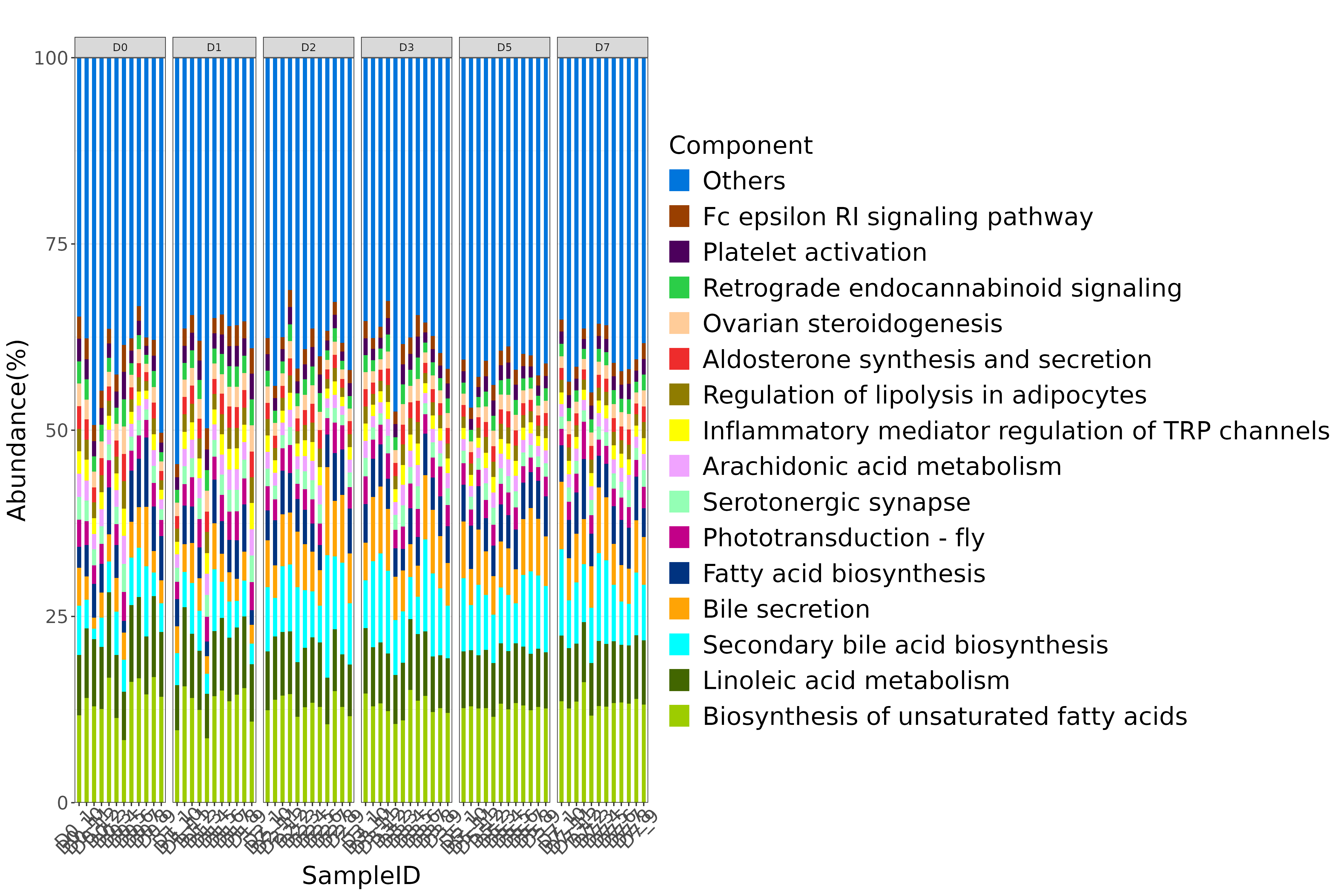

Supplement: Supplementary file 3 — Additional file 3. Raw data of the metabolomic compounds. [file 40104_2026_1385_MOESM3_ESM.zip › mix/KEGG_function_summary/Barplot/function_summary_level3_barplot.png]

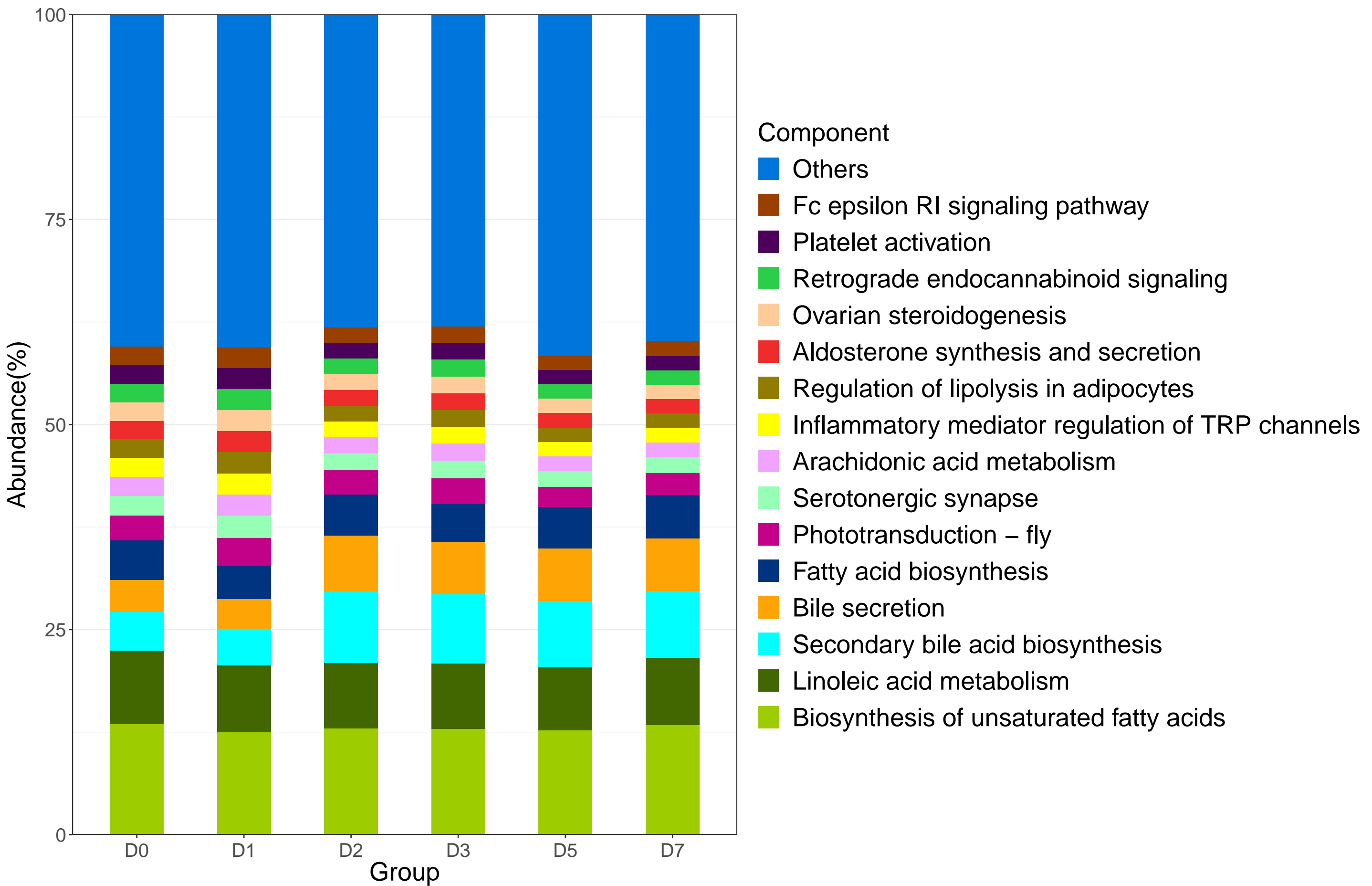

Supplement: Supplementary file 3 — Additional file 3. Raw data of the metabolomic compounds. [file 40104_2026_1385_MOESM3_ESM.zip › mix/KEGG_function_summary/Barplot/function_summary_level3_Group_barplot.pdf]

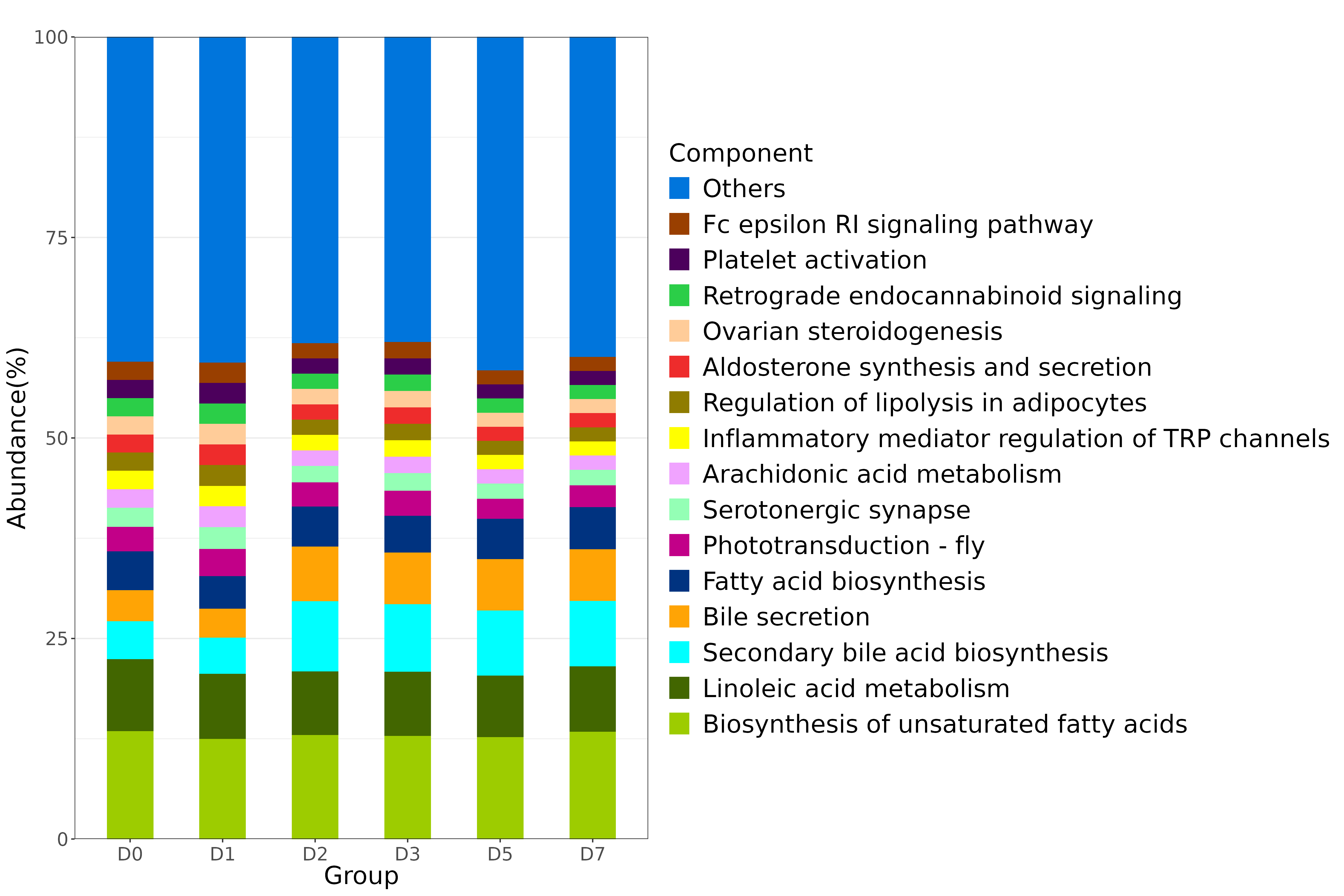

Supplement: Supplementary file 3 — Additional file 3. Raw data of the metabolomic compounds. [file 40104_2026_1385_MOESM3_ESM.zip › mix/KEGG_function_summary/Barplot/function_summary_level3_Group_barplot.png]

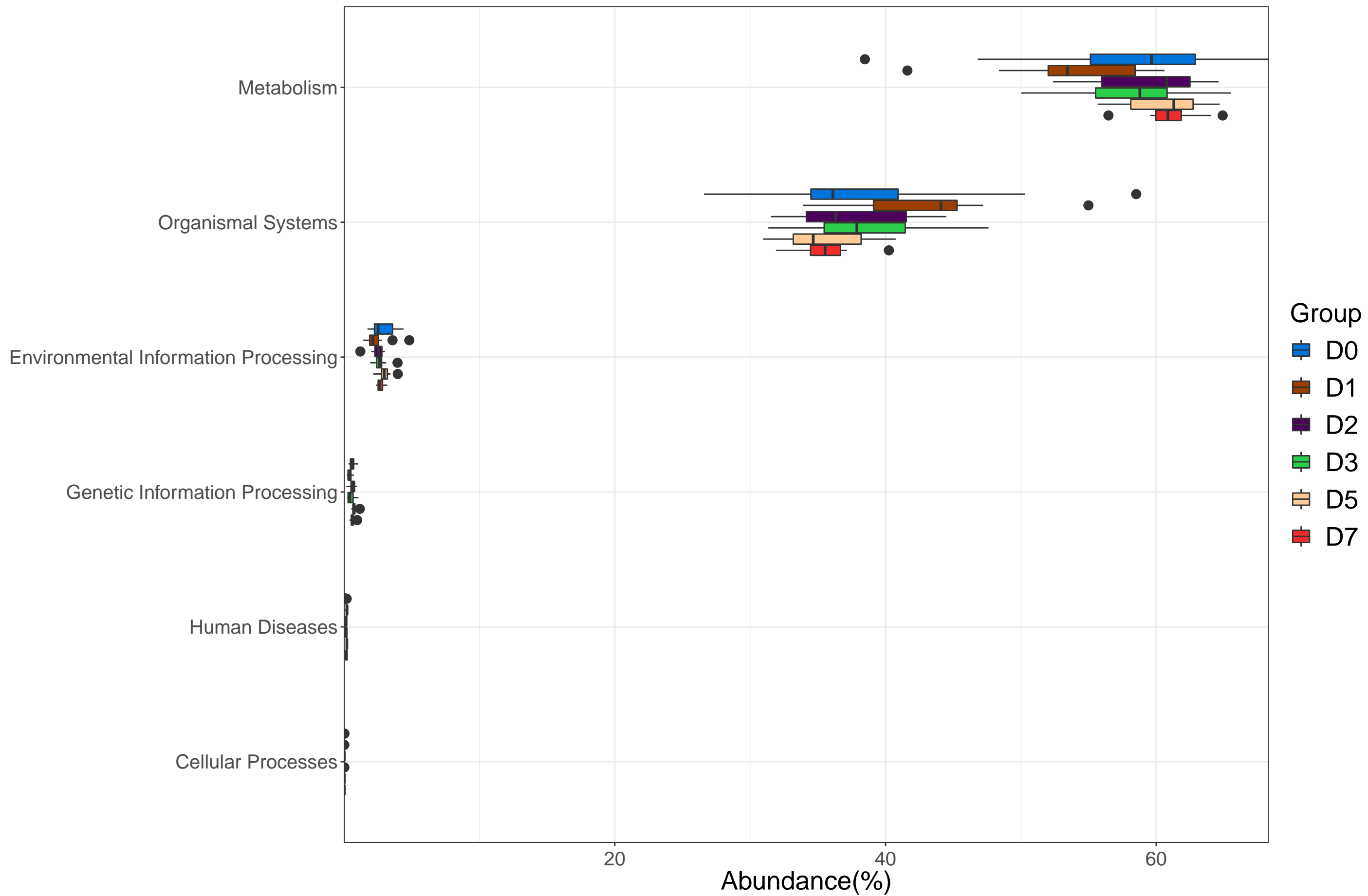

Supplement: Supplementary file 3 — Additional file 3. Raw data of the metabolomic compounds. [file 40104_2026_1385_MOESM3_ESM.zip › mix/KEGG_function_summary/Boxplot/function_summary_level1_boxplot.pdf]

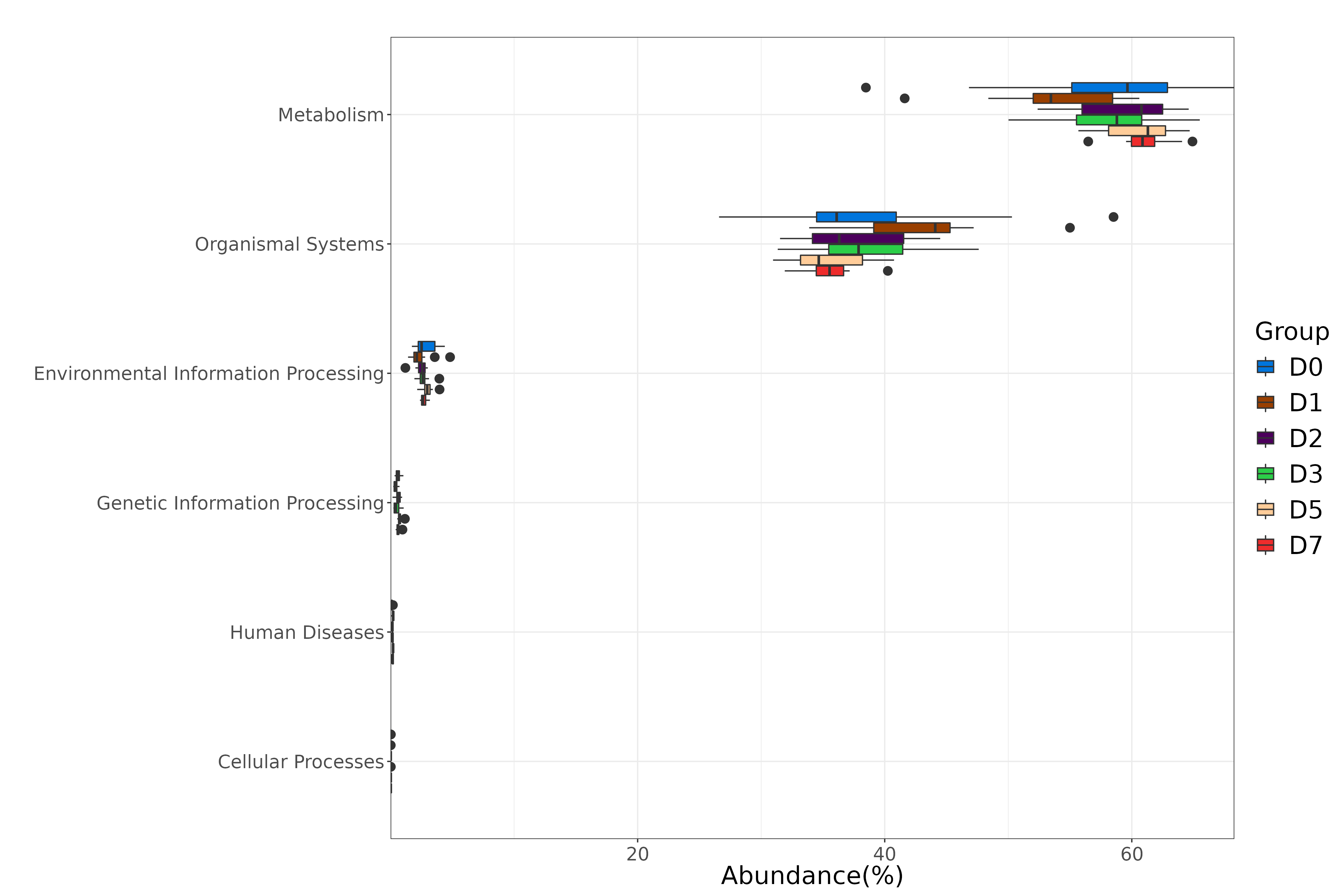

Supplement: Supplementary file 3 — Additional file 3. Raw data of the metabolomic compounds. [file 40104_2026_1385_MOESM3_ESM.zip › mix/KEGG_function_summary/Boxplot/function_summary_level1_boxplot.png]

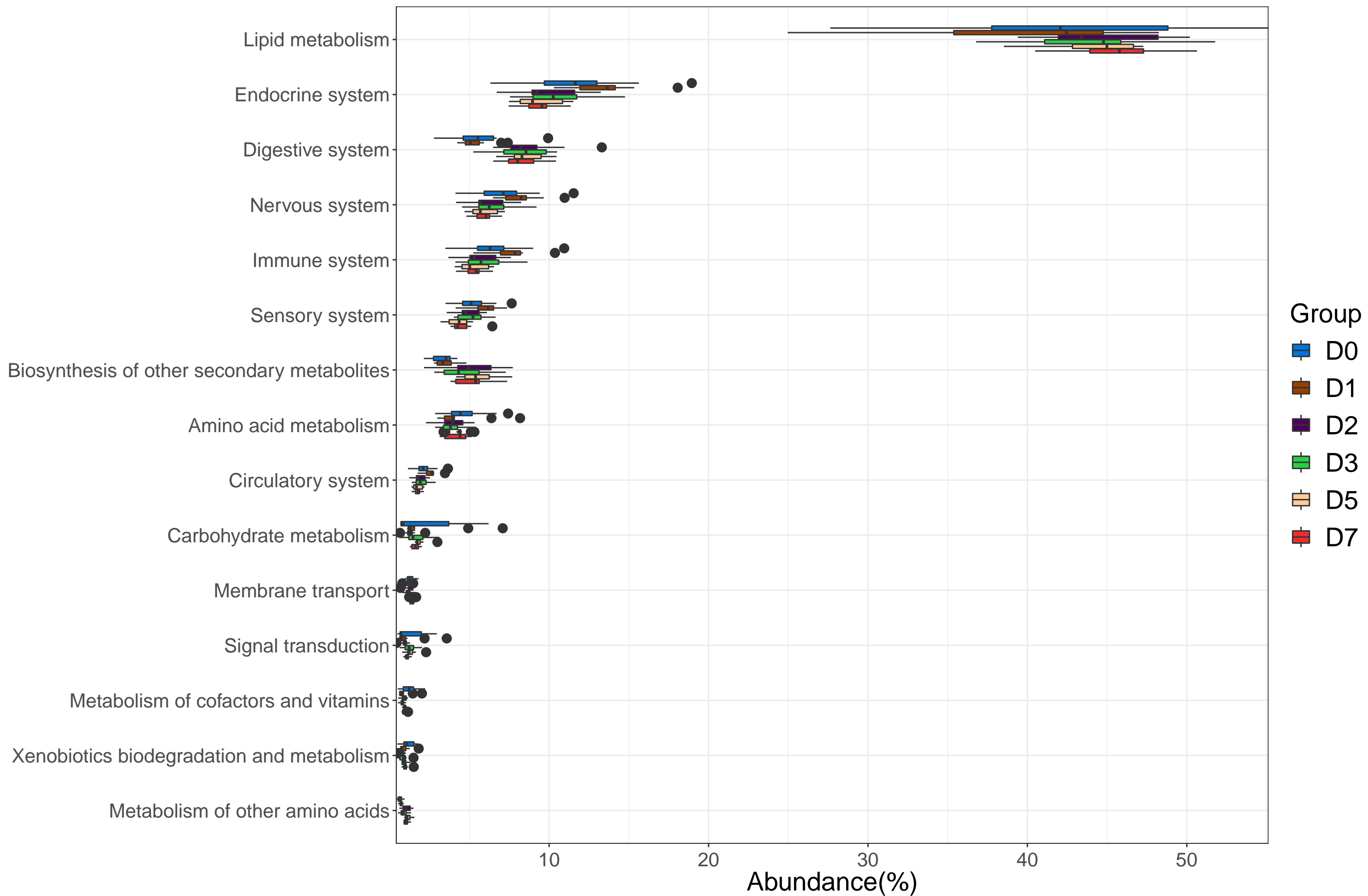

Supplement: Supplementary file 3 — Additional file 3. Raw data of the metabolomic compounds. [file 40104_2026_1385_MOESM3_ESM.zip › mix/KEGG_function_summary/Boxplot/function_summary_level2_boxplot.pdf]

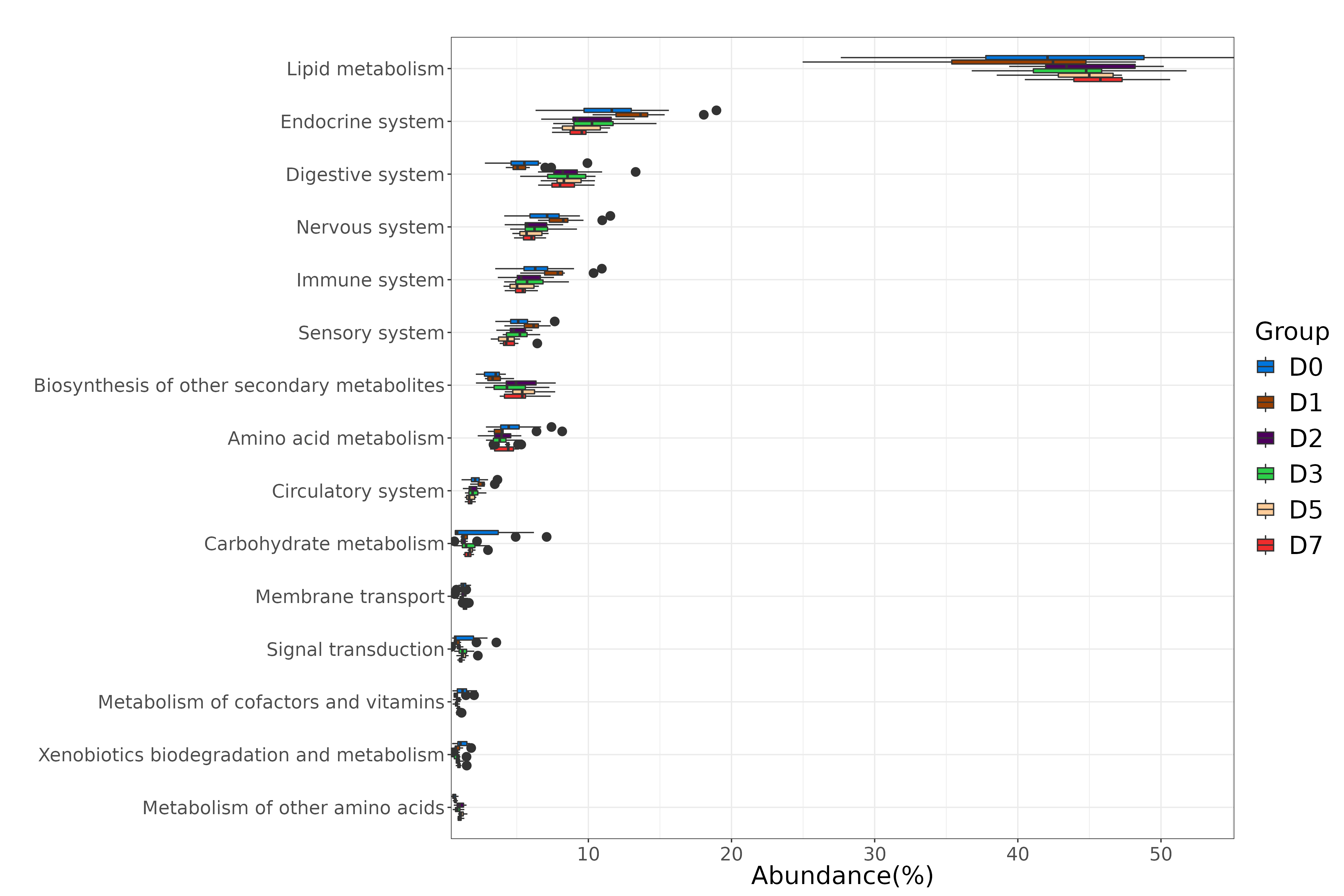

Supplement: Supplementary file 3 — Additional file 3. Raw data of the metabolomic compounds. [file 40104_2026_1385_MOESM3_ESM.zip › mix/KEGG_function_summary/Boxplot/function_summary_level2_boxplot.png]

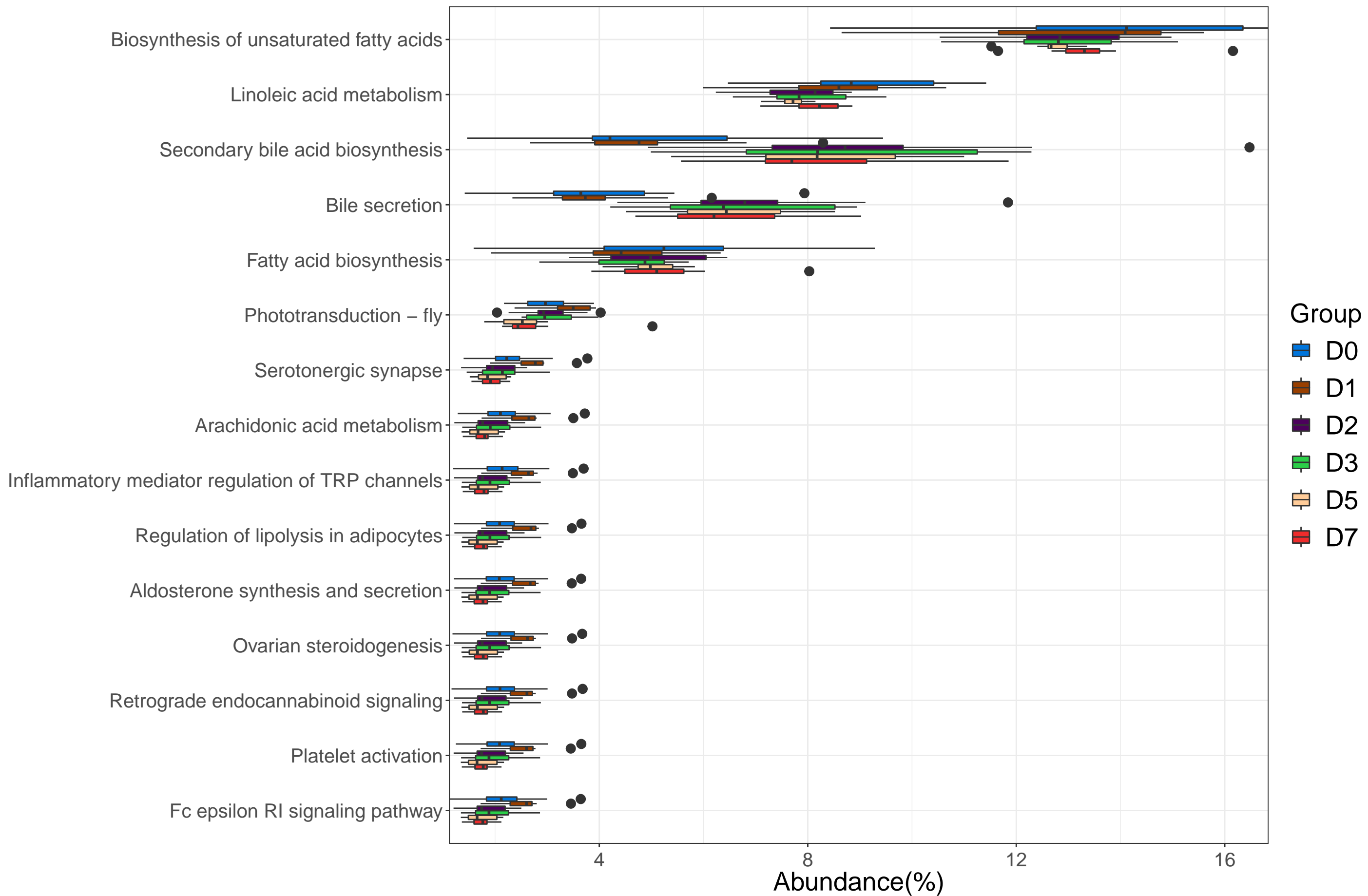

Supplement: Supplementary file 3 — Additional file 3. Raw data of the metabolomic compounds. [file 40104_2026_1385_MOESM3_ESM.zip › mix/KEGG_function_summary/Boxplot/function_summary_level3_boxplot.pdf]

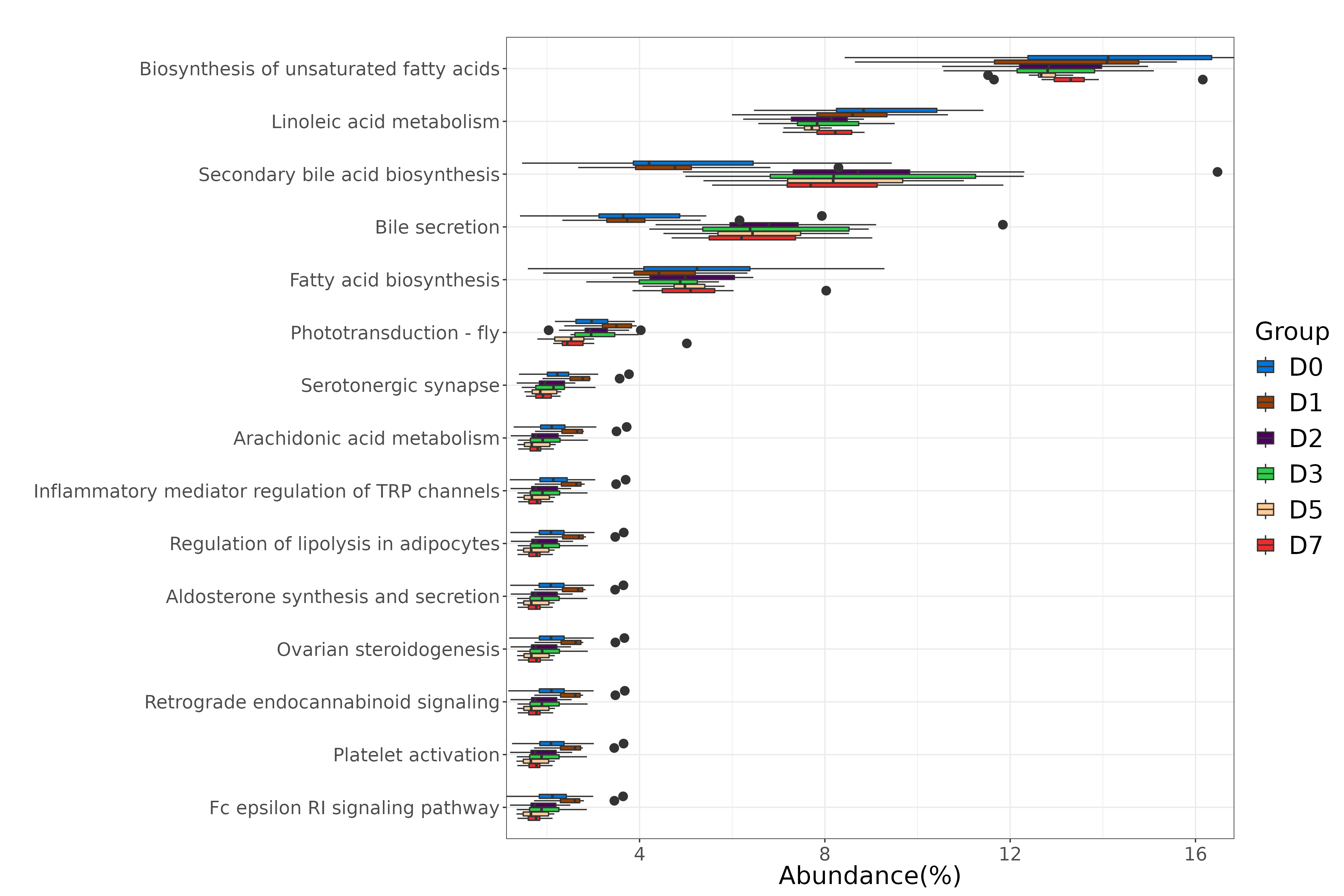

Supplement: Supplementary file 3 — Additional file 3. Raw data of the metabolomic compounds. [file 40104_2026_1385_MOESM3_ESM.zip › mix/KEGG_function_summary/Boxplot/function_summary_level3_boxplot.png]

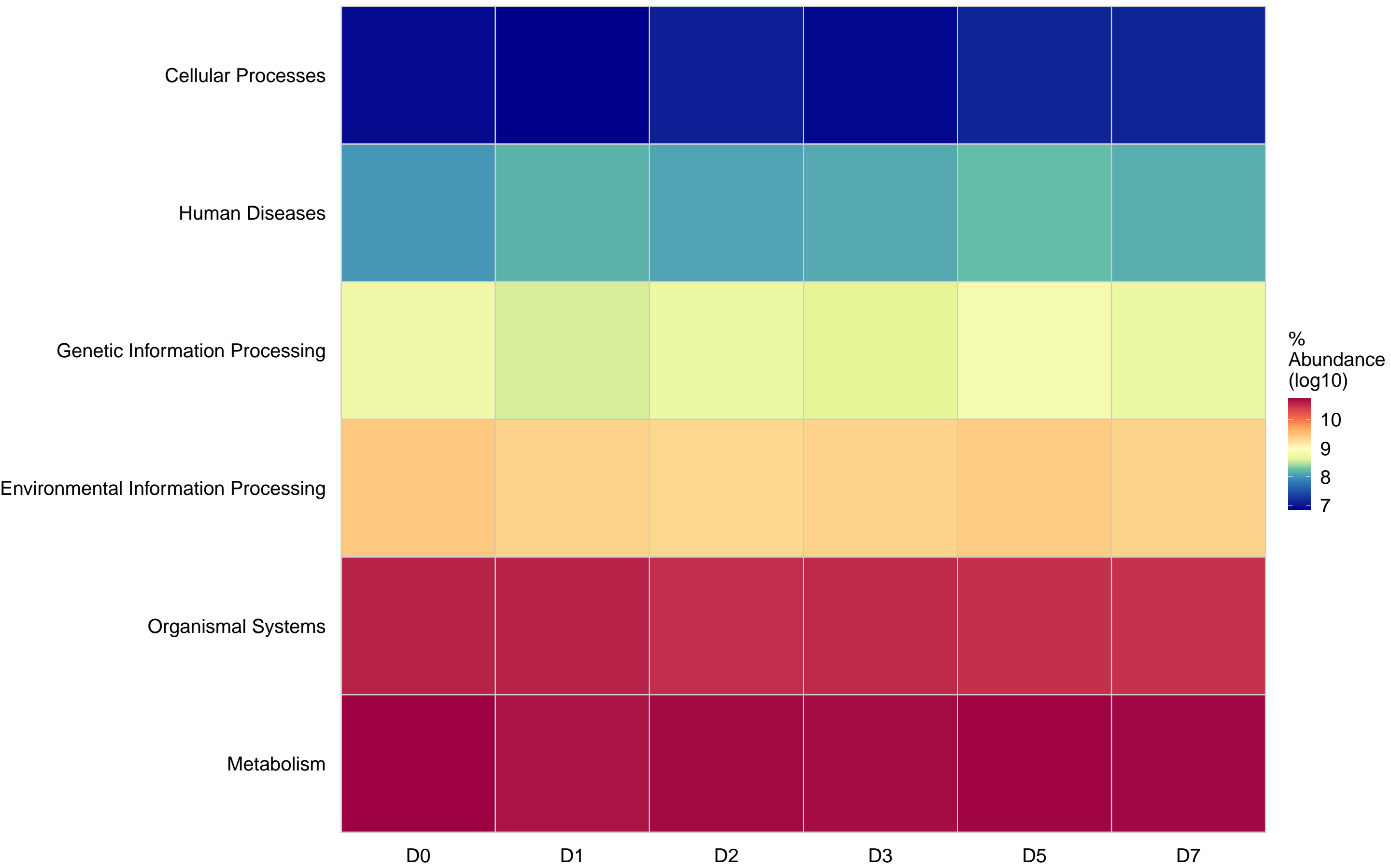

Supplement: Supplementary file 3 — Additional file 3. Raw data of the metabolomic compounds. [file 40104_2026_1385_MOESM3_ESM.zip › mix/KEGG_function_summary/Heatmap/function_summary_level1_Group_heatmap.pdf]

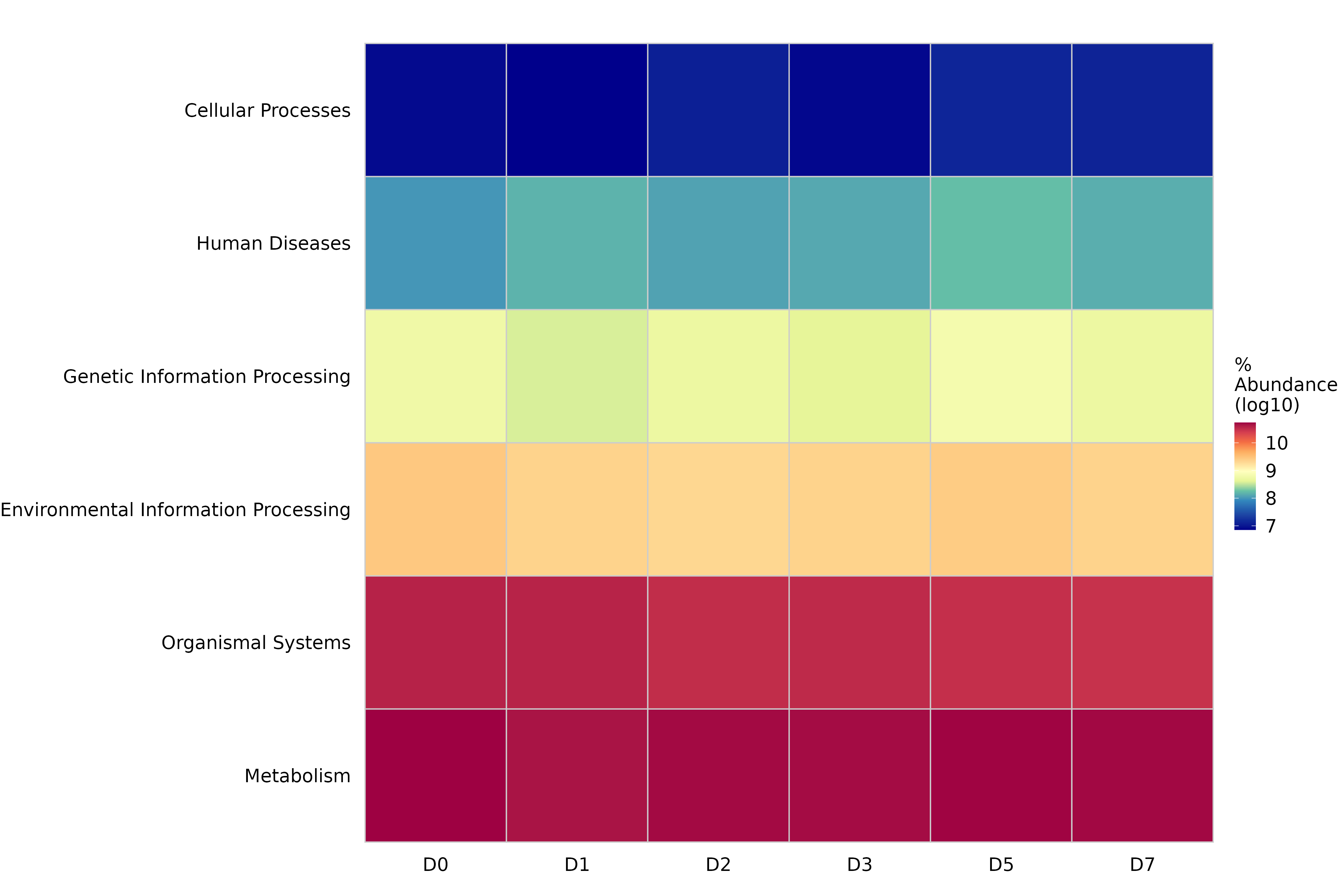

Supplement: Supplementary file 3 — Additional file 3. Raw data of the metabolomic compounds. [file 40104_2026_1385_MOESM3_ESM.zip › mix/KEGG_function_summary/Heatmap/function_summary_level1_Group_heatmap.png]

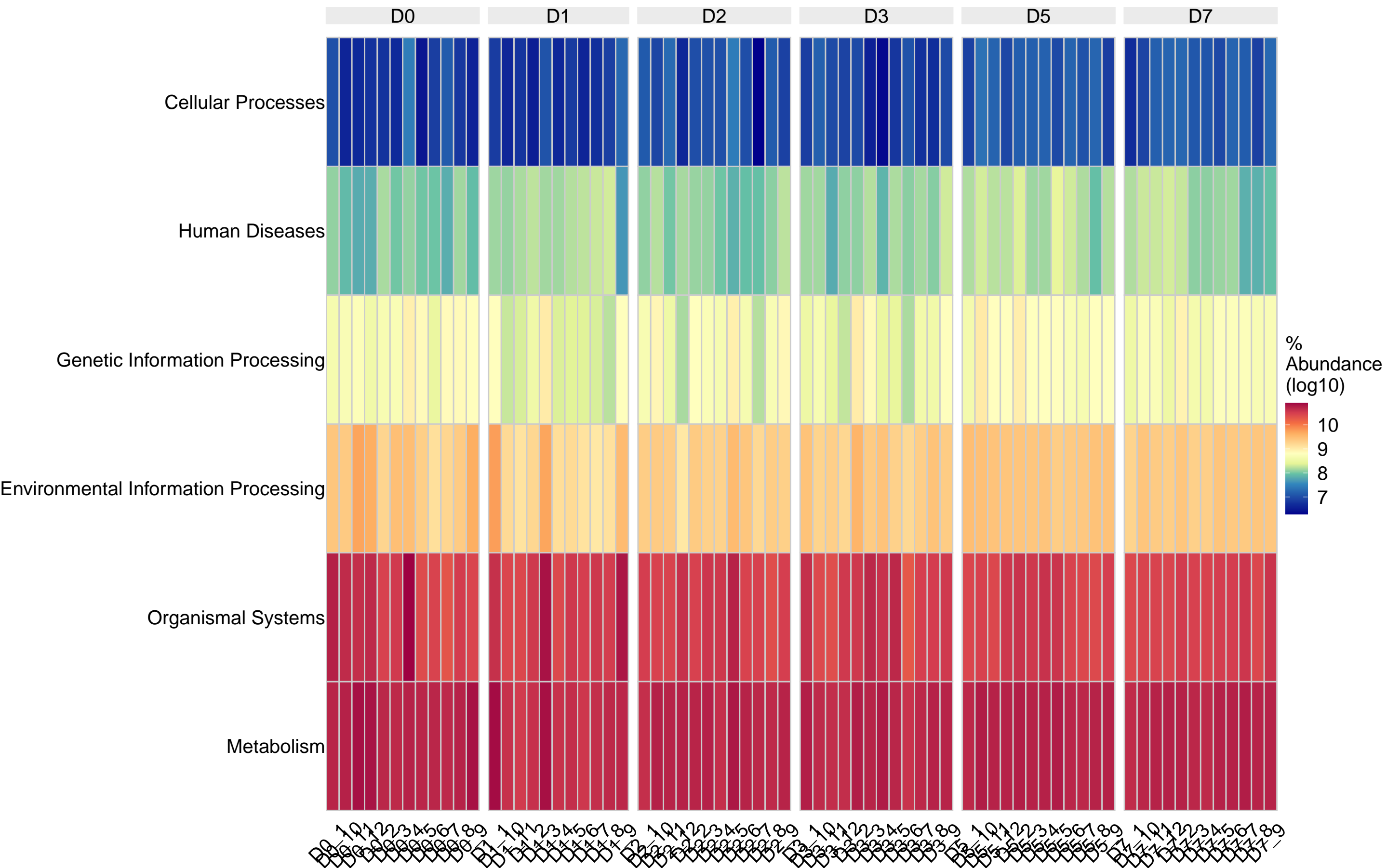

Supplement: Supplementary file 3 — Additional file 3. Raw data of the metabolomic compounds. [file 40104_2026_1385_MOESM3_ESM.zip › mix/KEGG_function_summary/Heatmap/function_summary_level1_heatmap.pdf]

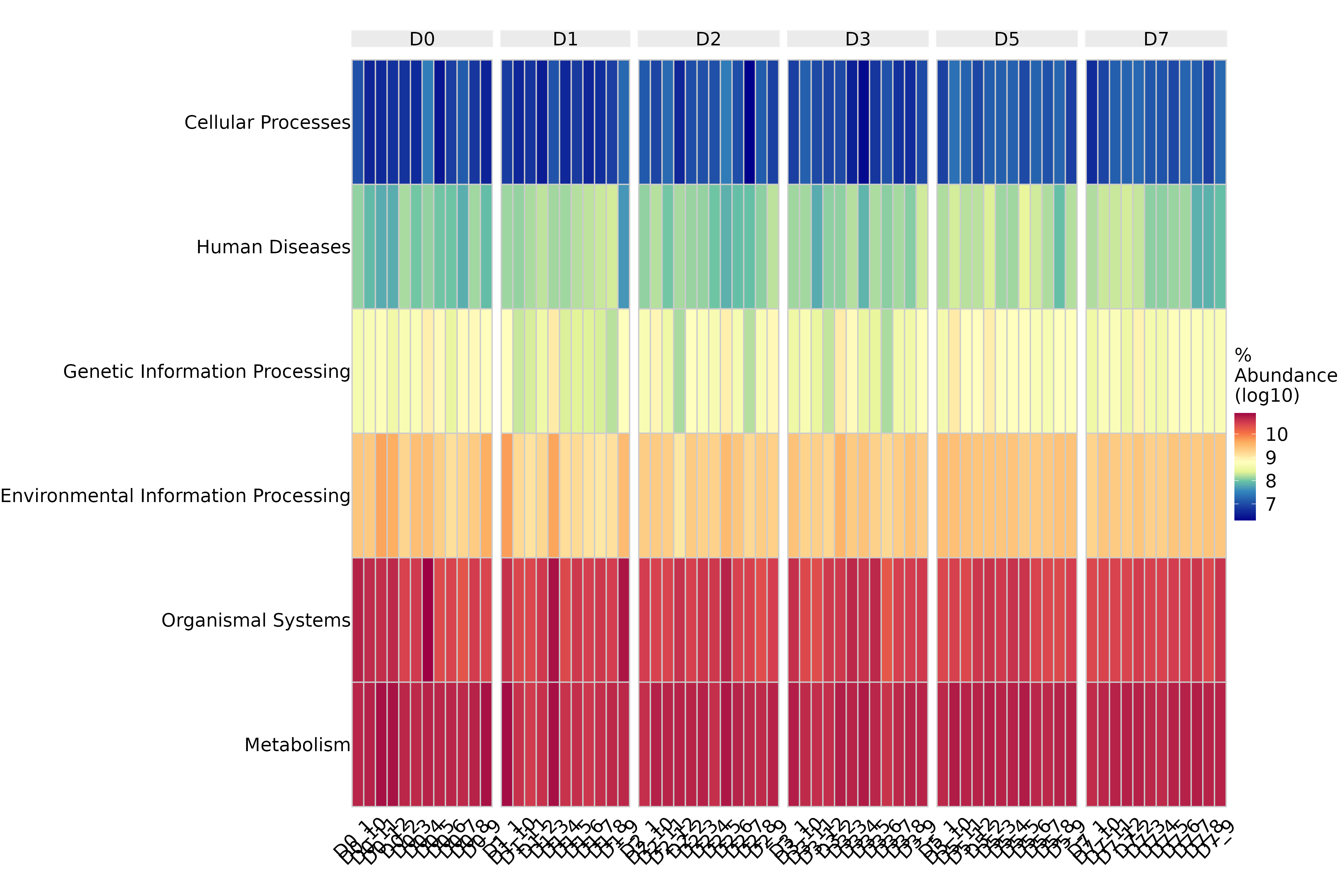

Supplement: Supplementary file 3 — Additional file 3. Raw data of the metabolomic compounds. [file 40104_2026_1385_MOESM3_ESM.zip › mix/KEGG_function_summary/Heatmap/function_summary_level1_heatmap.png]

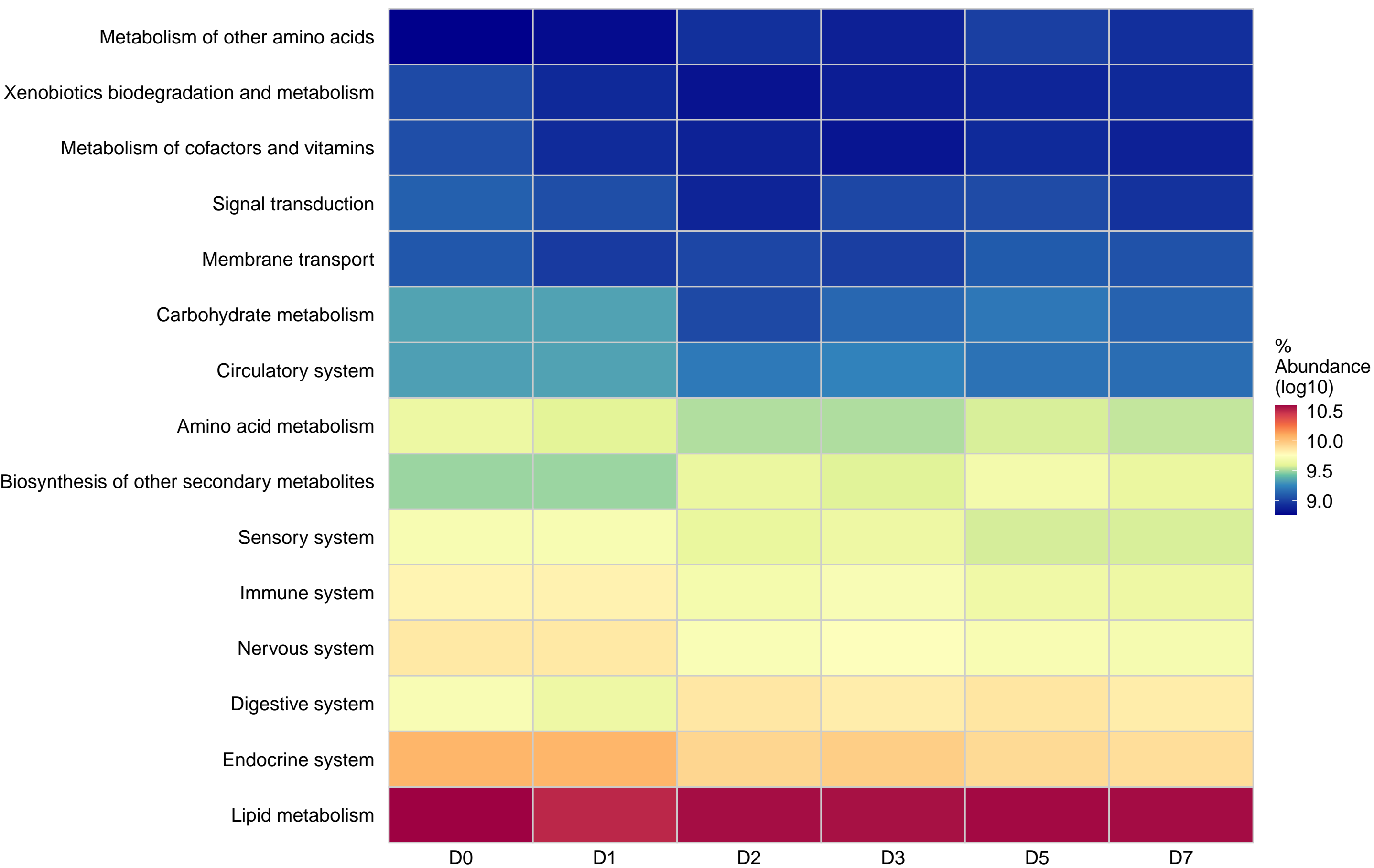

Supplement: Supplementary file 3 — Additional file 3. Raw data of the metabolomic compounds. [file 40104_2026_1385_MOESM3_ESM.zip › mix/KEGG_function_summary/Heatmap/function_summary_level2_Group_heatmap.pdf]

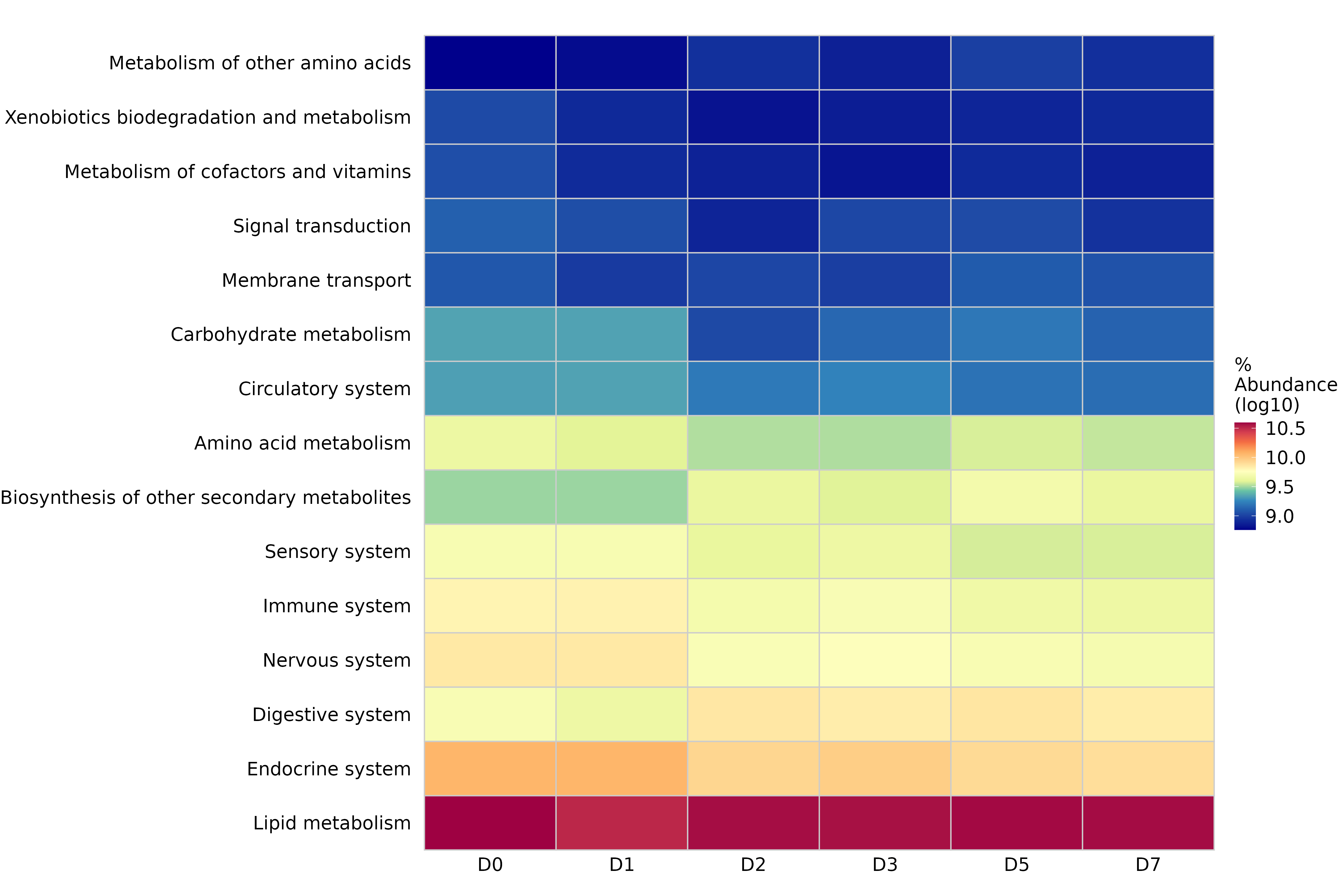

Supplement: Supplementary file 3 — Additional file 3. Raw data of the metabolomic compounds. [file 40104_2026_1385_MOESM3_ESM.zip › mix/KEGG_function_summary/Heatmap/function_summary_level2_Group_heatmap.png]

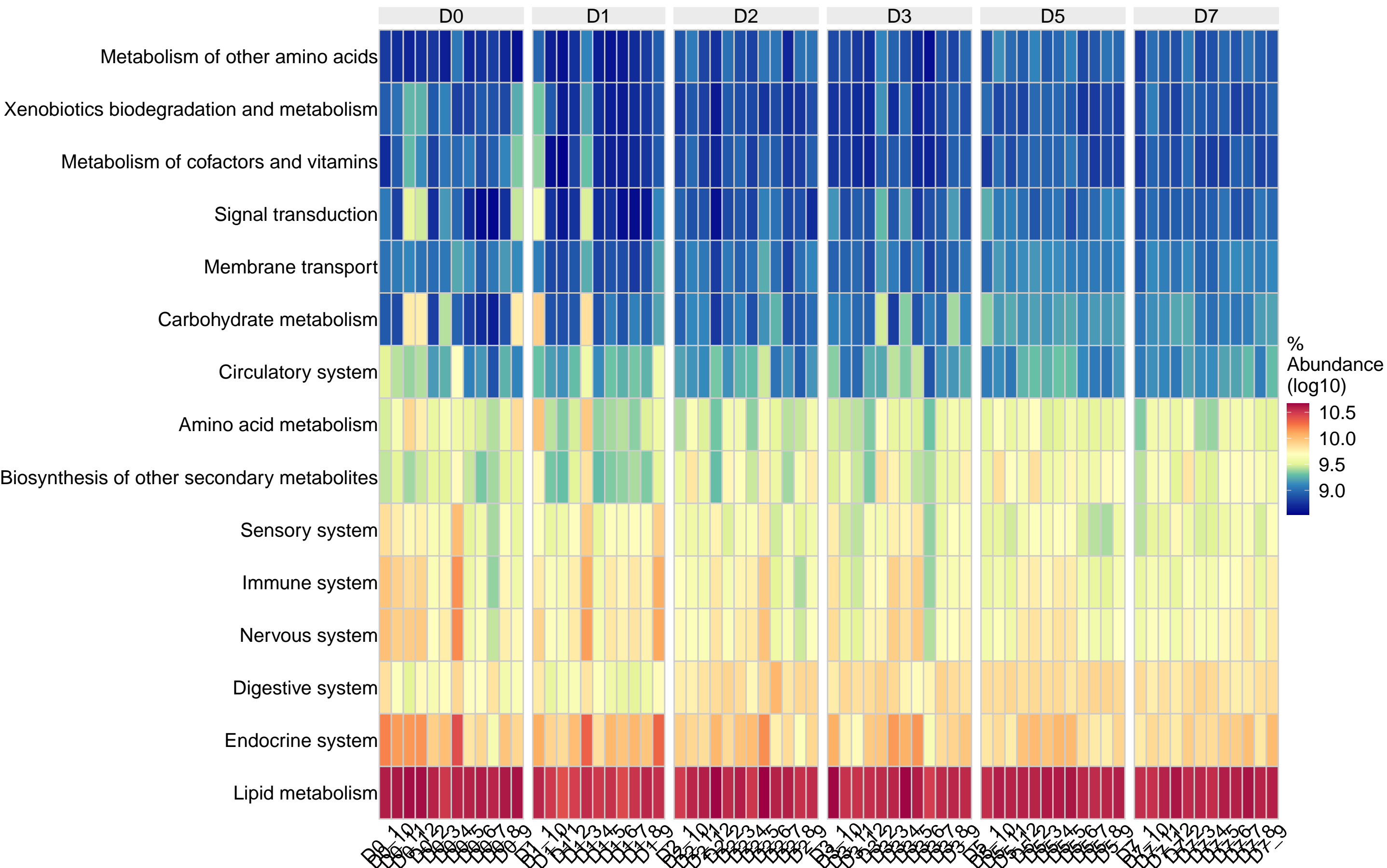

Supplement: Supplementary file 3 — Additional file 3. Raw data of the metabolomic compounds. [file 40104_2026_1385_MOESM3_ESM.zip › mix/KEGG_function_summary/Heatmap/function_summary_level2_heatmap.pdf]

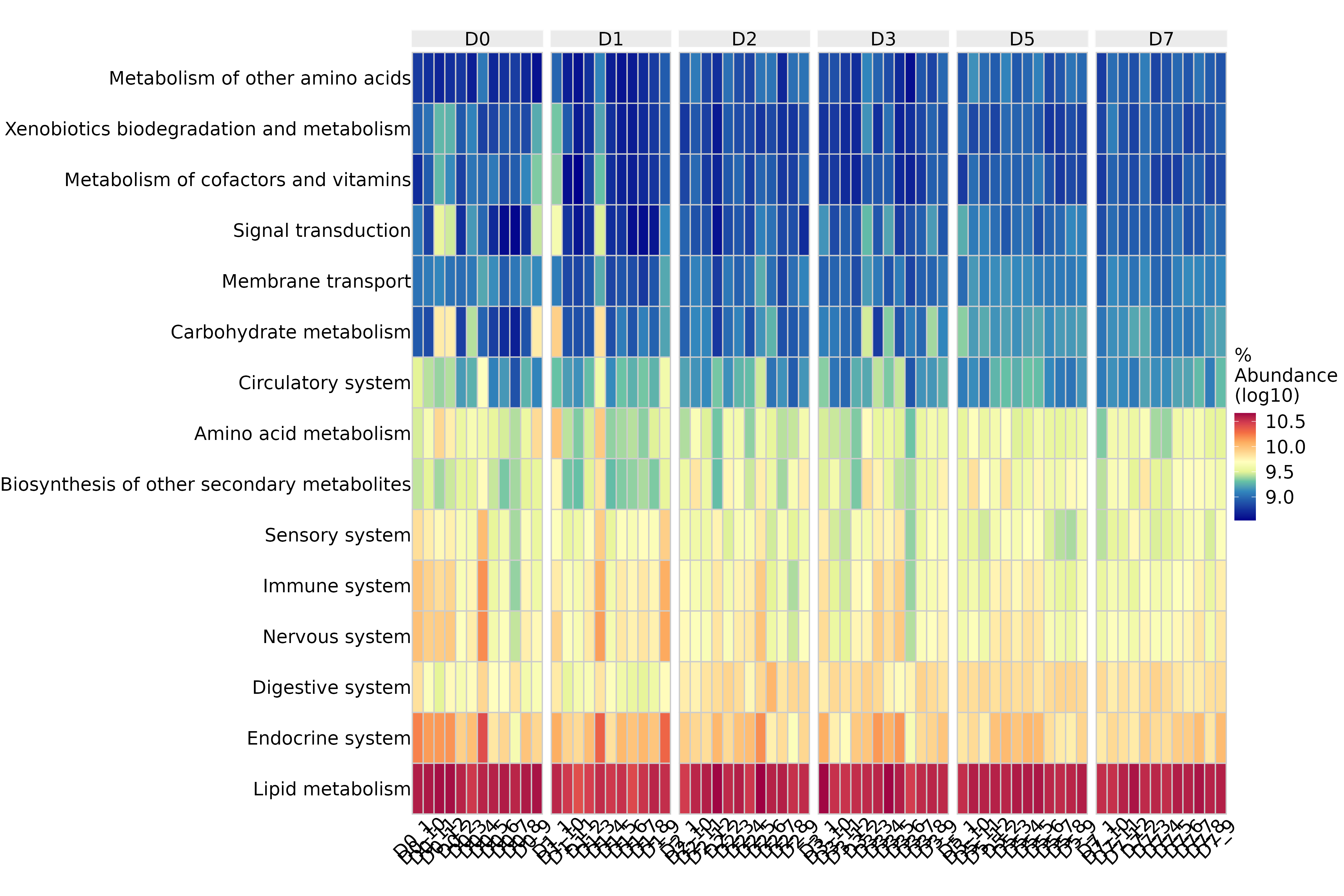

Supplement: Supplementary file 3 — Additional file 3. Raw data of the metabolomic compounds. [file 40104_2026_1385_MOESM3_ESM.zip › mix/KEGG_function_summary/Heatmap/function_summary_level2_heatmap.png]

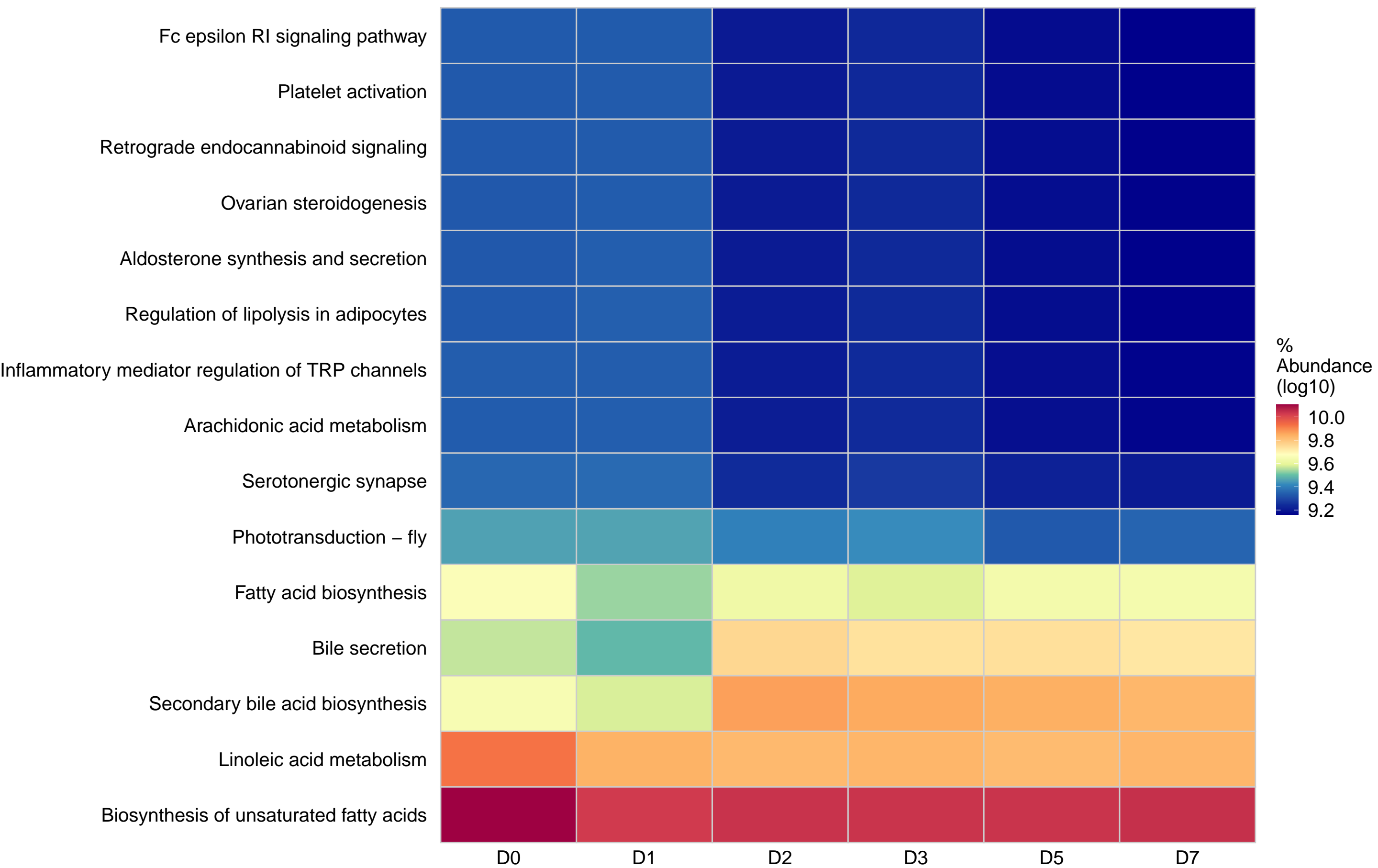

Supplement: Supplementary file 3 — Additional file 3. Raw data of the metabolomic compounds. [file 40104_2026_1385_MOESM3_ESM.zip › mix/KEGG_function_summary/Heatmap/function_summary_level3_Group_heatmap.pdf]

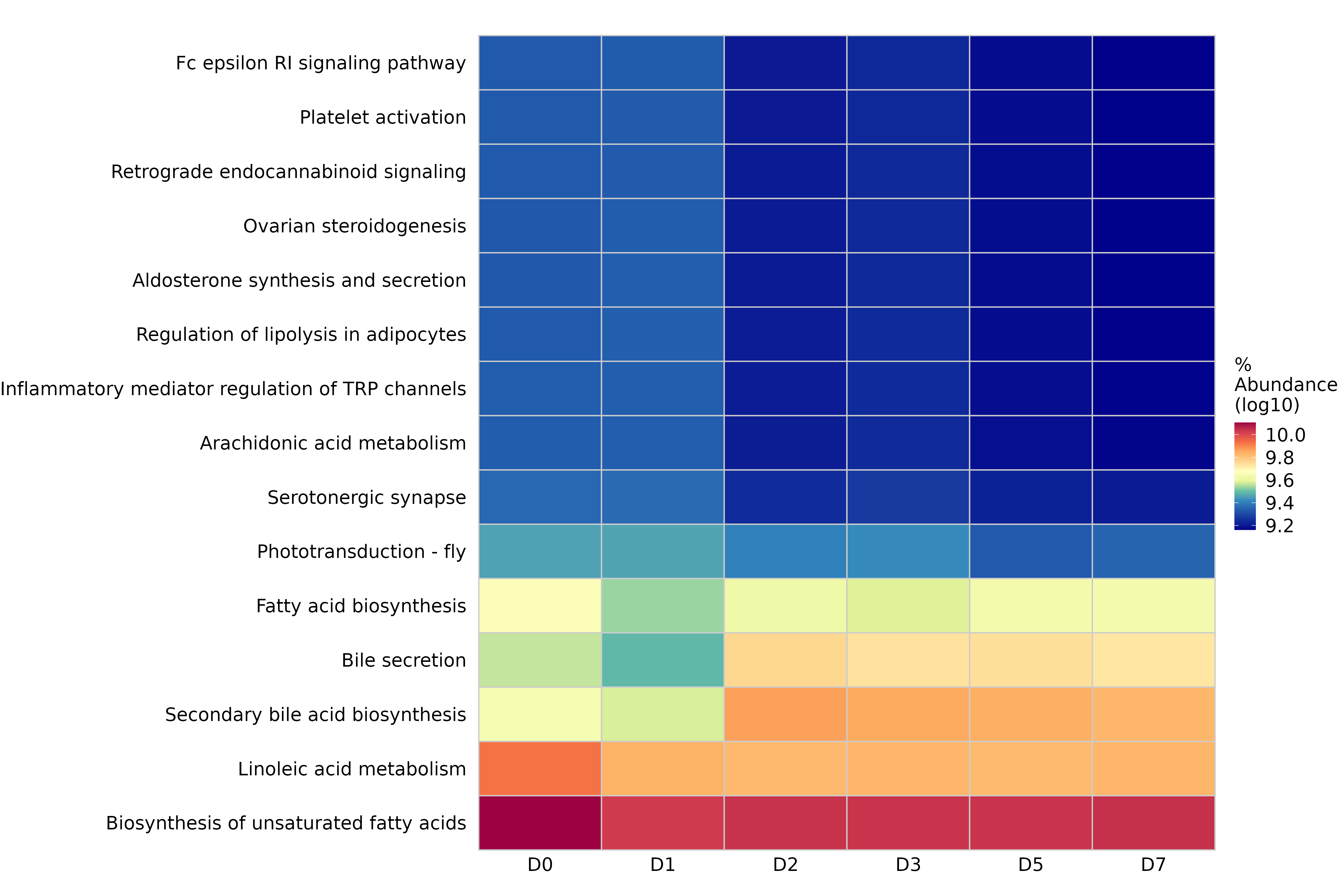

Supplement: Supplementary file 3 — Additional file 3. Raw data of the metabolomic compounds. [file 40104_2026_1385_MOESM3_ESM.zip › mix/KEGG_function_summary/Heatmap/function_summary_level3_Group_heatmap.png]

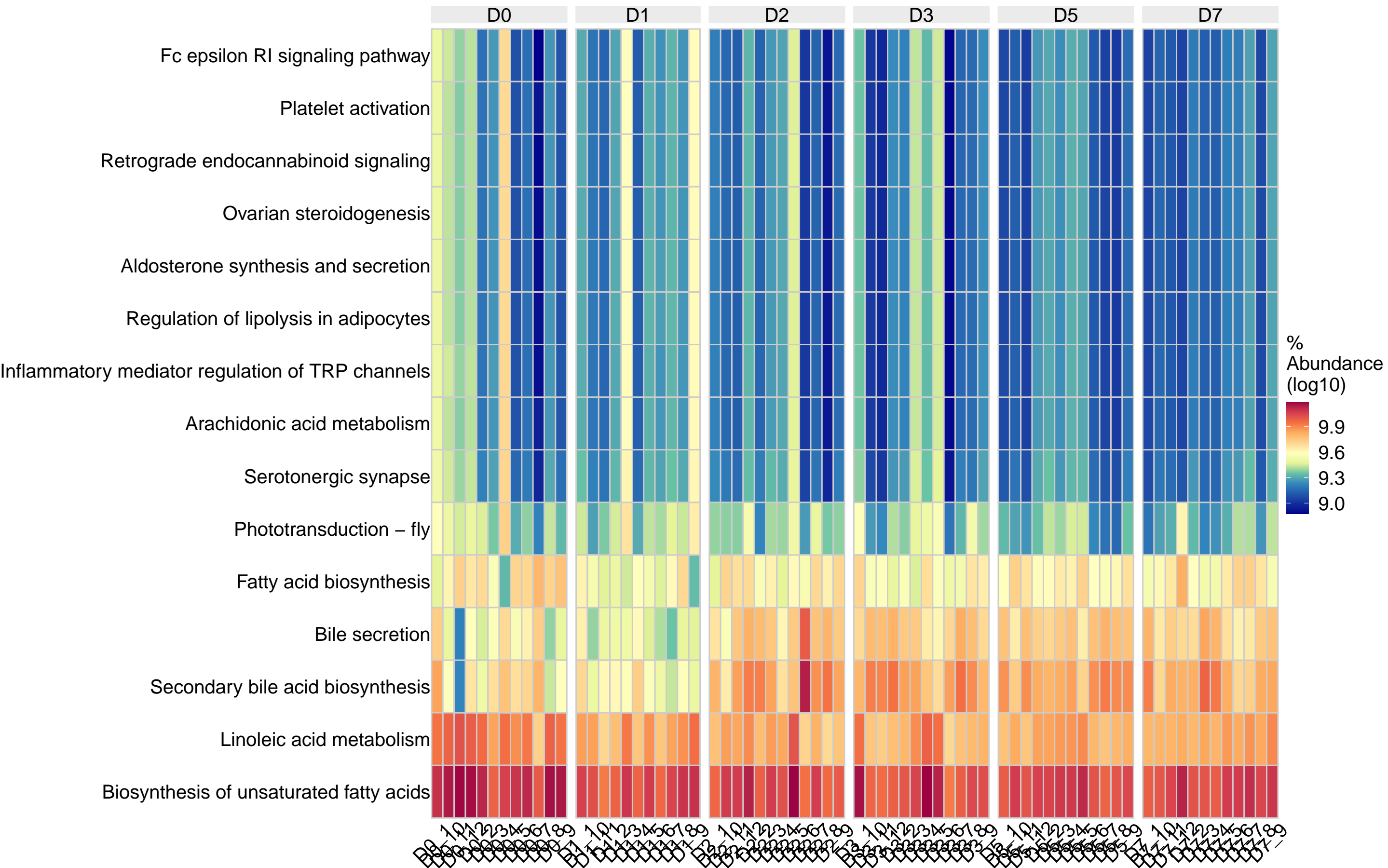

Supplement: Supplementary file 3 — Additional file 3. Raw data of the metabolomic compounds. [file 40104_2026_1385_MOESM3_ESM.zip › mix/KEGG_function_summary/Heatmap/function_summary_level3_heatmap.pdf]

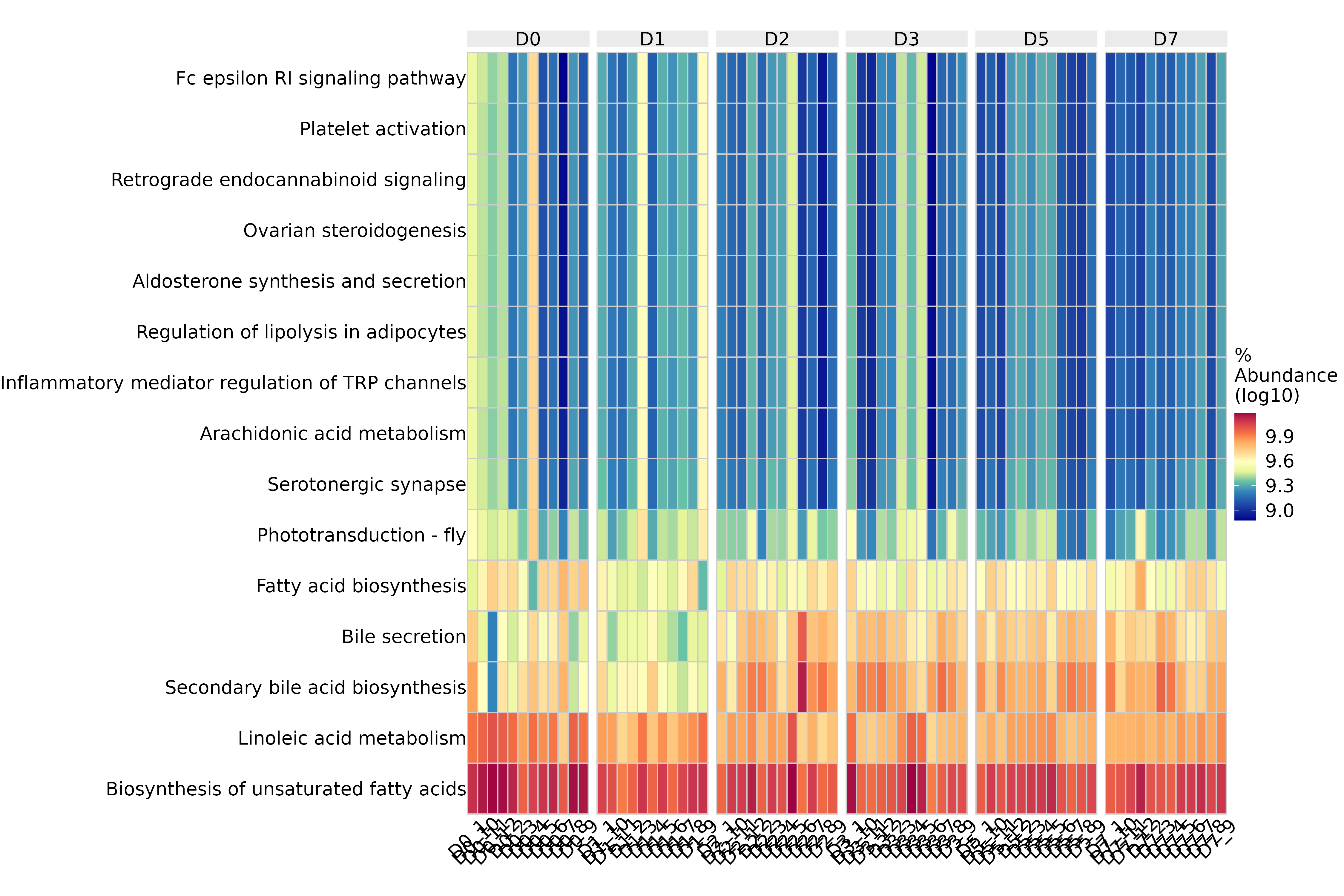

Supplement: Supplementary file 3 — Additional file 3. Raw data of the metabolomic compounds. [file 40104_2026_1385_MOESM3_ESM.zip › mix/KEGG_function_summary/Heatmap/function_summary_level3_heatmap.png]
